# Supplementary material for: WRN structural flexibility showcased through fragment-based lead discovery of inhibitors
Source: Nat Commun. 2026 Jan 3;17:79. doi: 10.1038/s41467-025-66768-8 (PMC12770528; doi:10.1038/s41467-025-66768-8)
Supplement: Supplementary file 1 — Supplementary Information [file 41467_2025_66768_MOESM1_ESM.pdf]

WRN structural flexibility showcased through fragment-based lead discovery of inhibitors.

Rachel L. Palte[1‡], Mihir Mandal[2‡], Justyna Sikorska[3‡], Artjohn B. Villafania[3‡], Meredith Rickard[1], Robert J. Bauer[4], Alexei V. Buevich[5], Xiaomei Chai[4], Jiafang He[2], Zahid Hussain[2], Markus Koglin[3], Hannah B. MacDonald[6], My S. Mansueto[4], Klaus Maskos[7], Joey L. Methot[1], Jaclyn Robustelli[3], Aileen Soriano,[3] Marcel J. Tauchert[7], Sriram Tyagarajan[4], Minjia Zhang[4], Daniel J. Klein[8], Jacqueline D. Hicks[2], David G. McLaren[3], Sandra B. Gabelli[8]*, Daniel F. Wyss[3]*

[1]Discovery Chemistry, Merck & Co., Inc., MRL, Boston, MA 02115, USA
[2]Discovery Chemistry, Merck & Co., Inc., MRL, Rahway, NJ 07065, USA
[3]Quantitative Biosciences, Merck & Co., Inc., MRL, Rahway, NJ 07065, USA
[4]Quantitative Biosciences, Merck & Co., Inc., MRL, Boston, MA 02115, USA
[5]Analytical Research & Development, Merck & Co., Inc., Rahway, NJ 07065, USA
[6]Discovery Chemistry London, MSD (UK) Ltd., London, UK
[7]Proteros Biostructures Germany
[8]Discovery Chemistry West Point, Merck & Co., Inc., MRL, West Point, PA 19486, USA
‡Shared first authors
*Shared corresponding authors

Figure SI 1. Workflows describing the bioNMR and SPR screening arms of fragment libraries.

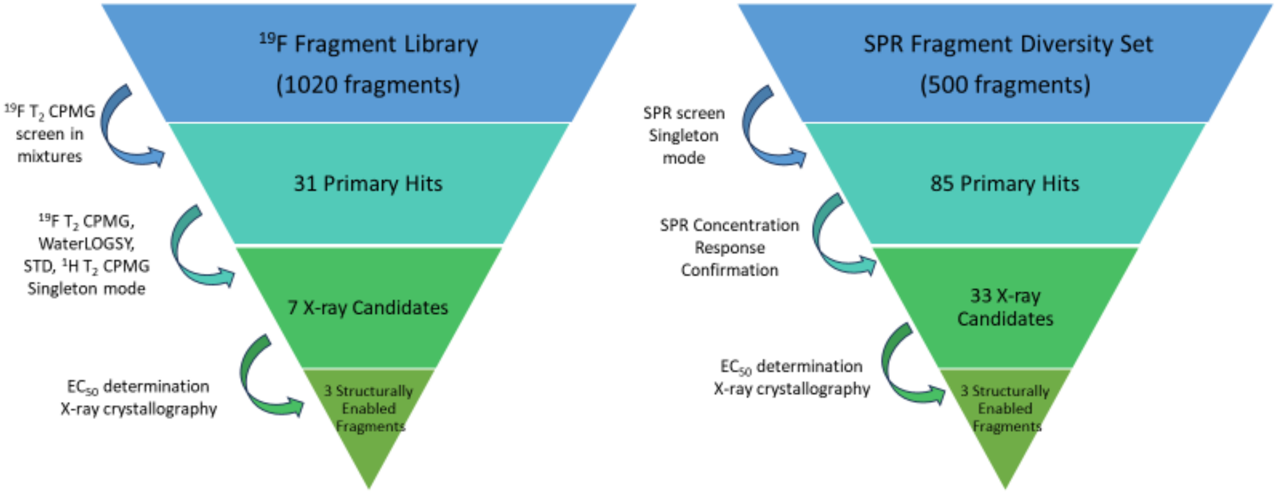

Figure SI 2. The SDS-PAGE gels for proteins used in biochemical, biophysical, and structural studies. A) HIS-FLG-THB-(WRN 500-942); B) GST-PRE-GP-(WRN 500-946)-AVI. Source data are provided as a Source Data file.

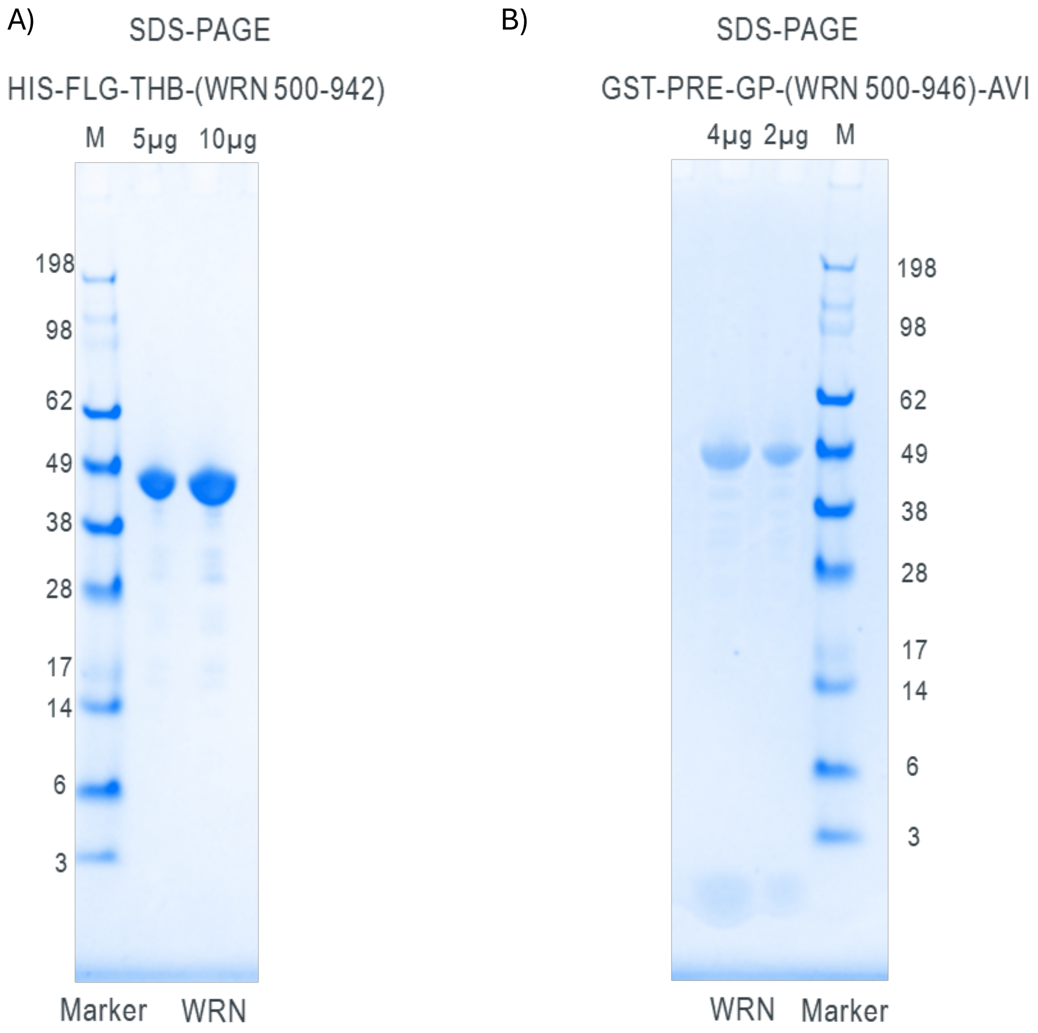

Figure SI 3. NMR data for fragment 2 used in the prioritization for x-ray crystallography. All samples were prepared in 25 mM HEPES, 50 mM NaCl, 2 mM MgCl2, pH 7.4 buffer and DMSO-D6 always matched to 2.5% final sample volume. A) **Fragment 2** ^{19}F T_2 CPMG experiment confirming dose dependent interaction with WRN. B) **Fragment 2** binding to WRN in the waterLOGSY, ^{1}H T_2 CPMG and STD experiments placing it among the Rank 1 hit compounds.

A)

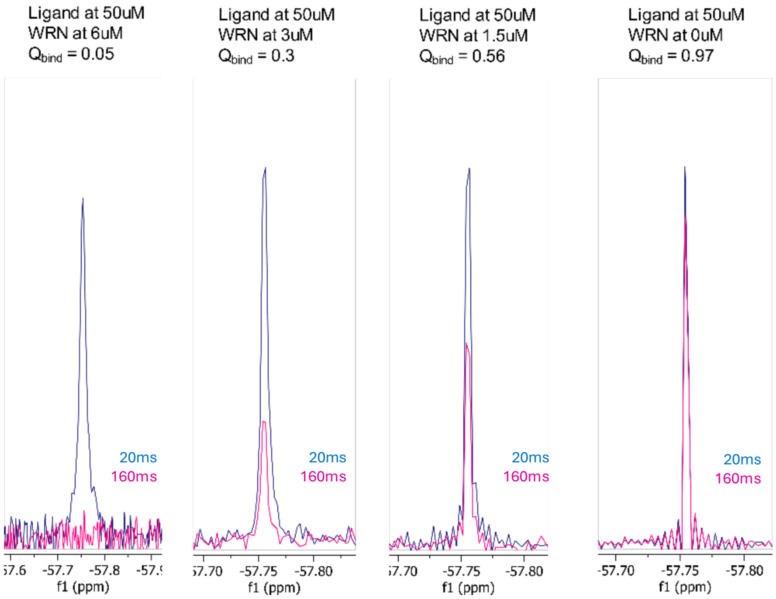

B)

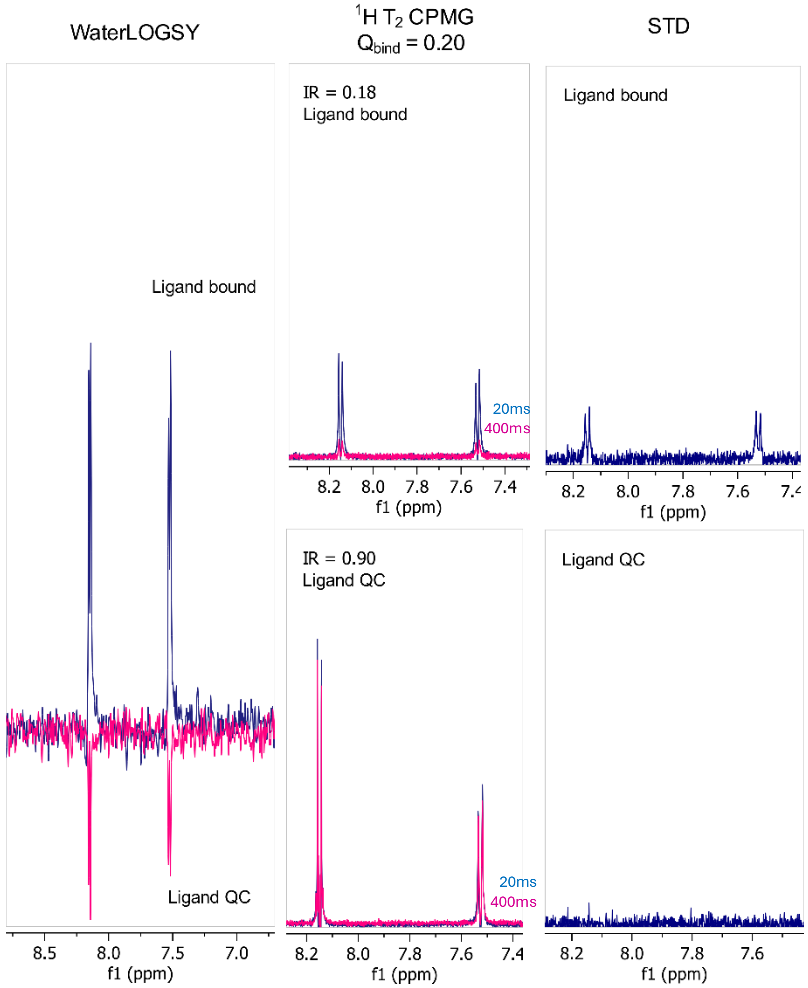
WaterLOGSY
Ligand bound
Ligand QC
8.5 8.0 7.5 7.0
f1 (ppm)
1H T2 CPMG
Qbind = 0.20
IR = 0.18
Ligand bound
20ms
400ms
8.2 8.0 7.8 7.6 7.4
f1 (ppm)
IR = 0.90
Ligand QC
20ms
400ms
8.2 8.0 7.8 7.6 7.4
f1 (ppm)
STD
Ligand bound
8.2 8.0 7.8 7.6
f1 (ppm)
Ligand QC
8.2 8.0 7.8 7.6
f1 (ppm)

Figure SI 4. Biochemical ATPase data for all described compounds. (A) Lead compounds and (B) fragments are shown. EC_{50} values are included in SI Table 1. All data is at least N=2. Source data are provided as a Source Data file.

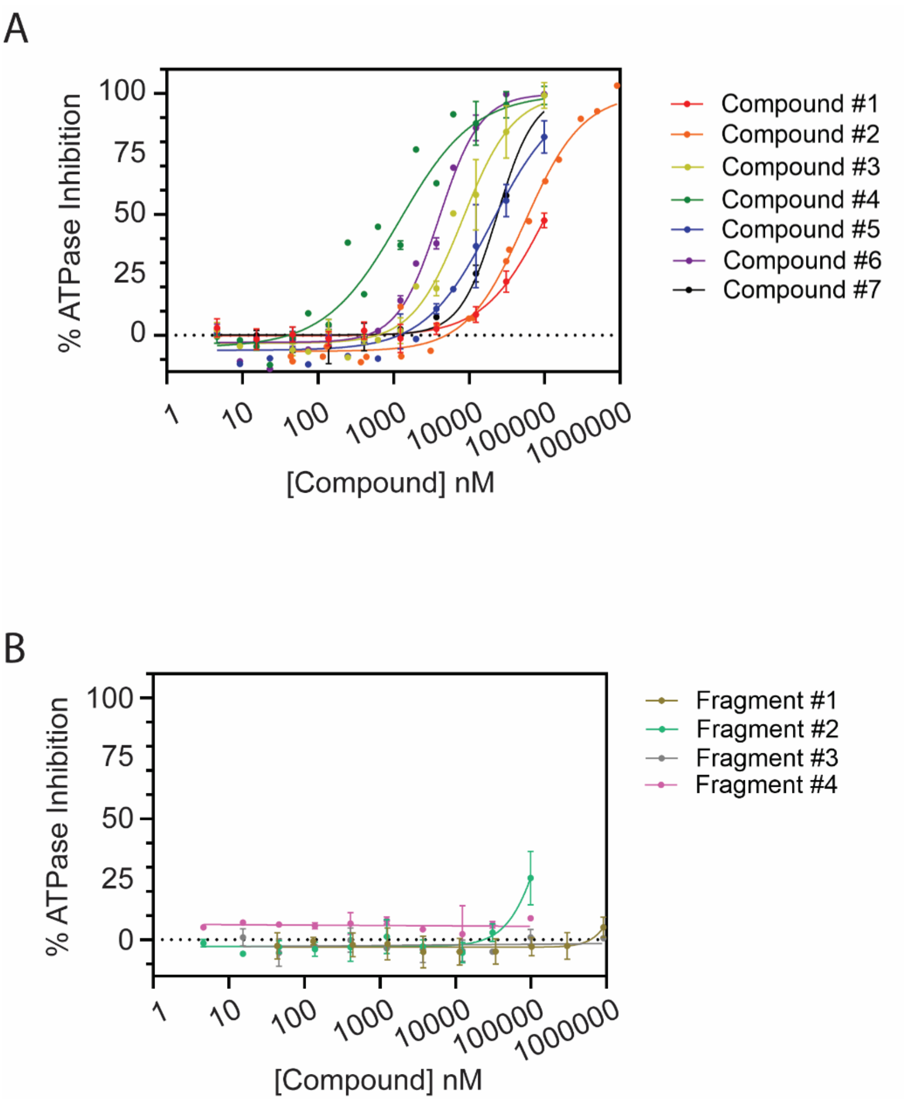

Figure SI 5. Fragments 3 and 4 bind to WRN (500-946). a) **Fragment 3** binding to WRN (500-946) was measured by multi-cycle kinetics using SPR with increasing concentrations (125, 250, 500, 1000 µM). b) Same as for **Fragment 4**. Source data are provided as a Source Data file.

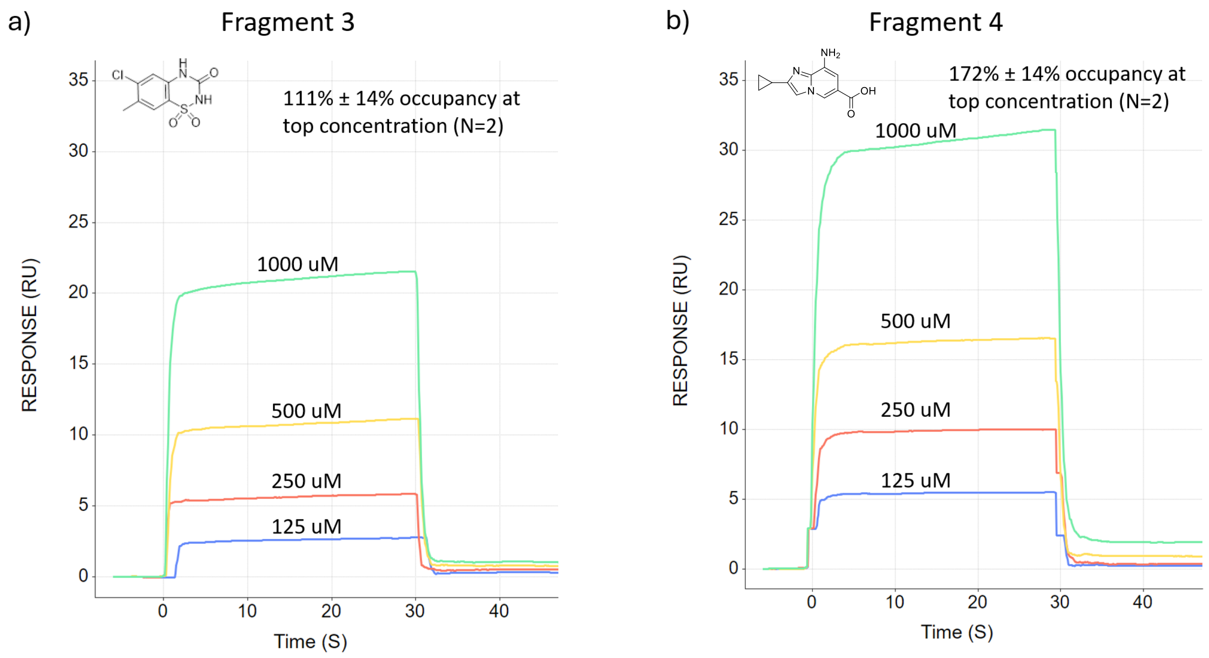

Figure SI 6. Overlay of AMP-PNP-bound WRN with PDB ID 6YHR structure. The structural construct used here (500-942) with AMP-PNP bound (white) and that of a longer published structure with ADP bound (6YHR; residues 517-1093) (brown) overlay with an RMSD of 0.52 Å. The Fo-Fc density for AMP-PNP is shown contoured to 2.5 σ.

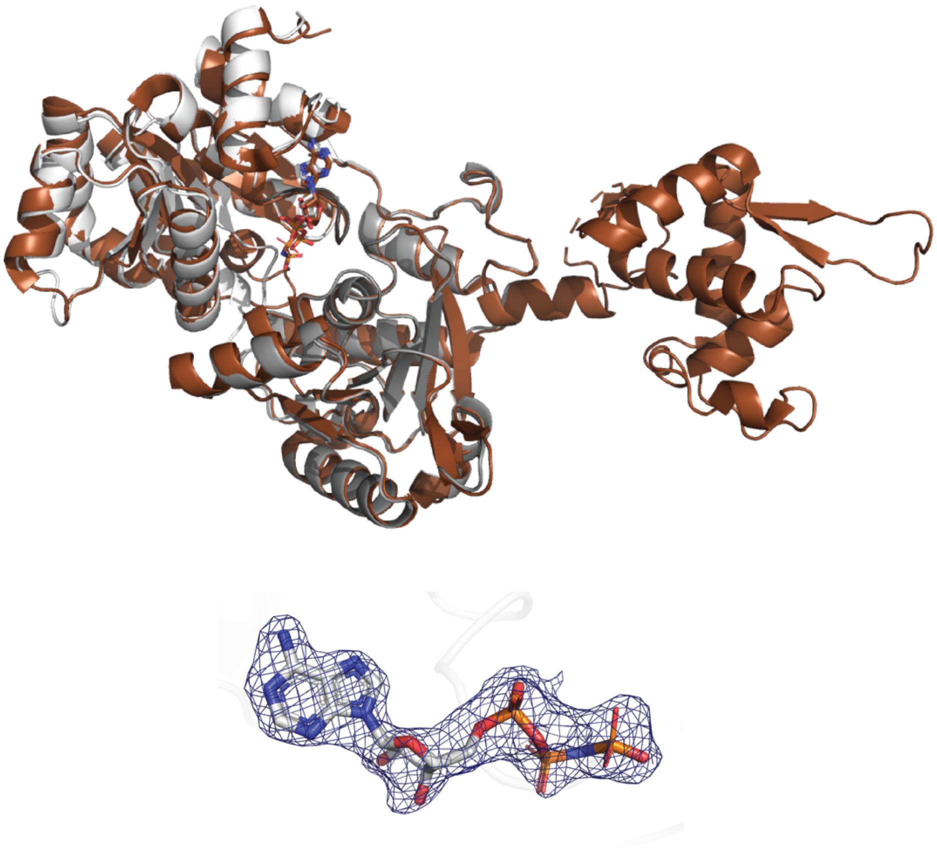

Figure SI 7. Overlay of Form D with bound fragment 1 and Form D in apo form. The WRN Form D can be observed both with ligand (**fragment 1** in blue) and apo form (red).

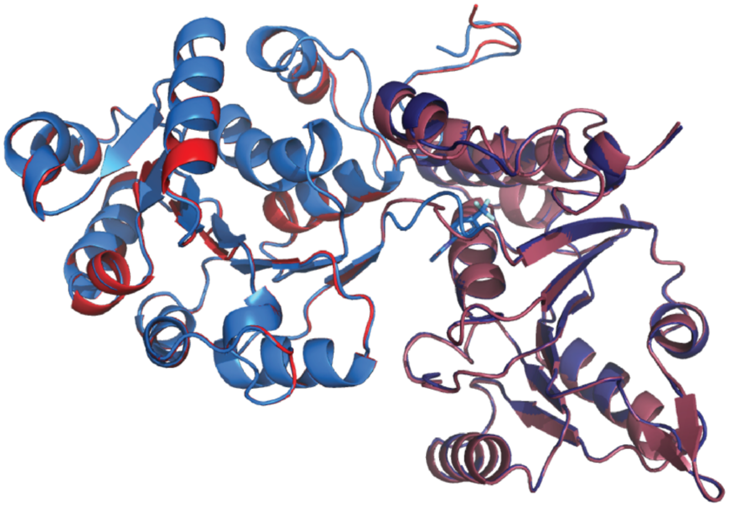

Figure SI 8. A representative SPR sensorgram and where applicable affinity plot for **compounds 2, 3, 4, 5, and 7.** The left panel represents multi-cycle kinetic analysis of **compound 2** and single-cycle kinetic analysis of **compounds 3, 4, 5, and 7** showing response to a 2-fold dilution series of injections over immobilized WRN. The top dose for **compound 2** was 500 µM, while for the remaining compounds, it was 25 µM. The right panel is the respective steady-state affinity fit graph. The binding affinities were calculated from two independent experiments, and the average and range from the steady-state fit are denoted on the affinity plot when fitting conditions have been met. In the case of compound 5 and compound 7, the binding affinity cannot be accurately reported due to low signal and a biphasic binding profile, respectively. Source data are provided as a Source Data file.

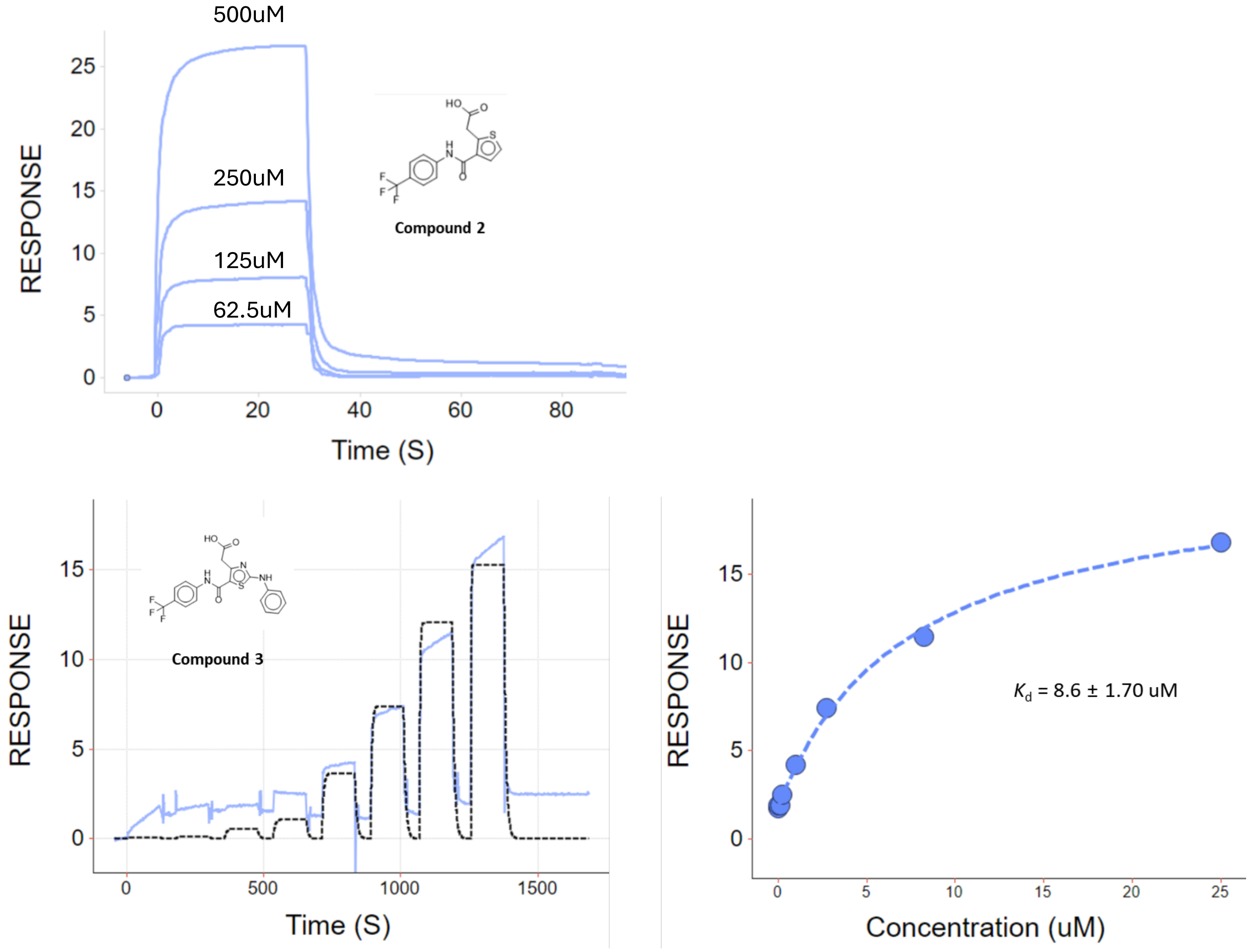

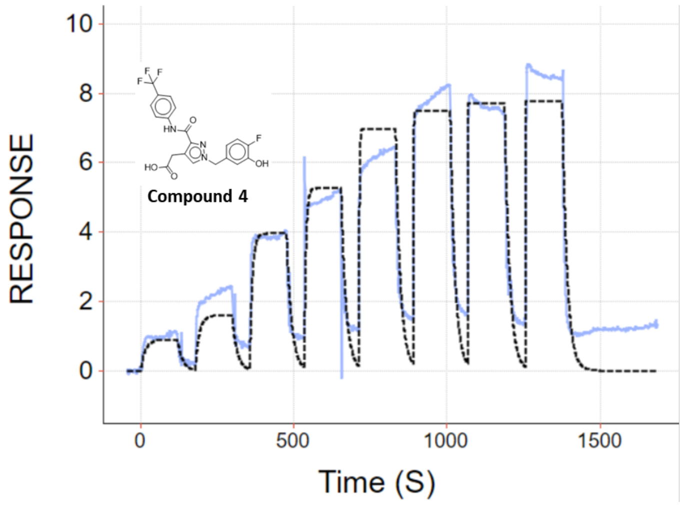
Compound 4
RESPONSE
Time (S)

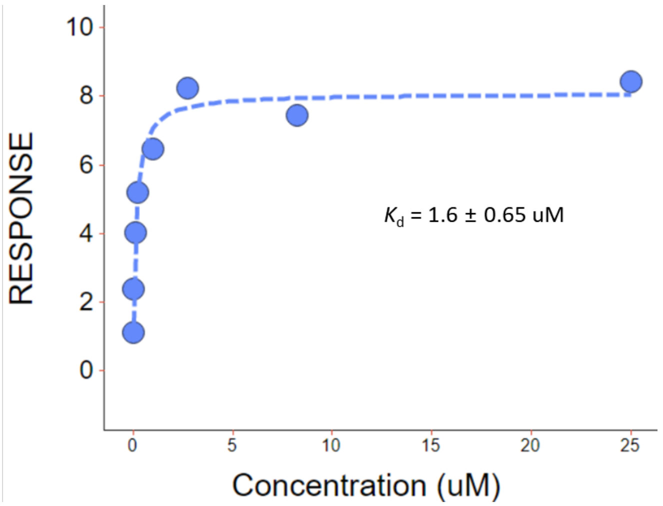
K_d = 1.6 ± 0.65 uM
RESPONSE
Concentration (uM)

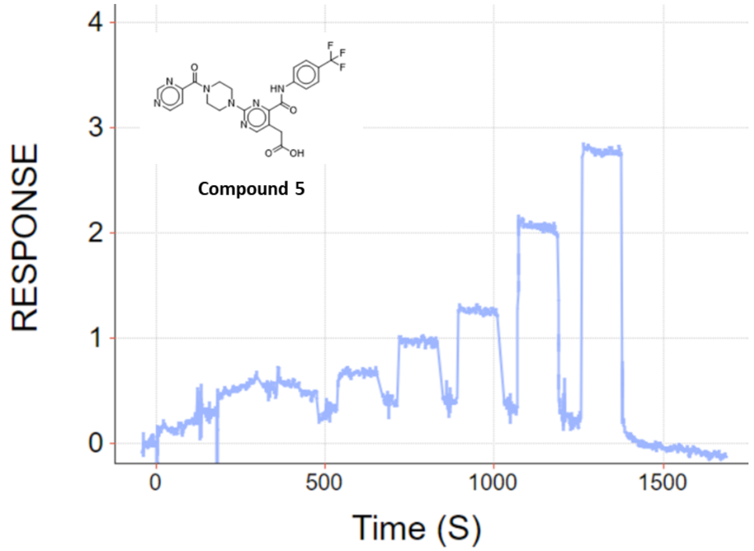
Compound 5
RESPONSE
Time (S)

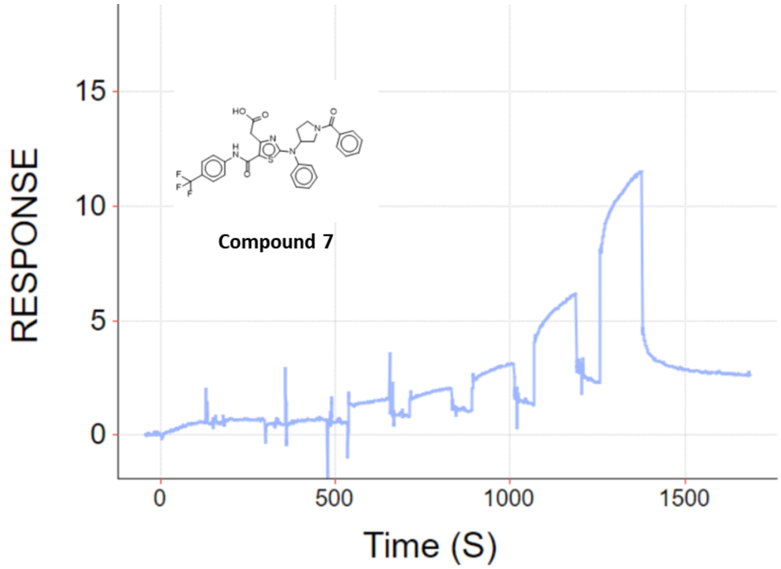
Compound 7
RESPONSE
Time (S)

Figure SI 9. Docking poses of compounds 4, 5, and 7. (A) **Compounds 4** (white bonds) and **5** (light gray bonds) are docked into the structure of WRN with **compound 6** (yellow bonds). (B) **Compound 7** (dark gray bonds) is docked into the structure of WRN with **compound 3** (salmon).

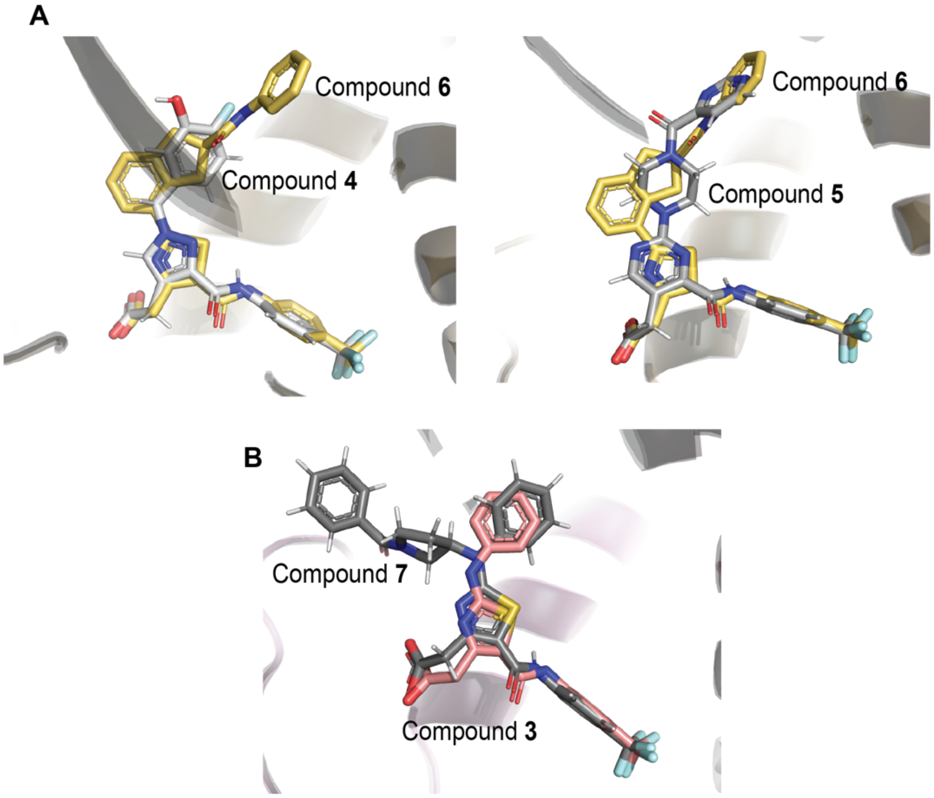

Figure SI 10. Overlay of BLM with WRN-**fragment 1** and WRN-**compound 5**. (A) Overlay of D1 domains of BLM (white), WRN-**fragment 1** (blue,) and WRN-**compound 5** (green) structures. (B) Overlay of the same structures on the D2 domain.

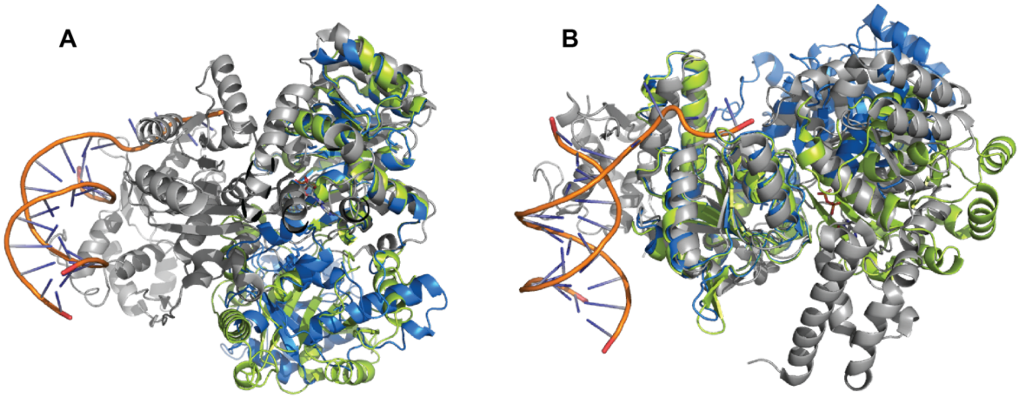

Table SI 1
The Human Werner-Syndrome (WRN) protein used for SPR, X-ray, biochemistry, and bioNMR studies.

Application	Plasmid	Construct Sequence	Amino Acid sequence of expressed construct	Full plasmid DNA-sequence	Insert DNA-sequence
SPR	pFastBac1	GST-PRE-GP-(WRN 500-946)-AVI	MSPILGYWKIKGLVQPTRLLLEYLEEKYEEHLYERDEGDKWRNKKFELGLEFPNLPYYIDGDVKLTQSMAIIRYIADKHNMLGGCPKERAEISMLEGAVLDIRYGVSRIAYSKDFETLKVDFLSKLPEMLKMFEDRLCHKTYLNGDHVTHPDFMLYDALDVVLYMDPMCLDAFPKLVCFKKRIEAIPQIDKYLKSSKYIAWPLQGWQATFGGGDHPPKSDLEVLFQGPNLGLPTKEEEEDDENEANEGEEDDDKDFLWPAPNEEQVTCLKMYFGHSSFKPVQWKVIHSVLEERRDNVAVMATGYGKSLCFQYPPVYVGKIGLVISPLISLMEDQVLQLKMSNIPACFLGSAQSENVLTDIKLGKYRIVYVTPEYCSGNMGLLQQLEADIGITLIAVDEAHCISEWGHDFRDSFRKLGSLKTALPMVPIVALTAT	GACGCGCCCTGTAGCGGCGCATTAAGCGCGGCGGGTGTGGTGGTTACGCGCAGCGTGACCGCTACACTTGCCAGCGCCCTAGCGCCCGCTCCTTTCGCTTTCTTCCCTTCCTTTCTCGCCACGTTCGCCGGCTTTCCCCGTCAAGCTCTAAATCGGGGGCTCCCTTTAGGGTTCCGATTTAGTGCTTTACGGCACCTCGACCCCAAAAAACTTGATTAGGGTGATGGTTCACGTAGTGGGCCATCGCCCTGATAGACGGTTTTTCGCCCTTTGACGTTGGAGTCCACGTTCTTTAATAGTGGACTCTTGTTCCAAACTGGAACAACACTCAACCCTATCTCGGTCTATTCTTTTGATTTATAAGGGATTTTGCCGATTTCGGCCTATTGGTTAAAAAATGAGCTGATTTAACAAAAATTTAACGCGAATTTTAACAAAATATTAACGTTTACAATTTCAGGTGGCACTTTTCGGGGAAATGTGCGCGGAACCCCTATTTGTTTATTTTTCTAAATACATTCAAATATGTATCCGCTCATGAGACAATAACCCTGATAAATGCTTCAATAATATTGAAAAAGGAAGAGTATGAGTATTCAACATTTC	ATGTCCCCTATCCTGGGCTACTGGAAGATCAAGGGACTGGTCCAGCCCACCCGTCTGCTGCTGGAGTACCTGGAGGAAAAGTACGAGGAACACCTGTACGAGCGTGACGAAGGAGACAAGTGGAGGAACAAGAAGTTCGAGCTGGGTCTGGAGTTCCCTAACCTGCCCTACTACATCGACGGCGACGTGAAGCTGACTCAGTCAATGGCTATCATCCGTTACATCGCCGACAAGCACAACATGCTGGGTGGCTGCCCTAAGGAGAGGGCTGAAATCTCCATGCTGGAGGGTGCCGTCCTGGACATCCGTTACGGCGTGAGCAGAATCGCTTACTCTAAGGACTTCGAAACCCTGAAGGTGGACTTCCTGAGCAAGCTGCCTGAGATGCTGAAGATGTTCGAAGACAGACTGTGCCACAAGACCTACCTGAACGGAGACCACGTGACTCACCCCGACTTCATGCTGTACGACGCCCTGGACGTGGTCCTGTACATGGACCCAATGTGCCTGGACGCTTTCCCTAAGCTGGTGTGCTTC

			ASSSIREDIVRCLNLRN<br>PQITCTGFDRPNLYLEV<br>RRKTGNILQDLQPFLVK<br>TSSHWEFEGPTIIYCPS<br>RKMTQQVTGELRKLNL<br>SCGTYHAGMSFSTRKDI<br>HHRFVRDEIQCVIATIAF<br>GMGINKADIRQVIHYGA<br>PKDMESYYQEIGRAGR<br>DGLQSSCHVLWAPADI<br>NLNRHLLTEIRNEKFRL<br>YKLKMMAKMEKYLHSS<br>RCRRQIILSHFEDKQVQ<br>KASLGIMGTEKCCDNC<br>RSRLDHCGSGSGLNDI<br>FEAQKIEWHE	CGTGTCGCCCTTATTCCCTTTTTT<br>GCGGCATTTTGCCTTCCTGTTTTT<br>GCTCACCCAGAAACGCTGGTGA<br>AAGTAAAAGATGCTGAAGATCAGT<br>TGGGTGCACGAGTGGGTTACATC<br>GAACTGGATCTCAACAGCGGTAA<br>GATCCTTGAGAGTTTTCGCCCCG<br>AAGAACGTTTTCCAATGATGAGC<br>ACTTTTAAAGTTCTGCTATGTGGC<br>GCGGTATTATCCCGTATTGACGC<br>CGGGCAAGAGCAACTCGGTCG<br>CCGCATACACTATTCTCAGAATGA<br>CTTGGTTGAGTACTCACCAGTCA<br>CAGAAAAGCATCTTACGGATGGC<br>ATGACAGTAAGAGAATTATGCAGT<br>GCTGCCATAACCATGAGTGATAA<br>CACTGCGGCCAACTTACTTCTGA<br>CAACGATCGGAGGACCGAAGGA<br>GCTAACCGCTTTTTTGCACAACAT<br>GGGGGATCATGTAACTCGCCTTG<br>ATCGTTGGGAACCGGAGCTGAAT<br>GAAGCCATACCAAACGACGAGC<br>GTGACACCACGATGCCTGTAGC<br>AATGGCAACAACGTTGCGCAAA<br>CTATTAACTGGCGAACTACTTACT<br>CTAGCTTCCCGGCAACAATTAAT<br>AGACTGGATGGAGGCGGATAAA<br>GTTGCAGGACCACTTCTGCGCT<br>CGGCCCTTCCGGCTGGCTGGTT<br>TATTGCTGATAAATCTGGAGCCG<br>GTGAGCGTGGGTCTCGCGGTAT	AAGAAGAGGATCGAGGCCAT<br>CCCACAGATCGACAAGTACCT<br>GAAGTCCAGCAAGTACATCGC<br>TTGGCCTCTGCAGGGTTGGC<br>AAGCTACCTTCGGAGGTGGC<br>GACCACCCTCCCAAGTCTGA<br>CCTGGAGGTCCTGTTCCAGG<br>GTCCCAACCTGGGCCTGCCA<br>ACTAAGGAAGAGGAAGAGGA<br>CGACGAGAACGAAGCTAACG<br>AAGGAGAGGAAGACGACGAC<br>AAGGACTTCCTGTGGCCCGC<br>CCCAAACGAGGAACAGGTGA<br>CCTGCCTGAAGATGTACTTCG<br>GCCACTCTTCATTCAAGCCCG<br>TGCAGTGGAAGGTCATCCACA<br>GCGTGCTGGAGGAACGCCGT<br>GACAACGTGGCTGTCATGGC<br>CACTGGATACGGCAAGTCCCT<br>GTGCTTCCAGTACCCACCTGT<br>GTACGTCGGCAAGATCGGCC<br>TGGTCATCTCACCCCTGATCT<br>CCCTGATGGAGGACCAGGTG<br>CTGCAGCTGAAGATGAGCAA<br>CATCCCAGCTTGCTTCCTGGG<br>TAGCGCCCAGTCTGAAAACGT<br>CCTGACCGACATCAAGCTGG<br>GCAAGTACCGTATCGTGTACG<br>TCACTCCAGAGTACTGCAGCG<br>GAAACATGGGTCTGCTGCAG<br>CAGCTGGAAGCTGACATCGG

				CATTGCAGCACTGGGGCCAGAT<br>GGTAAGCCCTCCCGTATCGTAGT<br>TATCTACACGACGGGGAGTCAG<br>GCAACTATGGATGAACGAAATAG<br>ACAGATCGCTGAGATAGGTGCCT<br>CACTGATTAAGCATTGGTAACTGT<br>CAGACCAAGTTTACTCATATATAC<br>TTTAGATTGATTTAAAACTTCATTTT<br>TAATTTAAAAGGATCTAGGTGAAG<br>ATCCTTTTTGATAATCTCATGACCA<br>AAATCCCTTAACGTGAGTTTTCGT<br>TCCACTGAGCGTCAGACCCCGT<br>AGAAAAGATCAAAGGATCTTCTTG<br>AGATCCTTTTTTTCTGCGCGTAAT<br>CTGCTGCTTGCAAACAAAAAAAC<br>CACCGCTACCAGCGGTGGTTTG<br>TTTGCCGGATCAAGAGCTACCAA<br>CTCTTTTTCCGAAGGTAACTGGCT<br>TCAGCAGAGCGCAGATACCAAA<br>TACTGTCCTTCTAGTGTAGCCGTA<br>GTTAGGCCACCACTTCAAGAACT<br>CTGTAGCACCGCCTACATACCTC<br>GCTCTGCTAATCCTGTTACCAGT<br>GGCTGCTGCCAGTGGCGATAAG<br>TCGTGTCTTACCGGGTTGGACTC<br>AAGACGATAGTTACCGGATAAGG<br>CGCAGCGGTCGGGCTGAACGG<br>GGGGTTCGTGCACACAGCCCAG<br>CTTGGAGCGAACGACCTACACC<br>GAACTGAGATACCTACAGCGTGA<br>GCATTGAGAAAGCGCCACGCTT	CATCACCCTGATCGCTGTGGA<br>CGAGGCCCACTGCATCTCTG<br>AATGGGGCCACGACTTCCGC<br>GACTCATTCCGTAAGCTGGGT<br>TCCCTGAAGACTGCTCTGCCA<br>ATGGTGCCTATCGTCGCCCTG<br>ACCGCTACTGCCTCCAGCTCT<br>ATCCGCGAGGACATCGTCCG<br>TTGCCTGAACCTGAGGAACC<br>CCCAGATCACCTGCACTGGAT<br>TCGACCGCCCAAACCTGTAC<br>CTGGAAGTCAGGAGAAAGAC<br>CGGTAACATCCTGCAGGACCT<br>GCAGCCCTTCCTGGTGAAGA<br>CCTCATCCCACTGGGAGTTCG<br>AAGGCCCTACTATCATCTACTG<br>CCCCTCAAGAAAGATGACCC<br>AGCAAGTGACTGGAGAGCTG<br>CGCAAGCTGAACCTGTCCTG<br>CGGAACCTACCACGCTGGTAT<br>GAGCTTCTCTACTCGTAAGGA<br>CATCCACCACCGCTTCGTCC<br>GTGACGAAATCCAGTGCGTGA<br>TCGCTACTATCGCCTTCGGCA<br>TGGGAATCAACAAGGCTGACA<br>TCAGGCAGGTCATCCACTACG<br>GTGCCCCAAAGGACATGGAG<br>TCATACTACCAGGAAATCGGC<br>AGGGCCGGAAGAGACGGTCT<br>GCAGTCCTCCTGCCACGTGC<br>TGTGGGCTCCTGCTGACATCA

				CCCGAAGGGAGAAAGGCGGAC<br>AGGTATCCGGTAAGCGGCAGGG<br>TCGGAACAGGAGAGCGCACGA<br>GGGAGCTTCCAGGGGGAAACG<br>CCTGGTATCTTTATAGTCCTGTCG<br>GGTTTCGCCACCTCTGACTTGAG<br>CGTCGATTTTTGTGATGCTCGTCA<br>GGGGGGCGGAGCCTATGGAAA<br>AACGCCAGCAACGCGGCCTTTT<br>TACGGTTCCTGGCCTTTTGCTGG<br>CCTTTTGCTCACATGTTCTTTCCT<br>GCGTTATCCCCTGATTCTGTGGAT<br>AACCGTATTACCGCCTTTGAGTG<br>AGCTGATACCGCTCGCCGCAGC<br>CGAACGACCGAGCGCAGCGAG<br>TCAGTGAGCGAGGAAGCGGAAG<br>AGCGCCTGATGCGGTATTTTCTC<br>CTTACGCATCTGTGCGGTATTTCA<br>CACCGCAGACCAGCCGCGTAA<br>CCTGGCAAAATCGGTTACGGTTG<br>AGTAATAAATGGATGCCCTGCGT<br>AAGCGGGTGTGGGCGGACAATA<br>AAGTCTTAAACTGAACAAAATAGA<br>TCTAAACTATGACAATAAAGTCTTA<br>AACTAGACAGAATAGTTGTAAACT<br>GAAATCAGTCCAGTTATGCTGTGA<br>AAAAGCATACTGGACTTTTGTTAT<br>GGCTAAAGCAAACTCTTCATTTTC<br>TGAAGTGCAAATTGCCCGTCGTA<br>TTAAAGAGGGGCGTGGCCAAGG<br>GCATGGTAAAGACTATATTCGCG	ACCTGAACAGACACCTGCTGA<br>CCGAGATCAGAAACGAAAAGT<br>TCCGCCTGTACAAGCTGAAGA<br>TGATGGCTAAGATGGAGAAGT<br>ACCTGCACTCATCCCGCTGC<br>CGCCGTCAGATCATCCTGAG<br>CCACTTCGAGGACAAGCAGG<br>TGCAGAAGGCCTCTCTGGGC<br>ATCATGGGAACTGAAAAGTGC<br>TGCGACAACTGCAGGTCCAG<br>ACTGGACCACTGCGGTTCAG<br>GCTCCGGACTGAACGACATCT<br>TCGAGGCTCAGAAGATCGAGT<br>GGCACGAATAATAA

				GCGTTGTGACAATTTACCGAACA ACTCCGCGGCCGGGAAGCCGA TCTCGGCTTGAACGAATTGTTAG GTGGCGGTACTTGGGTCGATATC AAAGTGCATCACTTCTTCCCGTAT GCCCAACTTTGTATAGAGAGCCA CTGCGGGATCGTCACCGTAATCT GCTTGCACGTAGATCACATAAGC ACCAAGCGCGTTGGCCTCATGC TTGAGGAGATTGATGAGCGCGGT GGCAATGCCCTGCCTCCGGTGC TCGCCGGAGACTGCGAGATCAT AGATATAGATCTCACTACGCGGC TGCTCAAACCTGGGCAGAACGT AAGCCGCGAGAGCGCCAACAA CCGCTTCTTGGTCGAAGGCAGC AAGCGCGATGAATGTCTTACTAC GGAGCAAGTTCCCGAGGTAATC GGAGTCCGGCTGATGTTGGGAG TAGGTGGCTACGTCTCCGAACTC ACGACCGAAAAGATCAAGAGCA GCCCGCATGGATTTGACTTGGTC AGGGCCGAGCCTACATGTGCGA ATGATGCCCATACTTGAGCCACC TAACTTTGTTTTAGGGCGACTGCC CTGCTGCGTAACATCGTTGCTGC TGCGTAACATCGTTGCTGCTCCA TAACATCAAACATCGACCCACGG CGTAACGCGCTTGCTGCTTGGAT GCCCGAGGCATAGACTGTACAA AAAAACAGTCATAACAAGCCATG	

				AAAACCGCCACTGCGCCGTTAC<br>CACCGCTGCGTTCGGTCAAGGT<br>TCTGGACCAGTTGCGTGAGCGC<br>ATACGCTACTTGCATTACAGTTTA<br>CGAACCGAACAGGCTTATGTCAA<br>CTGGGTTCGTGCCTTCATCCGTT<br>TCCACGGTGTGCGTCACCCGGC<br>AACCTTGGGCAGCAGCGAAGTC<br>GAGGCATTTCTGTCCTGGCTGGC<br>GAACGAGCGCAAGGTTTCGGTC<br>TCCACGCATCGTCAGGCATTGG<br>CGGCCTTGCTGTTCTTCTACGGC<br>AAGGTGCTGTGCACGGATCTGC<br>CCTGGCTTCAGGAGATCGGAAG<br>ACCTCGGCCGTCGCGGCGCTT<br>GCCGGTGGTGCTGACCCCGGAT<br>GAAGTGGTTCGCATCCTCGGTTT<br>TCTGGAAGGCGAGCATCGTTTGT<br>TCGCCCAGGACTCTAGCTATAGT<br>TCTAGTGGTTGGCTACGTATACTC<br>CGGAATATTAATAGATCATGGAGA<br>TAATTAAAATGATAACCATCTCGC<br>AAATAAATAAGTATTTTACTGTTTTC<br>GTAACAGTTTTGTAATAAAAAAAC<br>CTATAAATATTCCGGATTATTCATA<br>CCGTCCCACCATCGGGCGCGG<br>ATCCGCCACCATGTCCCCTATCC<br>TGGGCTACTGGAAGATCAAGGG<br>ACTGGTCCAGCCCACCCGTCTG<br>CTGCTGGAGTACCTGGAGGAAA<br>AGTACGAGGAACACCTGTACGA	

				GCGTGACGAAGGAGACAAGTGG AGGAACAAGAAGTTCGAGCTGG GTCTGGAGTTCCCTAACCTGCCC TACTACATCGACGGCGACGTGAA GCTGACTCAGTCAATGGCTATCA TCCGTTACATCGCCGACAAGCA CAACATGCTGGGTGGCTGCCCT AAGGAGAGGGCTGAAATCTCCAT GCTGGAGGGTGCCGTCCTGGAC ATCCGTTACGGCGTGAGCAGAAT CGCTTACTCTAAGGACTTCGAAA CCCTGAAGGTGGACTTCCTGAG CAAGCTGCCTGAGATGCTGAAG ATGTTCGAAGACAGACTGTGCCA CAAGACCTACCTGAACGGAGAC CACGTGACTCACCCCGACTTCAT GCTGTACGACGCCCTGGACGTG GTCCTGTACATGGACCCAATGTG CCTGGACGCTTTCCCTAAGCTG GTGTGCTTCAAGAAGAGGATCGA GGCCATCCCACAGATCGACAAG TACCTGAAGTCCAGCAAGTACAT CGCTTGGCCTCTGCAGGGTTGG CAAGCTACCTTCGGAGGTGGCG ACCACCCTCCCAAGTCTGACCT GGAGGTCCTGTTCCAGGGTCCC AACCTGGGCCTGCCAACTAAGG AAGAGGAAGAGGACGACGAGAA CGAAGCTAACGAAGGAGAGGAA GACGACGACAAGGACTTCCTGT GGCCCGCCCCAAACGAGGAAC	

				AGGTGACCTGCCTGAAGATGTAC<br>TTCGGCCACTCTTCATTCAAGCC<br>CGTGCAGTGGAAGGTCATCCAC<br>AGCGTGCTGGAGGAACGCCGTG<br>ACAACGTGGCTGTCATGGCCAC<br>TGGATACGGCAAGTCCCTGTGCT<br>TCCAGTACCCACCTGTGTACGTC<br>GGCAAGATCGGCCTGGTCATCT<br>CACCCCTGATCTCCCTGATGGA<br>GGACCAGGTGCTGCAGCTGAAG<br>ATGAGCAACATCCCAGCTTGCTT<br>CCTGGGTAGCGCCCAGTCTGAA<br>AACGTCCTGACCGACATCAAGCT<br>GGGCAAGTACCGTATCGTGTACG<br>TCACTCCAGAGTACTGCAGCGG<br>AAACATGGGTCTGCTGCAGCAG<br>CTGGAAGCTGACATCGGCATCA<br>CCCTGATCGCTGTGGACGAGGC<br>CCACTGCATCTCTGAATGGGGC<br>CACGACTTCCGCGACTCATTCC<br>GTAAGCTGGGTTCCCTGAAGACT<br>GCTCTGCCAATGGTGCCTATCGT<br>CGCCCTGACCGCTACTGCCTCC<br>AGCTCTATCCGCGAGGACATCGT<br>CCGTTGCCTGAACCTGAGGAAC<br>CCCCAGATCACCTGCACTGGATT<br>CGACCGCCCAAACCTGTACCTG<br>GAAGTCAGGAGAAAGACCGGTA<br>ACATCCTGCAGGACCTGCAGCC<br>CTTCCTGGTGAAGACCTCATCCC<br>ACTGGGAGTTCGAAGGCCCTAC	

				TATCATCTACTGCCCCTCAAGAA<br>AGATGACCCAGCAAGTGACTGG<br>AGAGCTGCGCAAGCTGAACCTG<br>TCCTGCGGAACCTACCACGCTG<br>GTATGAGCTTCTCTACTCGTAAGG<br>ACATCCACCACCGCTTCGTCCG<br>TGACGAAATCCAGTGCGTGATCG<br>CTACTATCGCCTTCGGCATGGGA<br>ATCAACAAGGCTGACATCAGGC<br>AGGTCATCCACTACGGTGCCCC<br>AAAGGACATGGAGTCATACTACC<br>AGGAAATCGGCAGGGCCGGAA<br>GAGACGGTCTGCAGTCCTCCTG<br>CCACGTGCTGTGGGCTCCTGCT<br>GACATCAACCTGAACAGACACCT<br>GCTGACCGAGATCAGAAACGAA<br>AAGTTCCGCCTGTACAAGCTGAA<br>GATGATGGCTAAGATGGAGAAGT<br>ACCTGCACTCATCCCGCTGCCG<br>CCGTCAGATCATCCTGAGCCACT<br>TCGAGGACAAGCAGGTGCAGAA<br>GGCCTCTCTGGGCATCATGGGA<br>ACTGAAAAGTGCTGCGACAACTG<br>CAGGTCCAGACTGGACCACTGC<br>GGTTCAGGCTCCGGACTGAACG<br>ACATCTTCGAGGCTCAGAAGATC<br>GAGTGGCACGAATAATAACTCGA<br>GGCATGCGGTACCAAGCTTGTC<br>GAGAAGTACTAGAGGATCATAATC<br>AGCCATACCACATTTGTAGAGGTT<br>TTACTTGCTTTAAAAAACCTCCCA	

				CACCTCCCCCTGAACCTGAAAC ATAAAATGAATGCAATTGTTGTTGT TAACTTGTTTATTGCAGCTTATAAT GGTTACAAATAAAGCAATAGCATC ACAAATTTCACAAATAAAGCATTTT TTTCACTGCATTCTAGTTGTGGTTT GTCCAAACTCATCAATGTATCTTAT CATGTCTGGATCTGATCACTGCTT GAGCCTAGGAGATCCGAACCAG ATAAGTGAAATCTAGTTCCAAACT ATTTTGTCATTTTTAATTTTCGTATTA GCTTACGACGCTACACCCAGTTC CCATCTATTTTGTCACTCTTCCCTA AATAATCCTTAAAAACTCCATTTC CACCCCTCCCAGTTCCCAACTAT TTTGTCCGCCCACAGCGGGGCA TTTTTCTTCCTGTTATGTTTTTAATC AAACATCCTGCCAACTCCATGTG ACAAACCGTCATCTTCGGCTACT TTTTCTCTGTCACAGAATGAAAATT TTTCTGTCATCTCTTCGTTATTAAT GTTTGTAATTGACTGAATATCAAC GCTTATTTGCAGCCTGAATGGCG AATGG	
X-ray	pFastBac1	HIS-FLG-THB-(WRN 500-942)	MHHHHHHHHGSGSD YKDDDDKGSGSLVPRG STSNLGLPTKEEEEDDE NEANEGEEDDDKDFL WPAPNEEQVTCLKMYF GHSSFKPVQWKVIHSV LEERRDNVAVMATGYG	GACGCGCCCTGTAGCGGCGCA TTAAGCGCGGCGGGTGTGGTGG TTACGCGCAGCGTGACCGCTAC ACTTGCCAGCGCCCTAGCGCC CGCTCCTTTCGCTTTCTTCCCTTC CTTTCTCGCCACGTTCGCCGGC TTTCCCCGTCAAGCTCTAAATCG	ATGCACCACCATCATCATCAC CATCACGGTTCCGGTTCCGA CTACAAGGACGACGACGACA AAGGTTCCGGCTCTCTGGTGC CTCGTGGTTCCACTTCTAACCT GGGCCTGCCTACCAAAGAGG AAGAAGAGGACGACGAGAAC

			KSLCFQYPPVYVGKIGL<br>VISPLISLMEDQVLQLK<br>MSNIPACFLGSAQSEN<br>VLTDIKLGKYRIVYVTPE<br>YCSGNMGLLQQLEADI<br>GITLIAVDEAHCISEWG<br>HDFRDSFRKLGSLKTAL<br>PMVPIVALTATASSSIRE<br>DIVRCLNLRNPQITCTG<br>FDRPNLYLEVRRKTGNI<br>LQDLQPFLVKTSSHWE<br>FEGPTIIYCPSRKMTQQ<br>VTGELRKLNLSCGTYHA<br>GMSFSTRKDIHHRFVR<br>DEIQCVIATIAFGMGINK<br>ADIRQVIHYGAPKDMES<br>YYQEIGRAGRDGLQSS<br>CHVLWAPADINLNRHL<br>LTEIRNEKFRLYKLKMM<br>AKMEKYLHSSRCRRQII<br>LSHFEDKQVQKASLGI<br>MGTEKCCDNCRSR	GGGGCTCCCTTTAGGGTTCCGAT<br>TTAGTGCTTTACGGCACCTCGAC<br>CCCAAAAAACTTGATTAGGGTGA<br>TGGTTCACGTAGTGGGCCATCG<br>CCCTGATAGACGGTTTTTCGCCC<br>TTTGACGTTGGAGTCCACGTTCTT<br>TAATAGTGGACTCTTGTTCCAAAC<br>TGGAACAACACTCAACCCTATCT<br>CGGTCTATTCTTTTGATTTATAAGG<br>GATTTTGCCGATTTCGGCCTATTG<br>GTTAAAAAATGAGCTGATTTAACA<br>AAAATTTAACGCGAATTTTAACAA<br>AATATTAACGCTTACAATTTAGGTG<br>GCACTTTTCGGGGAAATGTGCG<br>CGGAACCCCTATTTGTTTATTTTTC<br>TAAATACATTCAAATATGTATCCGC<br>TCATGAGACAATAACCCTGATAAA<br>TGCTTCAATAATATTGAAAAAGGA<br>AGAGTATGAGTATTCAACATTTCC<br>GTGTCGCCCTTATTCCCTTTTTTG<br>CGGCATTTTGCCTTCCTGTTTTTG<br>CTCACCCAGAAACGCTGGTGAA<br>AGTAAAAGATGCTGAAGATCAGTT<br>GGGTGCACGAGTGGGTTACATC<br>GAACTGGATCTCAACAGCGGTAA<br>GATCCTTGAGAGTTTTCGCCCCG<br>AAGAACGTTTTCCAATGATGAGC<br>ACTTTTAAAGTTCTGCTATGTGGC<br>GCGGTATTATCCCGTATTGACGC<br>CGGGCAAGAGCAACTCGGTCG<br>CCGCATACACTATTCTCAGAATGA	GAGGCTAACGAGGGCGAAGA<br>GGATGATGACAAGGACTTCCT<br>GTGGCCTGCTCCTAACGAGG<br>AACAAGTGACCTGCCTGAAGA<br>TGTACTTCGGTCACTCCAGCTT<br>CAAGCCCGTGCAGTGGAAAG<br>TGATCCACTCCGTGCTGGAAG<br>AACGTCGTGACAACGTGGCC<br>GTGATGGCTACCGGTTACGGA<br>AAGTCCCTGTGCTTCCAGTAT<br>CCTCCTGTGTACGTGGGCAAG<br>ATCGGCCTGGTCATCTCCCCT<br>CTGATCTCCCTGATGGAAGAT<br>CAGGTGCTGCAGCTGAAGAT<br>GAGCAATATCCCCGCTTGCTT<br>CCTGGGTTCCGCTCAGTCTGA<br>GAACGTGCTGACCGACATCAA<br>GCTGGGCAAGTACCGTATCGT<br>GTACGTGACCCCTGAGTACTG<br>CTCCGGCAACATGGGACTGC<br>TCCAGCAGCTGGAAGCTGAC<br>ATCGGTATCACCCTGATCGCT<br>GTGGACGAGGCTCACTGCAT<br>CTCTGAGTGGGGTCACGACTT<br>CCGTGACAGCTTCCGCAAGC<br>TGGGTTCTCTCAAGACCGCTC<br>TGCCCATGGTGCCTATCGTGG<br>CTCTGACCGCTACCGCTTCAT<br>CCTCTATCCGCGAGGACATC<br>GTGCGTTGCCTGAACCTGCGT<br>AACCCTCAGATCACTTGCACC

				CTTGGTTGAGTACTCACCAGTCA<br>CAGAAAAGCATCTTACGGATGGC<br>ATGACAGTAAGAGAATTATGCAGT<br>GCTGCCATAACCATGAGTGATAA<br>CACTGCGGCCAACTTACTTCTGA<br>CAACGATCGGAGGACCGAAGGA<br>GCTAACCGCTTTTTTGCACAACAT<br>GGGGGATCATGTAACTCGCCTTG<br>ATCGTTGGGAACCGGAGCTGAAT<br>GAAGCCATACCAAACGACGAGC<br>GTGACACCACGATGCCTGTAGC<br>AATGGCAACAACGTTGCGCAAA<br>CTATTAACTGGCGAACTACTTACT<br>CTAGCTTCCCGGCAACAATTAAT<br>AGACTGGATGGAGGCGGATAAA<br>GTTGCAGGACCACTTCTGCGCT<br>CGGCCCTTCCGGCTGGCTGGTT<br>TATTGCTGATAAATCTGGAGCCG<br>GTGAGCGTGGGTCTCGCGGTAT<br>CATTGCAGCACTGGGGCCAGAT<br>GGTAAGCCCTCCCGTATCGTAGT<br>TATCTACACGACGGGGAGTCAG<br>GCAACTATGGATGAACGAAATAG<br>ACAGATCGCTGAGATAGGTGCCT<br>CACTGATTAAGCATTGGTAACTGT<br>CAGACCAAGTTTACTCATATATAC<br>TTTAGATTGATTTAAAACTTCATTTT<br>TAATTTAAAAGGATCTAGGTGAAG<br>ATCCTTTTTGATAATCTCATGACCA<br>AAATCCCTTAACGTGAGTTTTCGT<br>TCCACTGAGCGTCAGACCCCGT	GGTTTCGACCGTCCTAACCTG<br>TACCTGGAAGTGCGTCGCAA<br>GACCGGAAACATCCTCCAGG<br>ACCTGCAGCCTTTCCTGGTCA<br>AGACCTCTAGCCACTGGGAGT<br>TCGAGGGTCCTACCATCATCT<br>ACTGCCCCTCTCGCAAGATGA<br>CCCAGCAAGTGACTGGCGAG<br>CTGCGCAAGCTGAACCTGTC<br>CTGCGGTACTTACCACGCTGG<br>CATGTCTTTCAGCACCCGCAA<br>GGACATCCACCACCGTTTCGT<br>GCGTGACGAGATCCAGTGCG<br>TGATCGCTACTATCGCTTTCGG<br>CATGGGTATCAACAAGGCTGA<br>CATCCGTCAAGTCATCCACTA<br>CGGTGCTCCCAAGGACATGG<br>AATCCTACTACCAAGAGATCG<br>GTCGTGCTGGTCGTGACGGC<br>CTGCAATCTTCTTGCCACGTGT<br>TGTGGGCTCCCGCTGACATCA<br>ACCTGAACCGTCACCTCCTGA<br>CCGAGATCCGTAACGAGAAGT<br>TCCGTCTGTACAAGCTCAAGA<br>TGATGGCCAAGATGGAAAAGT<br>ACCTGCACTCCTCTCGTTGCC<br>GTCGTCAGATCATCCTGTCTCA<br>CTTCGAGGACAAGCAGGTCC<br>AGAAGGCTTCCCTGGGTATCA<br>TGGGCACCGAGAAGTGCTGC

				AGAAAAGATCAAAGGATCTTCTTG AGATCCTTTTTTTCTGCGCGTAAT CTGCTGCTTGCAAACAAAAAAAC CACCGCTACCAGCGGTGGTTTG TTTGCCGGATCAAGAGCTACCAA CTCTTTTTCCGAAGGTAACTGGCT TCAGCAGAGCGCAGATACCAAA TACTGTTCTTCTAGTGTAGCCGTA GTTAGGCCACCACTTCAAGAACT CTGTAGCACCGCCTACATACCTC GCTCTGCTAATCCTGTTACCAGT GGCTGCTGCCAGTGGCGATAAG TCGTGTCTTACCGGGTTGGACTC AAGACGATAGTTACCGGATAAGG CGCAGCGGTCGGGCTGAACGG GGGGTTCGTGCACACAGCCCAG CTTGGAGCGAACGACCTACACC GAACTGAGATACCTACAGCGTGA GCTATGAGAAAGCGCCACGCTT CCCGAAGGGAGAAAGGCGGAC AGGTATCCGGTAAGCGGCAGGG TCGGAACAGGAGAGCGCACGA GGGAGCTTCCAGGGGGAAACG CCTGGTATCTTTATAGTCCTGTCG GGTTTCGCCACCTCTGACTTGAG CGTCGATTTTTGTGATGCTCGTCA GGGGGGCGGAGCCTATGGAAA AACGCCAGCAACGCGGCCTTTT TACGGTTCCTGGCCTTTTGCTGG CCTTTTGCTCACATGTTCTTTCCT GCGTTATCCCCTGATTCTGTGGAT	GACAACTGCCGTTCTCGCTAA TAA

				AACCGTATTACCGCCTTTGAGTG<br>AGCTGATACCGCTCGCCGCAGC<br>CGAACGACCGAGCGCAGCGAG<br>TCAGTGAGCGAGGAAGCGGAAG<br>AGCGCCTGATGCGGTATTTTCTC<br>CTTACGCATCTGTGCGGTATTTCA<br>CACCGCAAGACCAGCCGCGTA<br>ACCTGGCAAAATCGGTTACGGTT<br>GAGTAATAAATGGATGCCCTGCG<br>TAAGCGGGTGTGGGCGGACAAT<br>AAAGTCTTAAACTGAACAAAATAG<br>ATCTAAACTATGACAATAAAGTCTT<br>AAACTAGACAGAATAGTTGTAAAC<br>TGAAATCAGTCCAGTTATGCTGTG<br>AAAAAGCATACTGGACTTTTGTTA<br>TGGCTAAAGCAAACTCTTCATTTT<br>CTGAAGTGCAAATTGCCCGTCGT<br>ATTAAAGAGGGGCGTGGCCAAG<br>GGCATGGTAAAGACTATATTCGC<br>GGCGTTGTGACAATTTACCGAAC<br>AACTCCGCGGCCGGGAAGCCG<br>ATCTCGGCTTGAACGAATTGTTAG<br>GTGGCGGTACTTGGGTCGATATC<br>AAAGTGCATCACTTCTTCCCGTAT<br>GCCCAACTTTGTATAGAGAGCCA<br>CTGCGGGATCGTCACCGTAATCT<br>GCTTGCACGTAGATCACATAAGC<br>ACCAAGCGCGTTGGCCTCATGC<br>TTGAGGAGATTGATGAGCGCGGT<br>GGCAATGCCCTGCCTCCGGTGC<br>TCGCCGGAGACTGCGAGATCAT	

				AGATATAGATCTCACTACGCGGC<br>TGCTCAAACTTGGGCAGAACGTA<br>AGCCGCGAGAGCGCCAACAAC<br>CGCTTCTTGGTCGAAGGCAGCA<br>AGCGCGATGAATGTCTTACTACG<br>GAGCAAGTTCCCGAGGTAATCG<br>GAGTCCGGCTGATGTTGGGAGTA<br>GGTGGCTACGTCTCCGAACTCA<br>CGACCGAAAAGATCAAGAGCAG<br>CCCGCATGGATTTGACTTGGTCA<br>GGGCCGAGCCTACATGTGCGAA<br>TGATGCCCATACTTGAGCCACCT<br>AACTTTGTTTTAGGGCGACTGCC<br>CTGCTGCGTAACATCGTTGCTGC<br>TGCGTAACATCGTTGCTGCTCCA<br>TAACATCAAACATCGACCCACGG<br>CGTAACGCGCTTGCTGCTTGGAT<br>GCCCGAGGCATAGACTGTACAA<br>AAAAACAGTCATAACAAGCCATG<br>AAAACCGCCACTGCGCCGTTAC<br>CACCGCTGCGTTCGGTCAAGGT<br>TCTGGACCAGTTGCGTGAGCGC<br>ATACGCTACTTGCATTACAGTTTA<br>CGAACCGAACAGGCTTATGTCAA<br>CTGGGTTCGTGCCTTCATCCGTT<br>TCCACGGTGTGCGTCACCCGGC<br>AACCTTGGGCAGCAGCGAAGTC<br>GAGGCATTTCTGTCCTGGCTGGC<br>GAACGAGCGCAAGGTTTCGGTC<br>TCCACGCATCGTCAGGCATTGG<br>CGGCCTTGCTGTTCTTCTACGGC	

				AAGGTGCTGTGCACGGATCTGC<br>CCTGGCTTCAGGAGATCGGAAG<br>ACCTCGGCCGTCGCGGCGCTT<br>GCCGGTGGTGCTGACCCCGGAT<br>GAAGTGGTTCGCATCCTCGGTTT<br>TCTGGAAGGCGAGCATCGTTTGT<br>TCGCCCAGGACTCTAGCTATAGT<br>TCTAGTGGTTGGCTACGTATACTC<br>CGGAATATTAATAGATCATGGAGA<br>TAATTAAAATGATAACCATCTCGC<br>AAATAAATAAGTATTTTACTGTTTTC<br>GTAACAGTTTTGTAATAAAAAAAC<br>CTATAAATATTCCGGATTATTCATA<br>CCGTCCCACCATCGGGCGCGG<br>ATCCGCCACCATGCACCACCAT<br>CATCATCACCATCACGGTTCCGG<br>TTCCGACTACAAGGACGACGAC<br>GACAAAGGTTCCGGCTCTCTGGT<br>GCCTCGTGGTTCCACTTCTAACC<br>TGGGCCTGCCTACCAAAGAGGA<br>AGAAGAGGACGACGAGAACGAG<br>GCTAACGAGGGCGAAGAGGATG<br>ATGACAAGGACTTCCTGTGGCCT<br>GCTCCTAACGAGGAACAAGTGA<br>CCTGCCTGAAGATGTACTTCGGT<br>CACTCCAGCTTCAAGCCCGTGC<br>AGTGGAAAGTGATCCACTCCGTG<br>CTGGAAGAACGTCGTGACAACG<br>TGGCCGTGATGGCTACCGGTTAC<br>GGAAAGTCCCTGTGCTTCCAGTA<br>TCCTCCTGTGTACGTGGGCAAGA	

				TCGGCCTGGTCATCTCCCCTCTG ATCTCCCTGATGGAAGATCAGGT GCTGCAGCTGAAGATGAGCAATA TCCCCGCTTGCTTCCTGGGTTCC GCTCAGTCTGAGAACGTGCTGAC CGACATCAAGCTGGGCAAGTAC CGTATCGTGTACGTGACCCCTGA GTACTGCTCCGGCAACATGGGA CTGCTCCAGCAGCTGGAAGCTG ACATCGGTATCACCCTGATCGCT GTGGACGAGGCTCACTGCATCT CTGAGTGGGGTCACGACTTCCG TGACAGCTTCCGCAAGCTGGGTT CTCTCAAGACCGCTCTGCCCAT GGTGCCTATCGTGGCTCTGACC GCTACCGCTTCATCCTCTATCCG CGAGGACATCGTGCGTTGCCTG AACCTGCGTAACCCTCAGATCAC TTGCACCGGTTTCGACCGTCCTA ACCTGTACCTGGAAGTGCGTCG CAAGACCGGAAACATCCTCCAG GACCTGCAGCCTTTCCTGGTCAA GACCTCTAGCCACTGGGAGTTC GAGGGTCCTACCATCATCTACTG CCCCTCTCGCAAGATGACCCAG CAAGTGACTGGCGAGCTGCGCA AGCTGAACCTGTCCTGCGGTACT TACCACGCTGGCATGTCTTTCAG CACCCGCAAGGACATCCACCA CCGTTTCGTGCGTGACGAGATCC AGTGCGTGATCGCTACTATCGCT	

				TTCGGCATGGGTATCAACAAGGC<br>TGACATCCGTCAAGTCATCCACT<br>ACGGTGCTCCCAAGGACATGGA<br>ATCCTACTACCAAGAGATCGGTC<br>GTGCTGGTCGTGACGGCCTGCA<br>ATCTTCTTGCCACGTGTTGTGGG<br>CTCCCGCTGACATCAACCTGAA<br>CCGTCACCTCCTGACCGAGATC<br>CGTAACGAGAAGTTCCGTCTGTA<br>CAAGCTCAAGATGATGGCCAAG<br>ATGGAAAAGTACCTGCACTCCTC<br>TCGTTGCCGTCGTCAGATCATCC<br>TGTCTCACTTCGAGGACAAGCAG<br>GTCCAGAAGGCTTCCCTGGGTAT<br>CATGGGCACCGAGAAGTGCTGC<br>GACAACTGCCGTTCTCGCTAATA<br>ACTCGAGGCATGCGGTACCAAG<br>CTTGTCGAGAAGTACTAGAGGAT<br>CATAATCAGCCATACCACATTTGT<br>AGAGGTTTTACTTGCTTTAAAAAA<br>CCTCCCACACCTCCCCCTGAAC<br>CTGAAACATAAAATGAATGCAATT<br>GTTGTTGTTAACTTGTTTATTGCAG<br>CTTATAATGGTTACAAATAAAGCA<br>ATAGCATCACAAATTTCACAAATA<br>AAGCATTTTTTTCACTGCATTCTAG<br>TTGTGGTTTGTCCAAACTCATCAA<br>TGTATCTTATCATGTCTGGATCTGA<br>TCACTGCTTGAGCCTAGGAGATC<br>CGAACCAGATAAGTGAAATCTAG<br>TTCCAAACTATTTTGTCATTTTTAAT	

				TTTCGTATTAGCTTACGACGCTAC ACCCAGTTCCCATCTATTTTGTCA CTCTTCCCTAAATAATCCTTAAAA ACTCCATTTCCACCCCTCCCAGT TCCCAACTATTTTGTCCGCCCAC AGCGGGGCATTTTTCTTCCTGTTA TGTTTTTAATCAAACATCCTGCCA ACTCCATGTGACAAACCGTCATC TTCGGCTACTTTTTCTCTGTCACA GAATGAAAATTTTTCTGTCATCTCT TCGTTATTAATGTTTGTAATTGACT GAATATCAACGCTTATTTGCAGCC TGAATGGCGAATGG	
bioNMR	pFastBac1	HIS-FLG-THB-(WRN 500-942)	Same construct as for X-ray	Same construct as for X-ray	Same construct as for X-ray
Biochemistry	pFastBac1	HIS-FLG-THB-(WRN 500-942)	Same construct as for X-ray	Same construct as for X-ray	Same construct as for X-ray

Table SI 2. Biochemical and structural data for the WRN chemical matter.

Compound	Structure	MW (LE)	ATPase WRN IC50 (µM)	ATPase BLM IC50 (µM)	PDB code
Fragment 1	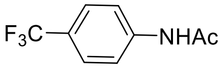	203.1 (NA)	>100	>100	9MJU
Fragment 2	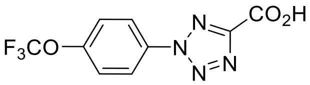	274.2 (NA)	>100	78*	9MJV
Fragment 3	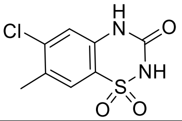	246.7 (NA)	>100	>100	9MJW
Fragment 4	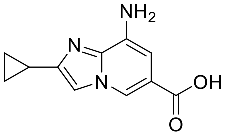	217.2 (NA)	>100	>100	9MJX
Compound 1	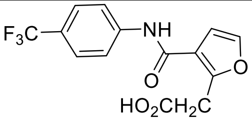	313.2 (0.23)	>100	73.4	9MJY
Compound 2	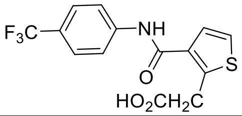	329.3 (0.26)	68.3 ± 2.0	3.7*	
Compound 3	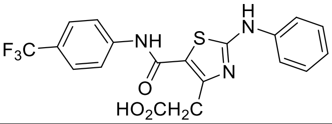	421.4 (0.25)	9.8 ± 4.0	22.8	9MJZ
Compound 4	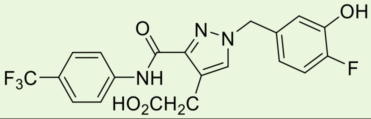	437.3 (0.28)	1.2 ± 0.7	>100	
Compound 5	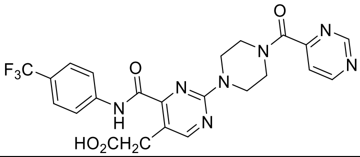	515.4 (0.18)	24.6 ± 20.7	28.3	9MK0
Compound 6	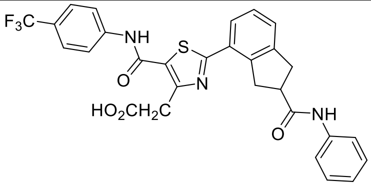	565.6 (0.19)	4.2 ± 1.1	4.5	9MK1
Compound 7	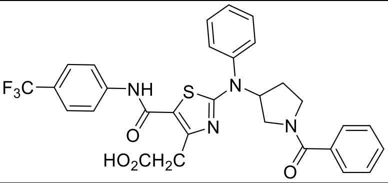	594.6 (0.14)	23.7 ± 0.6	14.8	

* Compounds with Hill slope ≥ 2.

Table SI 3. **Data collection and refinement statistics for all fragments and compounds.**

**PDB code**	**9MJS**	**9MJT**	**9MJU**	**9MJV**
**Compound name**	**AMP-PNP structure**	**Apo Form D**	**Fragment 1**	**Fragment 2**
**Data collection**				
**X-ray source**	X10SA (SLS)	X10SA (SLS)	X10SA (SLS)	X10SA (SLS)
**Wavelength** (Å)	1.0000	0.9999	1.0000	1.0000
**oscillation range total (°)**	220	220	220	220
**oscillation range per image (°)**	0.1	0.1	0.1	0.1
**Exposure time (s)**	0.01	0.01	0.01	0.01
**Detector**	Dectris EIGER2 Si 16M	Dectris EIGER2 Si 16M	Dectris EIGER2 Si 16M	Dectris EIGER2 Si 16M
Space group	*C*2	$P2_12_12_1$	$P2_12_12_1$	$P2_12_12_1$
Cell dimensions				
a, b, c (Å)	104.2, 93.7, 53.8	57.7, 58.2, 130.7	58.1, 58.5, 130.9	57.8, 58.4, 132.1
α, β, γ (°)	90.0, 104.4, 90.0	90.0, 90.0, 90.0	90.0, 90.0, 90.0	90.0, 90.0, 90.0
Average mosaicity (°)	0.17	0.20	0.07	0.36
Resolution (Å)	45.4-1.84 (1.87-1.84)*	65.3-1.73 (1.67-1.73)*	53.5-1.58 (1.61-1.60)*	53.4-2.58 (2.62-2.58)*
Unique reflections	41,646 (2,092)	46,791 (2,312)	61,984 (3,069)	14,021 (715)
Rpim	0.057	0.029	0.023	0.048
Rsym	0.080	0.078	0.062	0.128
Rmeas	0.08	0.08	0.07	0.08
Mean I/σI	8.2 (1.3)	13.3 (1.3)	15.1 (1.3)	11.2 (1.3)
Completeness (%)	96.3 (96.2)	100.0 (100.0)	100.0 (100.0)	95.5 (100.0)
CC(1/2) (%)	99.70 (52.80)	99.60 (59.00)	99.90 (57.60)	99.80 (53.50)
Multiplicity	2.8 (2.9)	8.2 (8.2)	8.2 (8.5)	8.1 (8.5)
**Refinement**				
Resolution (Å)	45.4-1.84	65.3-1.73	53.5-1.58	53.4-2.58
No. reflections (working /test)	39,208 / 2413	43,948 / 2843	58,159 / 3823	13,271 / 724
Rwork / Rfree	0.19 / 0.23	0.22 / 0.26	0.21 / 0.23	0.24 / 0.29
No. atoms				
Protein	3279	3427	3520	3,361
Ligand	31	--	14	19
Solvent	282	212	343	17
Other	1	10	18	5
B-factors				
Protein	36.2	42.3	33.1	72.78
Ligands	43.8	--	46.4	87.0
Solvent	42.2	46.4	43.0	60.6
Other	--	48.1	43.5	66.1
R.m.s. deviations				
Bond lengths (Å)	0.005	0.004	0.005	0.004
Bond angles (°)	1.380	1.33	1.36	1.38
Ramachandran Plot				
Favored [%]	98.51	97.88	97.88	93.30
Allowed [%]	1.49	1.89	1.88	6.46
Outliers [%]	0.00	0.24	0.24	0.24
MolProbity Clashscore	0.75	0.58	1.55	0.78
MolProbity Score	0.74	0.72	0.93	1.28

*Highest resolution shell (in Å) is shown in parentheses.

PDB code	9MJW	9MJX	9MJY	9MJZ
**Compound name**	**Fragment 3**	**Fragment 4**	**Compound 1**	**Compound 3**
**Data collection**				
**X-ray source**	X10SA (SLS)	X10SA (SLS)	X10SA (SLS)	I04 (DLS)
**Wavelength (Å)**	1.0000	1.0000	1.0000	0.9537
**oscillation range total**	220	220	220	220
**oscillation range per image**	0.1	0.1	0.1	0.1
**Exposure time (s)**	0.01	0.01	0.01	0.015
**Detector**	Dectris EIGER2 Si 16M	Dectris EIGER2 Si 16M	Dectris EIGER2 Si 16M	Dectris EIGER2 XE 16M
Space group	$P2_12_12_1$	$P2_12_12_1$	$P2_12_12_1$	$P2_12_12_1$
Cell dimensions				
a, b, c (Å)	55.6, 62.3, 135.1	57.4, 58.1, 130.8	57.7, 57.9, 130.3	58.1, 58.1, 131.6
α, β, γ (°)	90.0, 90.0, 90.0	90.0, 90.0, 90.0	90.0, 90.0, 90.0	90.0, 90.0, 90.0
Average mosaicity (°)	0.95	0.11	0.19	0.05
Resolution (Å)	51.5-2.00 (2.03-2.00)*	43.5-1.73 (1.76-1.73)*	52.8-1.63 (1.66-1.63)*	53.2-1.70 (1.73-1.70)*
Unique reflections	21,778 (1,280)	46,408 (2,276)	54,424 (2,761)	49,994 (2,456)
Rpim	0.051	0.029	0.020	0.014
Rsym	0.091	0.072	0.055	0.037
Rmeas	0.11	0.08	0.06	0.04
Mean I/σI	9.8 (1.3)	13.1 (1.3)	16.9 (1.4)	22.6 (8.2)
Completeness (%)	66.8 (79.8)	100.0 (100.0)	98.4 (100.0)	100.0 (100.0)
CC(1/2) (%)	99.50 (51.10)	99.90 (49.50)	99.90 (66.20)	99.60 (69.90)
Multiplicity	3.8 (3.8)	7.1 (7.4)	8.1 (8.3)	8.3 (8.2)
**Refinement**				
Resolution (Å)	51.5-2.00	43.5-1.73	52.8-1.63	34.9-1.70
No. reflections(working /test)	21,252 / 504	45,762 / 641	53,674 / 744	41,609 / 893
No. reflections	21,252	45,762	53,674	41,609
Rwork / Rfree	0.22/0.26	0.20/0.23	0.19/0.22	0.21 / 0.26
No. atoms				
Protein	3,295	3,445	3,501	3,423
Ligand	15	27	44	29
Solvent	138	229	259	194
Other	1	34	30	43
B-factors				
Protein	38.6	42.7	37.3	45.2
Ligands	35.0	44.8	41.1	35.2
Solvent	35.3	47.2	43.0	50.2
Other	32.4	62.0	52.5	64.2
R.m.s. deviations				
Bond lengths (Å)	0.007	0.006	0.004	0.006
Bond angles (°)	1.55	1.41	1.17	1.43
Ramachandran Plot				
Favored [%]	95.57	98.36	97.22	98.33
Allowed [%]	4.19	1.17	2.55	1.43
Outliers [%]	0.25	0.47	0.23	0.24
MolProbity Clashscore	1.66	1.71	1.55	1.14
MolProbity Score	1.48	0.93	1.04	0.82

*Highest resolution shell (in Å) is shown in parentheses.

PDB code	9MK0	9MK1
**Compound name**	**Compound 5**	**Compound 6**
**Data collection**		
**X-ray source**	X10SA (SLS)	ID23-1 (ESRF)
**Wavelength (Å)**	0.9999	0.8856
**oscillation range total (°)**	220	206.8
**oscillation range per image (°)**	0.1	0.094
**Exposure time (s)**	0.01	0.012
**Detector**	Dectris EIGER2 Si 16M	Dectris EIGER2 CdTe 16M
Space group	*I*222	$P2_12_12_1$
Cell dimensions		
a, b, c (Å)	80.6, 96.1, 149.1	57.8, 57.8, 133.5
α, β, γ (°)	90.0, 90.0, 90.0	90.0, 90.0, 90.0
Average mosaicity (°)	0.05	0.50
Resolution (Å)	47.6-1.89 (1.92-1.89)*	53.0-2.12 (2.15-2.12)*
Unique reflections	46,576 (2,304)	26,085 (1,251)
Rpim	0.035	0.037
Rsym	0.097	0.094
Rmeas	0.103	0.101
Mean I/σI	10.2 (1.4)	12.1 (1.4)
Completeness (%)	100.0 (100.0)	99.4 (96.0)
CC(1/2) (%)	99.70 (64.70)	99.70 (56.40)
Multiplicity	8.5 (8.7)	7.3 (6.6)
**Refinement**		
Resolution (Å)	47.6-1.89	53.0-2.12
No. reflections(working /test)	44,158 / 2,388	24,831 / 1252
Rwork / Rfree	0.19 / 0.23	0.23 / 0.27
No. atoms		
Protein	3,421	3,428
Ligand	45	40
Solvent	233	79
Other	57	9
B-factors		
Protein	51.8	52.5
Ligands	43.6	42.1
Solvent	56.8	48.8
Other	60.6	51.8
R.m.s. deviations		
Bond lengths (Å)	0.004	0.003
Bond angles (°)	1.36	1.35
Ramachandran Plot		
Favored [%]	98.81	97.39
Allowed [%]	0.95	2.61
Outliers [%]	0.24	0.00
MolProbity Clashscore	1.98	0.72
MolProbity Score	0.98	0.85

*Highest resolution shell (in Å) is shown in parentheses.

Chemistry

General procedures and reaction conditions

2-(3-((4-(trifluoromethyl)phenyl)carbamoyl)furan-2-yl)acetic acid (1) and 2-(3-((4-(trifluoromethyl)phenyl)carbamoyl)thiophen-2-yl)acetic acid (2)

Scheme 1. Synthesis of compound 1 and 2.

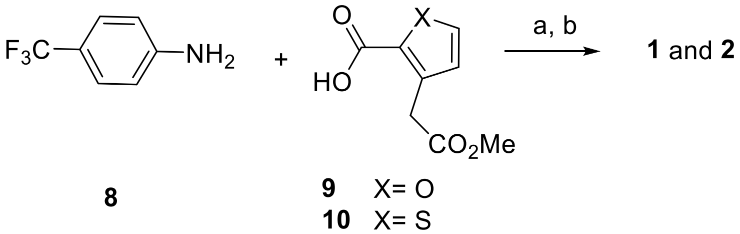

Reagents and conditions: (a) EDCI, pyridine, rt; (b) LiOH, MeOH, H_2O, 45 ºC.

General procedure for the preparation of compounds exemplified as 1 and 2.

To a mixture of acid exemplified as **9** (1.2 equiv.) in pyridine was added EDCI (2 equiv.) followed by a solution of aniline **8** (1 equiv.) in pyridine. The resulting mixture was stirred at rt for 12 h, then the reaction mixture was evaporated to dryness. To a solution of this crude product in a mixture of THF and water was added LiOH and heated at 45 ºC for 12 h. Then the reaction mixture was cooled to rt and acidified to pH 2-3 using 1 N aqueous solution of HCl, evaporated, and purified by reverse phase HPLC to afford the compounds exemplified as **1** and **2**. ^{1}H NMR of compound **1** (600 MHz, DMSO) δ 12.58 (bs, 1H), 10.11 (s, 1H), 7.96 (d, *J* = 8.5 Hz, 2H), 7.76 – 7.62 (m, 3H), 7.17 (d, *J* = 2.1 Hz, 1H), 4.06 (s, 2H). ^{13}C NMR of compound **1** (151 MHz, DMSO) δ 169.93, 161.64, 154.33, 142.38 (q, *J* = 1.4 Hz), 141.82, 125.77 (q, *J* = 3.8 Hz), 124.29 (q, *J* = 271.2 Hz), 123.43 (q, *J* = 32.1 Hz), 119.99, 117.13, 109.16, 33.32. ^{1}H NMR of compound **2** (600 MHz, DMSO) δ 12.5 (bs, 1H), 10.33 (s, 1H), 7.94 (d, *J* = 8.6 Hz, 2H), 7.70 (d, *J* = 8.6 Hz, 2H), 7.58 (d, *J* = 5.4 Hz, 1H), 7.52 (d, *J* = 5.4 Hz, 1H), 4.14 (s, 2H). ^{13}C NMR of compound **2** (151 MHz, DMSO) δ 171.12, 162.51, 142.57, 142.02, 132.78, 127.07, 125.77 (q, *J* = 3.9 Hz), 124.30 (q, *J* = 271.2 Hz), 123.84, 123.37 (q, *J* = 32.0 Hz), 119.90, 33.72.

HRMS (ESI): m/z calcd for **1** (C14H10F3NO4 + H)$^+$, 314.0635; found, 314.0638. HRMS (ESI): m/z calcd for **2** (C14H10F3NO3S + H)$^+$, 330.0406; found, 330.0408.

2-(2-(phenylamino)-5-((4-(trifluoromethyl)phenyl)carbamoyl)thiazol-4-yl)acetic acid (3)

Scheme 2. Synthesis of compound 3.

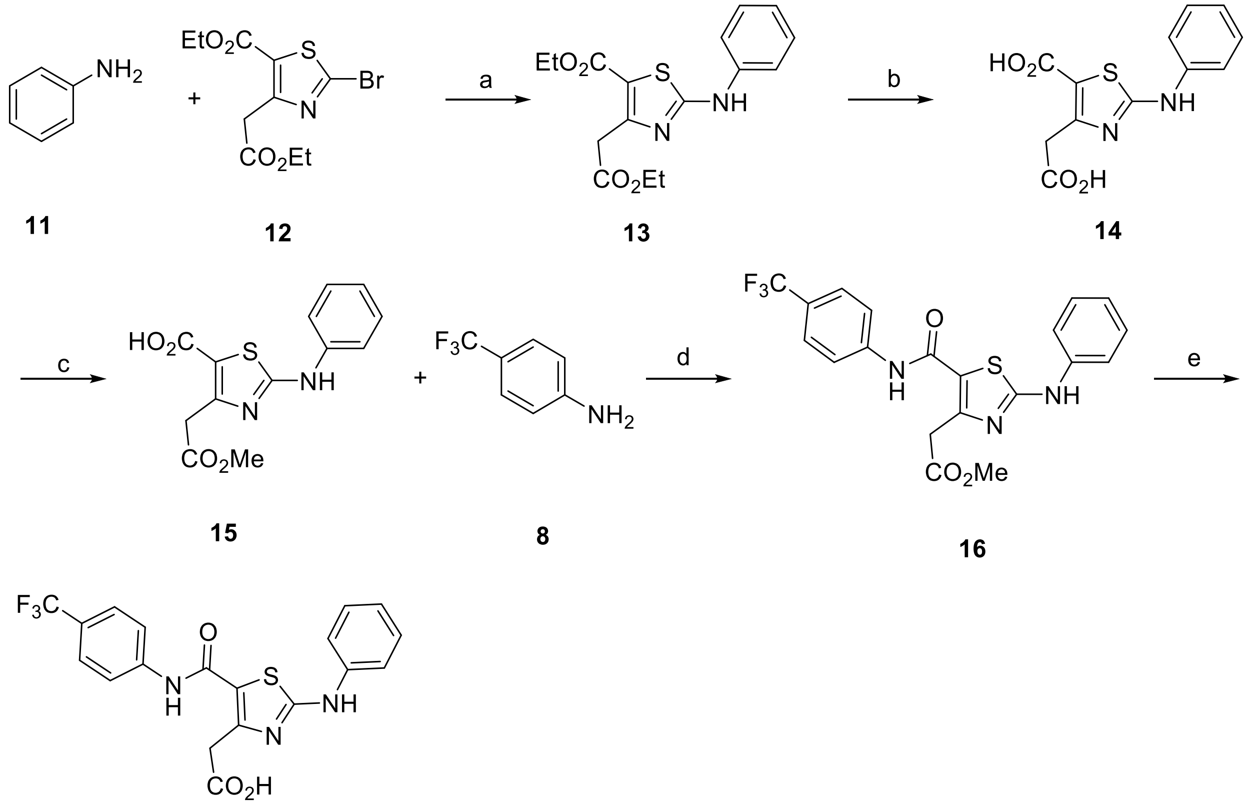

Reagents and conditions: (a) $Pd_2(dba)_3$, Xantphos,Cs_2CO_3, dioxane, yield 15.4%; (b) LiOH, MeOH, Water, yield 40.6%; (c) HCl, MeOH, yield 57.1%; (d) Py, $POCl_3$, DCM, yield 23.5%; (e) Me_3SnOH, DCE, yield 31%.

Palladium catalyzed C-N coupling between **11** and **12** followed by hydrolysis of the ethyl esters led to formation of bis carboxylic acid **14**. Selective esterification of less hindered primary acid of **14** followed by amide coupling of the remaining carboxylic acid with **8** afforded **16**. Finally, trimethyltin hydroxide mediated hydrolysis of methyl ester of **16** led to formation of **3**.

Experimental procedure for synthesis of compound 3.
Synthesis of ethyl 4-(2-ethoxy-2-oxoethyl)-2-(phenylamino)thiazole-5-carboxylate (**13**)

To a solution of **12** (1.99 g, 6.21 mmol) in dioxane (30 mL) was added aniline **11** (0.867 g, 9.31 mmol), Cs_2CO_3 (6.07 g, 18.62 mmol), Xantphos (0.718 g, 1.24 mmol) and $Pd_2(dba)_3$ (0.568 g, 0.621 mmol) under N_2, and the mixture was stirred at 100 °C for 16 h. The reaction mixture was treated with water (50 mL) and extracted with EtOAc (3 × 30 mL). The combined organic layers were washed with brine (30 mL), dried over anhydrous Na_2SO_4, filtered, and concentrated in vacuo. The residue was purified by reverse phase HPLC to afford ethyl 4-(2-ethoxy-2-oxoethyl)-

2-(phenylamino)thiazole-5-carboxylate (**13**) (0.320 g, 0.96 mmol, 15.4% yield) as a yellow oil. MS (ESI): *m/z* $(M+H)^+$ 335.0.

Synthesis of 4-(carboxymethyl)-2-(phenylamino)thiazole-5-carboxylic acid (**14**)

To a mixture ethyl 4-(2-ethoxy-2-oxoethyl)-2-(phenylamino)thiazole-5-carboxylate (**13**) (0.40 g, 1.12 mmol) in a mixture of MeOH (5 mL) and water (0.25 mL) was added LiOH (0.086 g, 3.59 mmol) and stirred at 25 °C for 3 h. The reaction mixture was neutralized and purified by reverse phase HPLC to give 4-(carboxymethyl)-2-(phenylamino)thiazole-5-carboxylic acid (**14**) (0.15 g, 0.46 mmol, 40.6% yield) as a yellow oil. MS (ESI): *m/z* $(M+H)^+$ 278.9.

Synthesis of 4-(2-methoxy-2-oxoethyl)-2-(phenylamino)thiazole-5-carboxylic acid (**15**)

A mixture of 4-(carboxymethyl)-2-(phenylamino)thiazole-5-carboxylic acid (**14**) (0.20 g, 0.719 mmol) and conc. HCl (0.06 mL, 0.72 mmol) in MeOH (6 mL) was stirred at 20 °C for 8 h. The mixture was treated with 20 mL of water and extracted with EtOAc (15 mL x 3). The combined organic layers were washed with brine (10 mL x 2), dried over anhydrous Na_2SO_4, filtered, and concentrated under reduced pressure to give crude 4-(2-methoxy-2-oxoethyl)-2-(phenylamino)thiazole-5-carboxylic acid (**15**) (0.12 g, 0.41 mmol, 57.1% yield) as a yellow solid, which was used in the next step directly without further purification. MS (ESI): *m/z* $(M+H)^+$ 293.0.
Synthesis of methyl 2-(2-(phenylamino)-5-((4-(trifluoromethyl)phenyl)carbamoyl)thiazol-4-yl)acetate (**16**)

To a solution of 4-(2-methoxy-2-oxoethyl)-2-(phenylamino)thiazole-5-carboxylic acid (**15**) (0.095 g, 0.33 mmol) in DCM (1.5 mL) was added 4-(trifluoromethyl)aniline (**8**) (0.052 g, 0.33 mmol), Pyridine (0.04 mL, 0.49 mmol) and $POCl_3$ (0.036 mL, 0.390 mmol) at 0 °C, and stirred at 0 °C for 2 h. The solvent was removed in vacuum. The residue was treated with water (10 mL) and extracted with EtOAc (3 × 10 mL). The combined organic layers were washed with brine (10 mL), dried over anhydrous Na_2SO_4, filtered, and concentrated in vacuo. The residue was purified by reversed phase HPLC to give methyl 2-(2-(phenylamino)-5-((4-(trifluoromethyl)phenyl)carbamoyl)thiazol-4-yl)acetate (**16**) (0.035 g, 0.08 mmol, 23.50% yield) as white solid. MS (ESI): *m/z* $(M+H)^+$ 436.0. ^{1}H NMR ($CDCl_3$, 400 MHz) δ 10.02 (s, 1H), 7.77 (d, *J* = 8.5 Hz, 2H), 7.61 (d, *J* = 8.6 Hz, 2H), 7.40-7.47 (m, 2H), 7.34 (d, *J* = 7.6 Hz, 2H), 7.18-7.25 (m, 1H), 3.99 (s, 2H), 3.90 (s, 3H).

Synthesis of 2-(2-(phenylamino)-5-((4-(trifluoromethyl)phenyl)carbamoyl)thiazol-4-yl)acetic acid (**3**)

To a solution of methyl 2-(2-(phenylamino)-5-((4-(trifluoromethyl)phenyl)carbamoyl)thiazol-4-yl)acetate (**16**) (0.035 g, 0.080 mmol) in DCE (1 mL) was added Me_3SnOH (0.044g, 0.24 mmol) and stirred at 25 °C for 16 h. The reaction mixture was purified by reverse phase HPLC to give 2-(2-(phenylamino)-5-((4-(trifluoromethyl)phenyl)carbamoyl)thiazol-4-yl)acetic acid (**3**) (0.011 g, 0.025 mmol, 31.0% yield) as a yellow oil. ^{1}H NMR (600 MHz, DMSO) δ 12.44 (bs, 1H), 10.69 (s, 1H), 10.15 (s, 1H), 7.87 (d, *J* = 8.5 Hz, 2H), 7.69 (d, *J* = 8.6 Hz, 2H), 7.63 – 7.56 (m, 2H),

7.41 – 7.33 (m, 2H), 7.05 (tt, *J* = 7.3, 1.1 Hz, 1H), 3.98 (s, 2H). ^{13}C NMR (151 MHz, DMSO) δ 171.11, 163.47, 160.23, 152.40, 142.48, 140.14, 129.09, 125.73 (q, *J* = 3.9 Hz), 124.28 (q, *J* = 271.8 Hz), 122.44, 120.17, 117.79, 113.97, 36.81. HRMS (ESI): m/z calcd for **3** (C19H14F3N3O3S + H)$^+$, 422.0781; found, 422.0785.

2-(1-(4-fluoro-3-hydroxybenzyl)-3-((4-(trifluoromethyl)phenyl)carbamoyl)-1H-pyrazol-4-yl)acetic acid (4)

Scheme 3. Synthesis of compound 4.

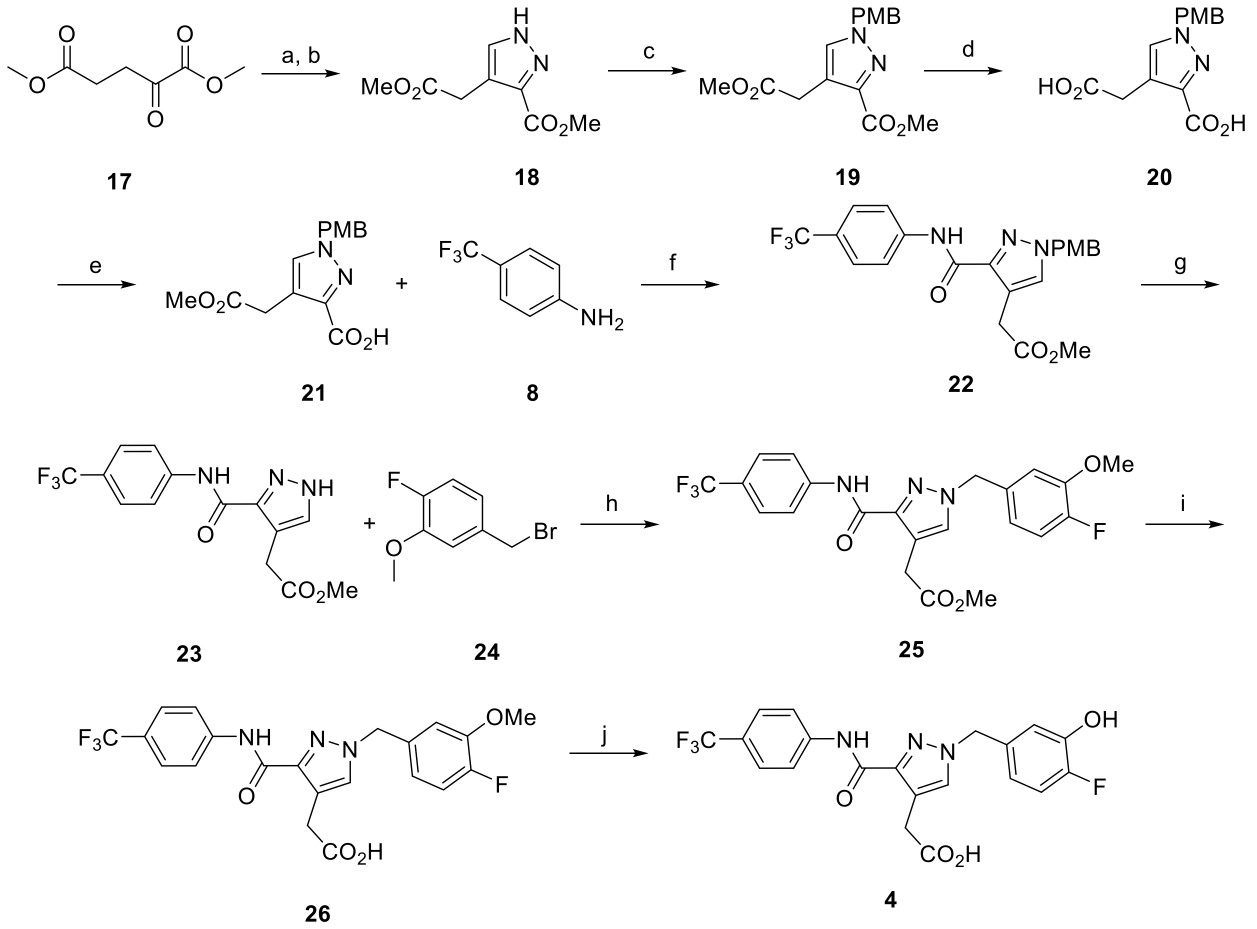

Reagents and conditions: (a) DMF-DMA, toluene, 70 °C; b) 2) N_2H_4 H2O, HOAc, 20 °C, 61.5%; (c) PMB-Cl, K_2CO_3, DMF, 25 °C, 46%; (d) LiOH H_2O, THF/H_2O, 25 °C, 94%; (e) HCl, MeOH, ~100%; (f) TCFH, NMI, DMF, 25 °C, 47.3%; (g) CAN, MeCN/H_2O, 77%; (h) K_2CO_3, DMF, 25 °C, 83%; (i) Me_3SnOH, DCE, 41.5%; (j) BBr3, DCM, 25.7%.

Condensation of **17** with DMF-DMA followed by treatment of the resulting enone with hydrazine afforded pyrazole **18**. PMB protected compound **19** was progressed to **22** following the protocol described in scheme 2. Removal of PMB and installation methoxy fluoro benzyl moiety

afforded **25**. Finally, hydrolysis of methyl ester followed by treatment of resulting compound with BBr_3 provided final compound **4**.

Experimental procedure for synthesis of compound 4.

Synthesis of methyl 4-(2-methoxy-2-oxoethyl)-1H-pyrazole-3-carboxylate (**18**)

To a solution of **17** (9.99 g, 57.4 mmol) in toluene (100 mL) was added DMF-DMA (10.26 g, 86 mmol) at 20 °C. The mixture was stirred at 70 °C for 16 h. The reaction mixture was concentrated to give crude dimethyl (Z)-3-((dimethylamino) methylene)-2-oxopentanedioate (13.16 g, ~100% yield) as a yellow oil, which was used in the next step without further purification. To a solution of dimethyl (Z)-3-((dimethylamino)methylene)-2-oxopentanedioate (13.16 g, 57.4 mmol) in HOAc (100 mL) was added hydrazine hydrate (11.50 g, 230 mmol) at 20 °C. The mixture was stirred at 20 °C for 16 h. The reaction mixture was concentrated to remove most of HOAc. The residue was treated with EtOAc (300 mL), washed successively with saturated aqueous solution of $NaHCO_3$ (100 mL) and brine (50 mL), dried over Na_2SO_4, filtered, and concentrated to give crude **18** (6.99 g, 35.5 mmol, 61.5% yield) as a yellow solid, which was used in the next step without further purification. ^1H NMR ($CDCl_3$, 400 MHz) δ 7.76 (s, 1H), 3.95 (s, 3H), 3.86 (s, 2H), 3.73 (s, 3H).

Synthesis of methyl 4-(2-methoxy-2-oxoethyl)-1-(4-methoxybenzyl)-1H-pyrazole-3-carboxylate (**19**)

To a solution of **18** (2.99 g, 15.14 mmol) in DMF (30 mL) was added PMB-Cl (2.61 g, 16.65 mmol) and K_2CO_3 (6.28 g, 45.4 mmol) at 25 °C. The mixture was stirred at 25 °C for 16 h. The reaction mixture was diluted with water (250 mL) and extracted with EtOAc (150 mL x 4). The combined organic layers were washed successively with water (150 mL) and brine (150 mL), dried over Na_2SO_4, filtered, and concentrated to give crude product, which was purified by silica gel chromatography using 0-10% EtOAc in hexanes to give **19** (2.33 g, 6.97 mmol, 46% yield) as the major product.**:** *m/z* $(M+H)^+$ 318.9.

Synthesis of 4-(carboxymethyl)-1-(4-methoxybenzyl)-1H-pyrazole-3-carboxylic acid (**20**)

To a solution of **19** (2.34 g, 7.34 mmol) in THF (69 mL) and water (23 mL) was added lithium hydroxide hydrate (1.54 g, 36.7 mmol) at 25 °C. Then the reaction mixture was stirred at 25 °C for 16 h. The reaction mixture was concentrated to removed most of THF. The residue was diluted with water (200 mL) and pH of the resulting solution was adjusted to ~ 4 with 1 M HCl. The aqueous layer was extracted with EtOAc (40 mL x 4). The combined organic layers were washed with brine (40 mL), dried over Na_2SO_4, filtered, and concentrated to give **20** (2.13 g, 6.89 mmol, 94% yield) as a white solid, which was used in the next step without further purification. MS (ESI): *m/z* $(M+H)^+$ 290.9.

Synthesis of 4-(2-methoxy-2-oxoethyl)-1-(4-methoxybenzyl)-1H-pyrazole-3-carboxylic acid (**21**)

To a solution of di acid **20** (2.13 g, 7.33 mmol) in MeOH (50 mL) was added conc. HCl (0.60 mL, 7.33 mmol) at 25 °C. Then the reaction mixture was stirred at 25 °C for 16 h. The reaction mixture was concentrated to remove MeOH. The residue was treated with EtOAc (100 mL), washed with water (50 mL) and then with brine (20 mL), dried over Na_2SO_4, filtered, and concentrated to give mono acid nomo ester **21** (2.23 g, 7.33 mmol, ~100% yield) as a brown solid, which was used in the next step without further purification. MS (ESI): *m/z* $(M+H)^+$ 304.8.

Synthesis of methyl 2-(1-(4-methoxybenzyl)-3-((4-(trifluoromethyl)phenyl)carbamoyl)-1H-pyrazol-4-yl)acetate (**22**)

To a solution of **21** (2.23 g, 7.33 mmol) in DMF (50 mL) was added 4-(trifluoromethyl)aniline **8** (1.54 g, 9.54 mmol), TCFH (3.09 g, 11.00 mmol) and NMI (1.81 g, 22.00 mmol) at 25 °C. Then the reaction mixture was stirred at 25 °C for 16 h. The reaction mixture was purified by reverse phase HPLC) to **22** (1.59 g, 3.47 mmol, 47.3% yield) as a brown solid. MS (ESI): *m/z* $(M+H)^+$ 448.0.

Synthesis of methyl 2-(3-((4-(trifluoromethyl)phenyl)carbamoyl)-1H-pyrazol-4-yl)acetate(**23**)

To a solution of **22** (1.58 g, 3.54 mmol) in MeCN (19.5 mL) and water (19.5 mL) was added CAN (5.82 g, 10.62 mmol) at 25 °C. Then the reaction mixture was stirred at 25 °C for 2 h. The reaction mixture was diluted with water (50 mL) and extracted with EtOAc (30 mL x 4). The combined organic layers were washed with brine (30 mL), dried over Na_2SO_4, filtered, and concentrated to give crude product, which was purified by silica gel chromatography using 0–22% EtOAc in hexanes to give **23** (0.95 g, 2.71 mmol, 77% yield) as a white solid. MS (ESI): *m/z* $(M+H)^+$ 327.9.

Synthesis of methyl 2-(1-(4-fluoro-3-methoxybenzyl)-3-((4-(trifluoromethyl)phenyl)carbamoyl)-1H-pyrazol-4-yl)acetate (**25**)

To a solution of **23** (0.15 g, 0.49 mmol) in DMF (2.5 mL) was added **24** (0.11 g, 0.50 mmol) and K_2CO_3 (0.19 g, 1.38 mmol) at 25 °C. The mixture was stirred at 25 °C for 36 h. The reaction mixture was diluted with water (25 mL) and extracted with EtOAc (15 mL x 4). The combined organic layers were washed with water (20 mL) and then with brine (20 mL), dried over Na_2SO_4, filtered, and concentrated to give crude product, which was purified by silica gel chromatography using 0-15% EtOAc in hexanes to give **25** (0.203 g, 0.38 mmol, 83% yield) a white solid. ^{1}H NMR ($CDCl_3$, 400 MHz) δ 8.87 (s, 1H), 7.79 (d, *J* = 8.5 Hz, 2H), 7.60 (d, *J* = 8.6 Hz, 2H), 7.51 (s, 1H), 7.13 - 7.04 (m, 1H), 6.86 - 6.75 (m, 2H), 5.28 (s, 2H), 3.96 (s, 2H), 3.88 (s, 3H), 3.73 (s, 3H). MS (ESI): *m/z* $(M+H)^+$ 466.0.

Synthesis of 2-(1-(4-fluoro-3-methoxybenzyl)-3-((4-(trifluoromethyl)phenyl)carbamoyl)-1H-pyrazol-4-yl)acetic acid (**26**)

To a solution of **25** (0.040 g, 0.09 mmol) in DCE (1.2 mL) was added Me_3SnOH (0.047g, 0.258 mmol) at 25 °C. Then the reaction mixture was stirred at 25 °C for 16 h, and at 45 °C for

another 8 h. The reaction mixture was concentrated to give crude product, which was purified by reverse to give **26** (0.018 g, 0.036 mmol, 41.5% yield) as a white solid. MS (ESI): *m/z* $(M+H)^+$ 452.1. ^{1}H NMR ($CDCl_3$, 400 MHz) δ 8.95 (s, 1H), 7.82 (d, *J* = 8.6 Hz, 2H), 7.65 (d, *J* = 8.6 Hz, 2H), 7.47 (s, 1H), 7.10 (dd, *J* = 10.9, 8.3 Hz, 1H), 6.76-6.89 (m, 2H), 5.28 (s, 2H), 3.89 (s, 3H), 3.82 (s, 2H).

2-(1-(4-fluoro-3-hydroxybenzyl)-3-((4-(trifluoromethyl)phenyl)carbamoyl)-1H-pyrazol-4-yl)acetic acid (**4**)

To a solution of **26** (0.099 g, 0.22 mmol) in DCM (2 mL) was added BBr_3 (0.67 mL, 0.67 mmol) dropwise at 0 °C under N_2. Then the reaction mixture was stirred at 25 °C for 2 h. The reaction mixture was diluted with DCM (20 mL), washed with ice water (15 mL) and then with brine (15 mL), dried over Na_2SO_4, filtered, and concentrated to give crude product, which was purified by reverse phase HPLC to give **4** (0.027 g, 0.057 mmol, 25.7% yield) as a white solid. ^{1}H NMR (600 MHz, DMSO) δ 12.18 (s, 1H), 10.40 (s, 1H), 9.95 (s, 1H), 8.03 (d, *J* = 8.3 Hz, 2H), 7.85 (s, 1H), 7.67 (d, *J* = 8.3 Hz, 2H), 7.13 (dd, *J* = 11.3, 8.3 Hz, 1H), 6.85 (dd, *J* = 8.5, 2.2 Hz, 1H), 6.74 (ddd, *J* = 8.4, 4.2, 2.2 Hz, 1H), 5.34 (s, 2H), 3.74 (s, 2H). ^{13}C NMR (151 MHz, DMSO) δ 172.16, 161.18, 150.41 (d, J = 241.0 Hz), 144.84 (d, J = 12.4 Hz), 142.73, 142.32 (q, J = 1.5 Hz), 133.16 (d, J = 3.4 Hz), 132.53, 125.66 (q, J = 3.8 Hz), 124.32 (q, J = 271.5 Hz), 123.19 (q, J = 31.8 Hz), 119.93, 118.48 (d, J = 6.8 Hz), 116.84, 116.77 (d, J = 3.2 Hz), 116.16 (d, J = 18.4 Hz), 54.52, 29.45. HRMS (ESI): m/z calcd for **4** $(C20H15F4N3O4 + H)^+$, 438.1072; found, 438.1076.

2-(2-(4-(pyrimidine-4-carbonyl)piperazin-1-yl)-4-((4-(trifluoromethyl)phenyl)carbamoyl)pyrimidin-5-yl)acetic acid (5)

Scheme 4. Synthesis of compound 5.

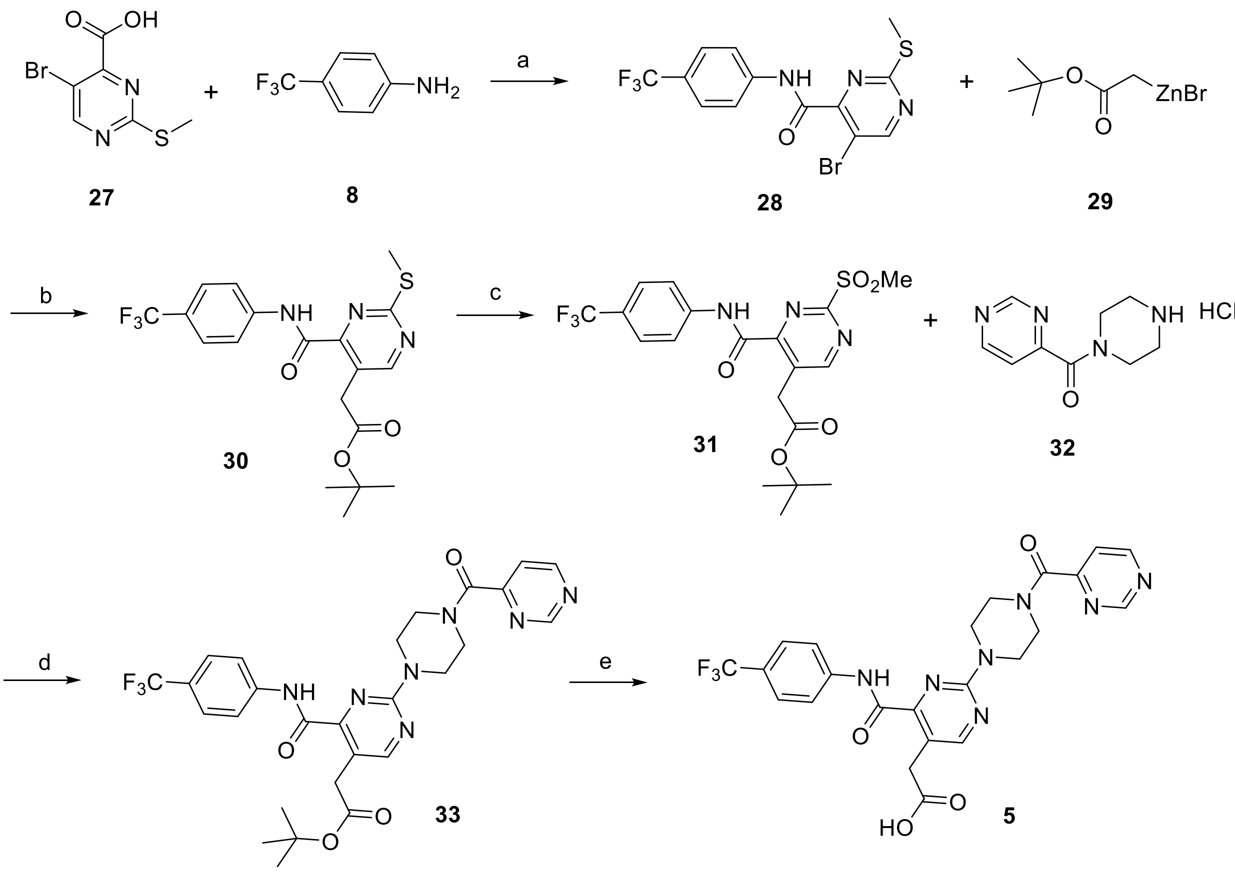

Reagents and conditions: (a) 1-Chloro-N,N,2-trimethylpropenylamine, DIEA, DCM, yield 67.1%; (b) 2nd Generation P(t-Bu)3 Pd pre-catalyst, potassium acetate, yield 15.9%; (c) mCPA, DCM, yield 81%; (d) DIEA, NMP, yield 56.5%; (d) TFA, DCM, yield 84%.

Amide coupling between **27** and aniline **8** mediated by Ghosez reagent followed by Negishi reaction of the resulting amide **28** with **29** led to formation of **30**. Sulfide moiety of **30** was oxidized to sulfone **31** for the S_NAr reaction with amine **32**. Finally, removal of *tert*-butyl ester protecting carboxylic acid of **33** led to the formation of **5**.

Experimental procedure for synthesis of compound 5.

Synthesis of 5-bromo-2-(methylthio)-N-(4-(trifluoromethyl)phenyl)pyrimidine-4-carboxamide (**28**)

To a solution of **27** (2.47 g, 10 mmol) in DCM (25 ml) was added 1-chloro-N,N,2-trimethylprop-1-en-1-amine (1.99 mL, 15 mmol) at rt and stirred for 15 min. The resulting clear light brown solution was cool to 0 °C and was added a solution of 4-(trifluoromethyl)aniline **8** (1.94 g, 12 mmol) in DCM (2 mL) followed N-ethyl-N-isopropylpropan-2-amine (5.24 mL, 30.1 mmol). Ice bath was removed, and the resulting mixture was stirred at rt for 10 min. The reaction mixture was diluted with DCM and washed with an aqueous solution of 1 N HCl (50 mL). The organic layer was dried with Na_2SO_4, filtered, concentrated and the crude was purified by silica

gel column chromatography using 10-20% EtOAc in hexanes to afford **28** (2.78 g, 6.73 mmol, 67.1% yield) .

Synthesis of tert-butyl 2-(2-(methylthio)-4-((4-(trifluoromethyl)phenyl)carbamoyl)pyrimidin-5-yl)acetate (**30**)

A mixture of **28** (0.75 g, 1.91 mmol), 2nd Generation P(t-Bu)$_3$ Pd pre-catalyst (0.196 g, 0.38 mmol), and potassium acetate (0.563 g, 5.74 mmol) was degassed and then to this mixture was added a solution of ((2-(tert-butoxy)-2-oxoethyl)zinc(II) bromide **29** (13.3 mL, 6.69 mmol) in THF. The resulting mixture was mixture was degassed and heated at 100 °C in a microwave reactor for 1 h. The reaction mixture was cooled to rt, carefully quenched with an aqueous solution of HCl (0.5 M) and concentrated to remove THF. Then the resulting mixture was transferred to a separatory funnel and extracted with EtOAc. The organic layer was washed successively with water and brine, dried with Na_2SO_4, filtered, concentrated and the crude product was purified by silica gel column chromatography using 20-50% EtOAc in hexanes to afford **30** (0.136 g, 0.30 mmol, 15.9% yield) as solid.

Synthesis of tert-butyl 2-(2-(methylsulfonyl)-4-((4-(trifluoromethyl)phenyl)carbamoyl)pyrimidin-5-yl)acetate (**31**)

To a solution of **30** (0.136 g, 0.32 mmol) in DCM (1 mL) was added 3-chloroperoxybenzoic acid (0.165 g, 0.96 mmol) at rt and stirred at rt for 1 h. The reaction mixture was quenched with a saturated aqueous solution of $NaHCO_3$ (1 mL), extracted with DCM (2 x 20 mL). The organic layer was dried with Na_2SO_4, filtered and concentrated and the crude **31** (0.132 g, 0.26 mmol, 81% yield) was taken to the next step without purification.

Synthesis of tert-butyl 2-(2-(4-(pyrimidine-4-carbonyl)piperazin-1-yl)-4-((4-(trifluoromethyl)phenyl)carbamoyl)pyrimidin-5-yl)acetate (**33**)

A mixture of **31** (0.050 g, 0.11 mmol), piperazin-1-yl(pyrimidin-4-yl)methanone hydrochloride **32** (0.027 g, 0.12 mmol) and *N*-ethyl-N-isopropylpropan-2-amine (0.076 mL, 0.435 mmol) in NMP (1 mL) was heated under microwave irradiation at 150 °C for 1 h. The reaction mixture was diluted with water and extracted with extracted with EtOAc (2 x 15 mL). The organic layer was separated, dried with Na_2SO_4, filter, concentrated and the crude product was purified by silica gel column chromatography using 30-60% EtOAc in hexanes to afford **33** (0.037 g, 0.06 mmol, 56.5% yield).

Synthesis of 2-(2-(4-(pyrimidine-4-carbonyl)piperazin-1-yl)-4-((4-(trifluoromethyl)phenyl)carbamoyl)pyrimidin-5-yl)acetic acid (**5**)

To a solution of **33** (0.025 g, 0.04 mmol) in DCM (1 mL) was added TFA (0.33 mL) and stirred for 30 min . The reaction mixture was concentrated, and the crude product was purified by reverse phase HPLC to afford **5** (0.019 g, 84% yield). ^{1}H NMR (600 MHz, DMSO) δ 10.72 (bs, 1H), 9.29

(d, *J* = 1.5 Hz, 1H), 9.01 (d, *J* = 5.1 Hz, 1H), 8.49 (s, 1H), 7.99 (d, *J* = 8.4 Hz, 2H), 7.77 – 7.71 (m, 3H), 4.01– 3.98 (m, 2H), 3.92 – 3.85 (m, 2H), 3.84 – 3.76 (m, 4H), 3.54 – 3.49 (m, 2H). ^{13}C NMR (151 MHz, DMSO) δ 172.20, 164.90, 163.96, 162.62, 160.68, 159.61, 158.74, 157.83, 155.76, 141.58, 125.83 (q, *J* = 3.8 Hz), 124.21 (q, *J* = 271.2 Hz), 124.02 (q, *J* = 32.1 Hz), 120.30, 119.98, 115.68, 46.0, 43.69, 43.04, 41.29, 34.16. HRMS (ESI): m/z calcd for **5** (C23H20F3N7O4 + H)$^+$, 516.1602; found, 516.1606..

2-(2-(2-(phenylcarbamoyl)-2,3-dihydro-1H-inden-4-yl)-5-((4-(trifluoromethyl)phenyl)carbamoyl)thiazol-4-yl)acetic acid (6)

Scheme 5. Synthesis of compound 6.

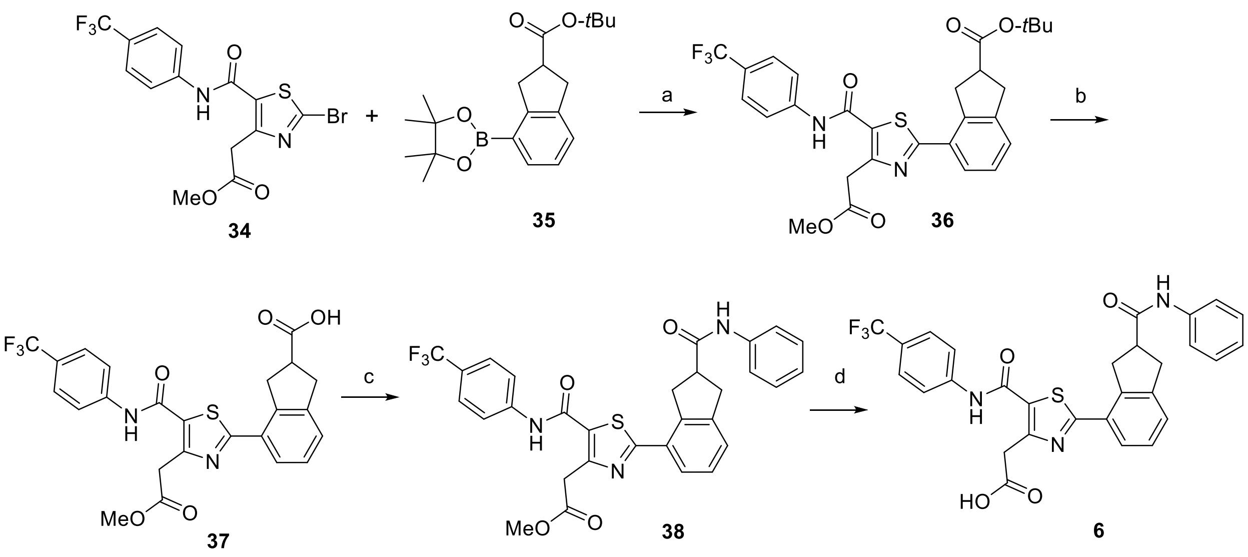

Reagents and conditions: (a) Pd(dppf)Cl_2·CH_2Cl_2, Cs_2CO_3, 1,4-dioxane, 60 ºC, 73.1%; (b) 4 M HCl in dioxane, rt; (c) 11, 1-chloro-N,N,2-trimethylprop-1-en-1-amine, DIEA, DCM, rt; (d) Me_3SnOH, DCE, 7.6%.

Suzuki reaction between 34 and 35 followed by removal of tert-butyl moiety afforded 36. Coupling with aniline followed by hydrolysis of methyl ester gave compound 6.

Experimental procedure for synthesis of compound 6.

Synthesis of 2-(2-(2-(phenylcarbamoyl)-2,3-dihydro-1H-inden-4-yl)-5-((4-(trifluoromethyl)phenyl)carbamoyl)thiazol-4-yl)acetic acid (**6**)

To a solution **34** (0.155 g, 0.37 mmol), 35 (0.151 g, 0.44 mmol), Pd(dppf)Cl_2·CH_2Cl_2 (0.03 g, 0.037 mmol) in 1,4-Dioxane (3.00 mL) was added Cs_2CO_3 (0.298 g, 0.46 mL, 0.92 mmol). The resulting mixture was degassed, refilled with nitrogen, and heated at 60 ^oC for 2 h. Then the reaction mixture was diluted with ethyl acetate, washed with water, dried with $MgSO_4$, filtered, concentrated, and purified by silica gel column chromatography using 0-20% EtOAc in hexanes to afford 36 (0.150 g, 0.268 mmol, 73.1%).

A mixture of hydrogen chloride (1.00 mL, 4.00 mmol, 4M in dioxane)) and 36 (0.115 g, 0.21 mmol) was stirred for 2 h. The reaction mixture was evaporated to dryness and taken to the next step without purification.

To a solution of 37 (0.030 g, 0.059 mmol) in DCM (1.0 mL) was added 1-chloro-N,N,2-trimethylprop-1-en-1-amine (0.009 g, 0.064 mmol) and stirred at rt for 15 minutes. To the resulting brown solution was added aniline (0.006 g, 0.064.4 mmol) followed by DIEA (0.023 mL,

0.129 mmol) and stirred at rt for 1 h. Then the reaction mixture was quenched with a solution of aqueous 1 N HCl and diluted with DCM. The organic layer was separated and the precipitate from the aqueous layer was filtered and washed with water to afford **38**, which was taken to the next step without purification.

To a solution of **38** (0.014.g, 0.024 mmol) in 1,2-Dichloroethane (1 mL) was added trimethyltin(IV) hydroxide (0.009 g, 0.048 mmol) and heated at 50 °C for 6 h. Then the reaction mixture was concentrated to dryness and purified by reverse phase HPLC to obtained **6** (0.010 g, 0.016 mmol, 7.6%). HRMS (ESI): m/z calcd for **6** (C29H22F3N3O4S + H)$^+$, 566.1356; found, 566.136.

^{1}H NMR (600 MHz, DMSO) δ 10.71 (bs, 1H), 10.15 (s, 1H), 7.91 (d, *J* = 8.5 Hz, 2H), 7.87 (d, *J* = 7.7 Hz, 1H), 7.73 (d, *J* = 8.6 Hz, 2H), 7.67 – 7.61 (m, 2H), 7.44 (d, *J* = 7.4 Hz, 1H), 7.38 (t, *J* = 7.6 Hz, 1H), 7.34 – 7.29 (m, 2H), 7.08 – 7.02 (m, 1H), 4.14 (s, 2H), 3.64 (dd, *J* = 16.1, 8.4 Hz, 1H), 3.53 (p, *J* = 8.1 Hz, 1H), 3.47 (dd, *J* = 16.0, 7.4 Hz, 1H), 3.31 (dd, *J* = 16.2, 8.1 Hz, 1H), 3.25 (dd, *J* = 16.2, 8.1 Hz, 1H). ^{13}C NMR (151 MHz, DMSO) δ 173.07, 171.10, 165.96, 159.91, 154.24, 144.35, 142.20, 140.28, 139.28, 128.78, 128.49, 127.65, 126.78, 126.69, 126.02 (q, *J* = 3.9 Hz), 125.87, 124.35 (q, *J* = 271.3 Hz), 124.05 (q, *J* = 32.2 Hz), 123.25, 120.46, 119.22, 44.68, 37.66, 36.80, 36.44.

2-(2-((1-benzoylpyrrolidin-3-yl)(phenyl)amino)-5-((4-(trifluoromethyl)phenyl)carbamoyl)thiazol-4-yl)acetic acid (7)

Scheme 6. Synthesis of compound 7.

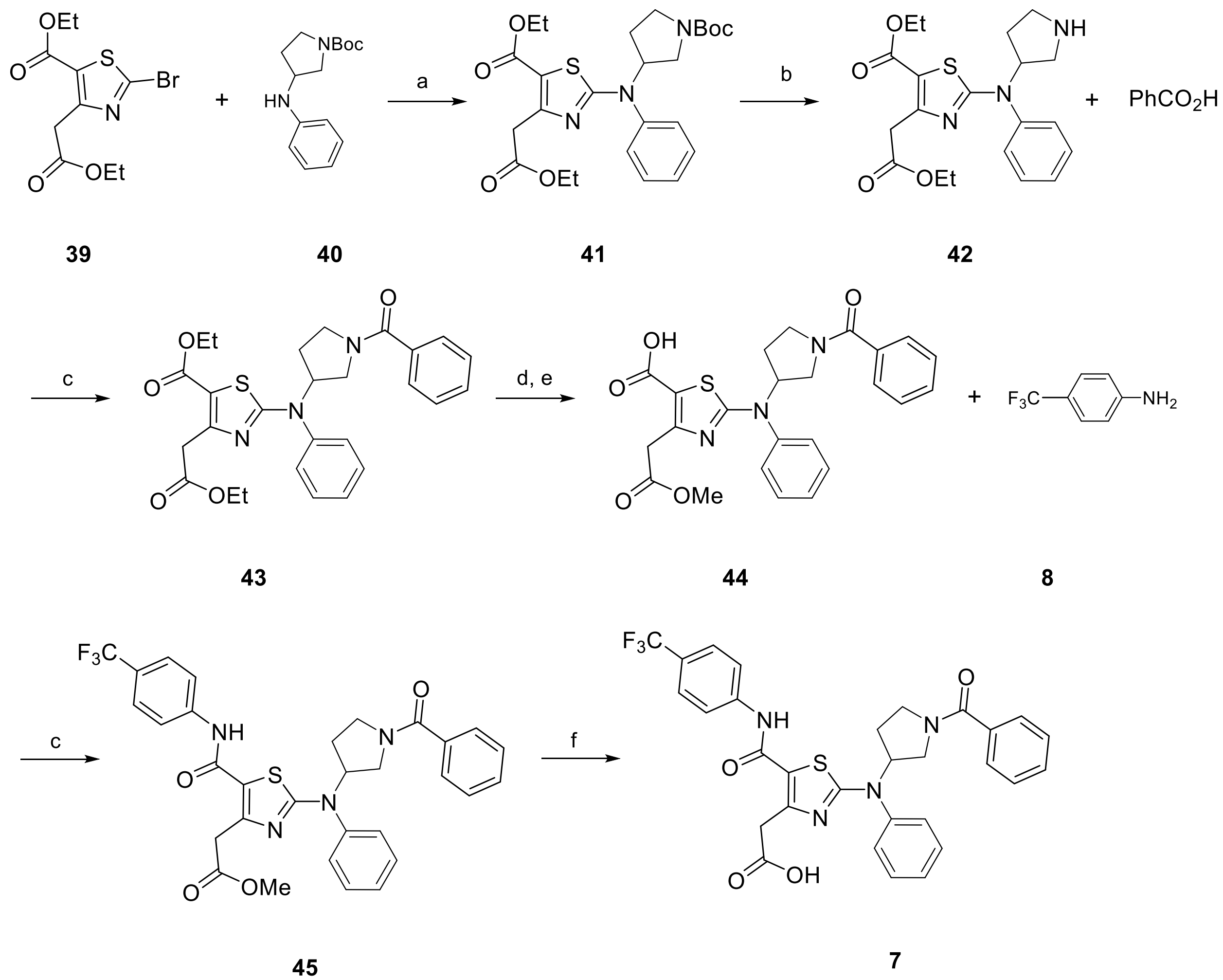

Reagents and conditions: (a) Pd-PEPPSI-IpentCl, Cs_2CO_3, dioxane, yield 51.2%; (b) TFA, DCM; (c) TCFH, NMI, DMF, yield 57.2%; (d) LiOH, MeOH, H_2O, yield 50%; (e) HCl, MeOH, yield 62.1%; (f) TCFH, NMI, DMF, yield 25%; (g) Me_3SnOH, DCE, yield 71.7%.

Palladium mediated C-N coupling between **39** and **40** followed by removal of Boc led to formation of **42**. Amide Coupling of resulting free amine of **42** with benzoic acid provided **43**. Bis de-esterification followed by selective methyl ester formation led to formation of **44**. Finally, amide coupling between **44** and **8** and subsequent hydrolysis of methyl ester of **45** mediated trimethyltin hydroxide afford **7**.

Experimental procedure for synthesis of compound 7.

Synthesis of ethyl 2-((1-(tert-butoxycarbonyl)pyrrolidin-3-yl)(phenyl)amino)-4-(2-ethoxy-2-oxoethyl)thiazole-5-carboxylate (**42**)

A mixture of **39** (3.98 g, 12.4 mmol), **40** (3.91 g, 14.90 mmol), Pd-PEPPSI-IpentCl (2.14 g, 2.5 mmol) and Cs_2CO_3 (12.14 g, 37.2 mmol) in dioxane (80 mL) was stirred at 110 °C for 8 h under N_2 atmosphere. The crude product was concentrated and directly loaded into column and

purified by silica gel chromatography using 0-50% EtOAc in hexanes to give **41** (3.19 g, 6.35 mmol, 51.2% yield) as a colorless oil. MS (ESI): *m/z* $(M+H)^+$ 504.3.

Synthesis of ethyl 4-(2-ethoxy-2-oxoethyl)-2-(phenyl(pyrrolidin-3-yl)amino)thiazole-5-carboxylate (**42**)

To a stirred solution of **41** (2.49 g, 4.96 mmol) in DCM (25 mL) was added TFA (5 mL). The mixture was stirred at 25 °C for 3 h. The reaction mixture was concentrated in vacuo to give **42** (1.99 g, 4.96 mmol, ~100% yield) as white oil. The crude product was used in the next step directly. MS (ESI): *m/z* $(M+H)^+$ 404.3.

Synthesis of ethyl 2-((1-benzoylpyrrolidin-3-yl)(phenyl)amino)-4-(2-ethoxy-2-oxoethyl)thiazole-5-carboxylate (**43**)

To a stirred solution of **42** (0.99 g, 2.48 mmol) in DMF (15 mL) was added benzoic acid (0.908 g, 7.43 mmol), TCFH (1.043 g, 3.72 mmol) and NMI (0.407 g, 4.96 mmol) at 25 °C and the resulting reaction mixture was stirred at 25 °C for 16 h. The reaction mixture was diluted with water (50 mL) and extracted with EtOAc (3 × 20 mL). The combined organic layer was washed with brine (30 mL), dried over anhydrous Na_2SO_4, filtered, and concentrated in vacuo. The residue was purified by silica gel chromatography using 75% EtOAc in hexanes to give **43** (0.90 g, 1.42 mmol, 57.2% yield) as a white solid. MS (ESI): *m/z* $(M+H)^+$ 508.3.

Synthesis of 2-((1-benzoylpyrrolidin-3-yl)(phenyl)amino)-4-(2-methoxy-2-oxoethyl)thiazole-5-carboxylic acid (**44**)

To a mixture of **43** (0. 90 g, 1.78 mmol) in a mixture of MeOH (10 mL) and water (5 mL) was added LiOH (0.425 g, 17.73 mmol) and stirred at 25 °C for 16 h. The mixture was diluted with water (20 mL) and EtOAc (30 mL). The organic layer was separated, and the aqueous layer was acidified with 1 N HCl to pH < 7. The aqueous layer was re-extracted with EtOAc (30 mL x 3), the combined organic layers were washed with brine (30 mL x 2), dried over anhydrous Na_2SO_4, filtered, and concentrated under reduced pressure to give the corresponding bis acid (0.50 g, 0.89 mmol, 50.0% yield) as white oil. The crude product was used in the next step directly. MS (ESI): *m/z* $(M+H)^+$ 452.2.

A mixture of this bis acid (0.50 g, 1.11 mmol) and HCl (2 mL, 24.36 mmol) in MeOH (10 mL) was stirred at 20 °C for 8 h. The mixture was purified by reverse phase HPLC to give **44** (0.319 g, 0.687 mmol, 62.1% yield) as a white solid. MS (ESI): *m/z* $(M+H)^+$ 466.2.

Synthesis of methyl 2-(2-((1-benzoylpyrrolidin-3-yl)(phenyl)amino)-5-((4-(trifluoromethyl)phenyl)carbamoyl)thiazol-4-yl)acetate (**45**)

To a stirred solution of **44** (0.33 g, 0.71 mmol) in DMF (5 mL) was added 4-(trifluoromethyl)aniline **8** (0.343 g, 2.18 mmol), TCFH (0.298 g, 1.06 mmol) and NMI (0.116 g, 1.42 mmol) at 25 °C and stirred at 25 °C for 16 h. The mixture was purified by reverse HPLC to give **45** (0.120 g, 0.18 mmol, 25% yield) as a white solid. MS (ESI): *m/z* $(M+H)^+$ 609.2. ^{1}H NMR (CD_3OD,

400 MHz): δ 7.66-7.75 (m, 2H), 7.53-7.64 (m, 5H), 7.33-7.51 (m, 7H), 5.12 (br t, *J* = 5.8 Hz, 1H), 3.94-4.13 (m, 3H), 3.38-3.88 (m, 6H), 2.02-2.45 (m, 2H).

2-(2-((1-benzoylpyrrolidin-3-yl)(phenyl)amino)-5-((4-(trifluoromethyl)phenyl)carbamoyl)thiazol-4-yl)acetic acid (**7**)

To a mixture of **45** in DCE (2 mL) was added trimethylstannanol (0.107 g, 0.59 mmol) and stirred at rt for 3 h. The reaction mixture was purified by reverse phase HPLC to give **7** (0.085 g, 0.14 mmol, 71.7% yield) as a white solid. ^{1}H NMR (600 MHz, DMSO, sample is a mixture of two rotamers in ratio 53:47) δ 12.4 (bs, 1H), 9.89 (s, 0.53H), 9.85 (s, 0.47H), 7.82 – 7.75 (m, 2H) , 7.66 – 7.49 (m, 6H), 7.48 – 7.32 (m, 6H), 5.25 (p, *J* = 6.9 Hz, 0.54H), 5.08 (p, *J* = 6.1 Hz, 0.47H), 3.98 (s, 1H), 3.97 – 3.91 (m, 1H), 3.90 – 3.80 (m, 1H), 3.56 (dd, *J* = 12.5, 6.5 Hz, 0.53H), 3.50 (dd, *J* = 11.3, 5.3 Hz, 0.47H), 3.46 – 3.39 (m, 1H), 3.38 – 3.22 (m, 0.47H), 3.12 – 3.07 (m, 0.53H), 3.34 – 3.20 (m, 1H), 2.10 – 2.00 (m, 0.47H), 1.95 – 1.85 (m, 0.53H). ^{13}C NMR (151 MHz, DMSO, sample is a mixture of two rotamers in ratio 53:47) δ 171.07, 170.99, 169.95, 169.68, 168.35, 167.96, 160.14, 160.08, 153.68, 153.60, 142.50, 142.48, 140.41, 140.27, 136.41, 136.34, 130.61, 130.58, 130.05, 129.76, 129.73, 129.66, 129.59, 128.08, 128.06, 126.88, 126.78, 125.58, 124.26 (q, *J* = 270.7 Hz), 123.21 (q, *J* = 32.3 Hz), 123.20 (q, *J* = 32.3 Hz), 120.15, 120.12, 114.21, 114.18, 58.74, 57.00, 51.60, 48.69, 46.77, 43.94, 37.01, 36.96, 29.75, 28.07.

HRMS (ESI): m/z calcd for **7** (C30H25F3N4O4S + H)$^{+}$, 595.1622; found, 595.1627..
