# Supplementary material for: WRN structural flexibility showcased through fragment-based lead discovery of inhibitors
Source: Nat Commun. 2026 Jan 3;17:79. doi: 10.1038/s41467-025-66768-8 (PMC12770528; doi:10.1038/s41467-025-66768-8)

WRN structural flexibility showcased through fragment-based lead discovery of inhibitors

Rachel L. Palte[1‡], Mihir Mandal[2‡], Justyna Sikorska[3‡], Artjohn B. Villafania[3‡], Meredith M. Rickard[1], Robert J. Bauer[4], Xiaomei Chai[4], Jiafang He[2], Zahid Hussain[2], Markus Koglin[3], Hannah B. MacDonald[5], My S. Mansueto[4], Klaus Maskos[6], Joey L. Methot[1], Aileen Soriano[3], Jaclyn Robustelli[3], Alexei V. Buevich,[8] Marcel J. Tauchert[6], Sriram Tyagarajan[4], Minjia Zhang[4], Daniel J. Klein[7], Jacqueline D. Hicks[2], David G. McLaren[3], Sandra B. Gabelli[7]*, Daniel F. Wyss[3]*

[1]Discovery Chemistry, Merck & Co., Inc., MRL, Boston, MA 02115, USA
[2]Discovery Chemistry, Merck & Co., Inc., MRL, Rahway, NJ 07065, USA
[3]Quantitative Biosciences, Merck & Co., Inc., MRL, Rahway, NJ 07065, USA
[4]Quantitative Biosciences, Merck & Co., Inc., MRL, Boston, MA 02115, USA
[5]Discovery Chemistry London, MSD (UK) Ltd., London, UK
[6]Proteros Biostructures, Planegg-Martinsried, Germany
[7]Discovery Chemistry, Merck & Co., Inc., MRL, West Point, PA 19486, USA
[8]Analytical Research & Development, Merck & Co., Inc., Rahway, NJ 07065, USA
‡ Shared first authors
*Shared corresponding authors

Contents **page number**

- HRMS of fragments **2**- **4** and compounds **1**-**7** 3-12
- LCMS of fragments **1**- **4** and compounds **3**-**7** 13-23
- 1H and 13C listing for fragments 24
- 1H and 13C spectra for fragments and compounds 25-59

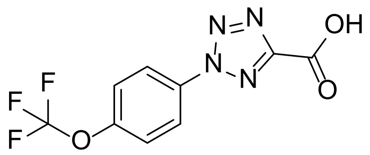

Exact Mass: 274.03

Fragment 2

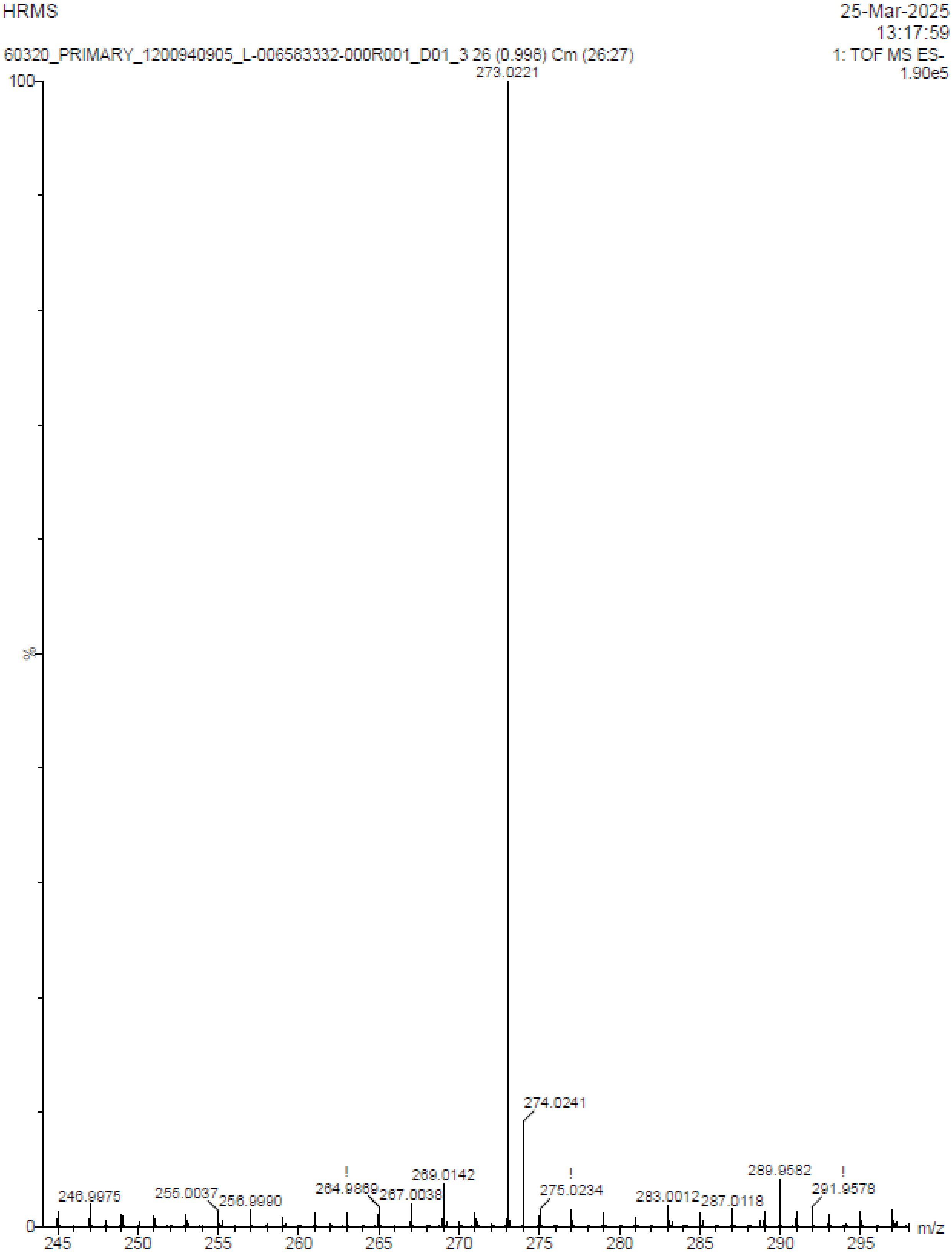

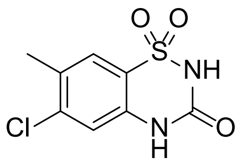

Exact Mass: 245.99

Fragment 3

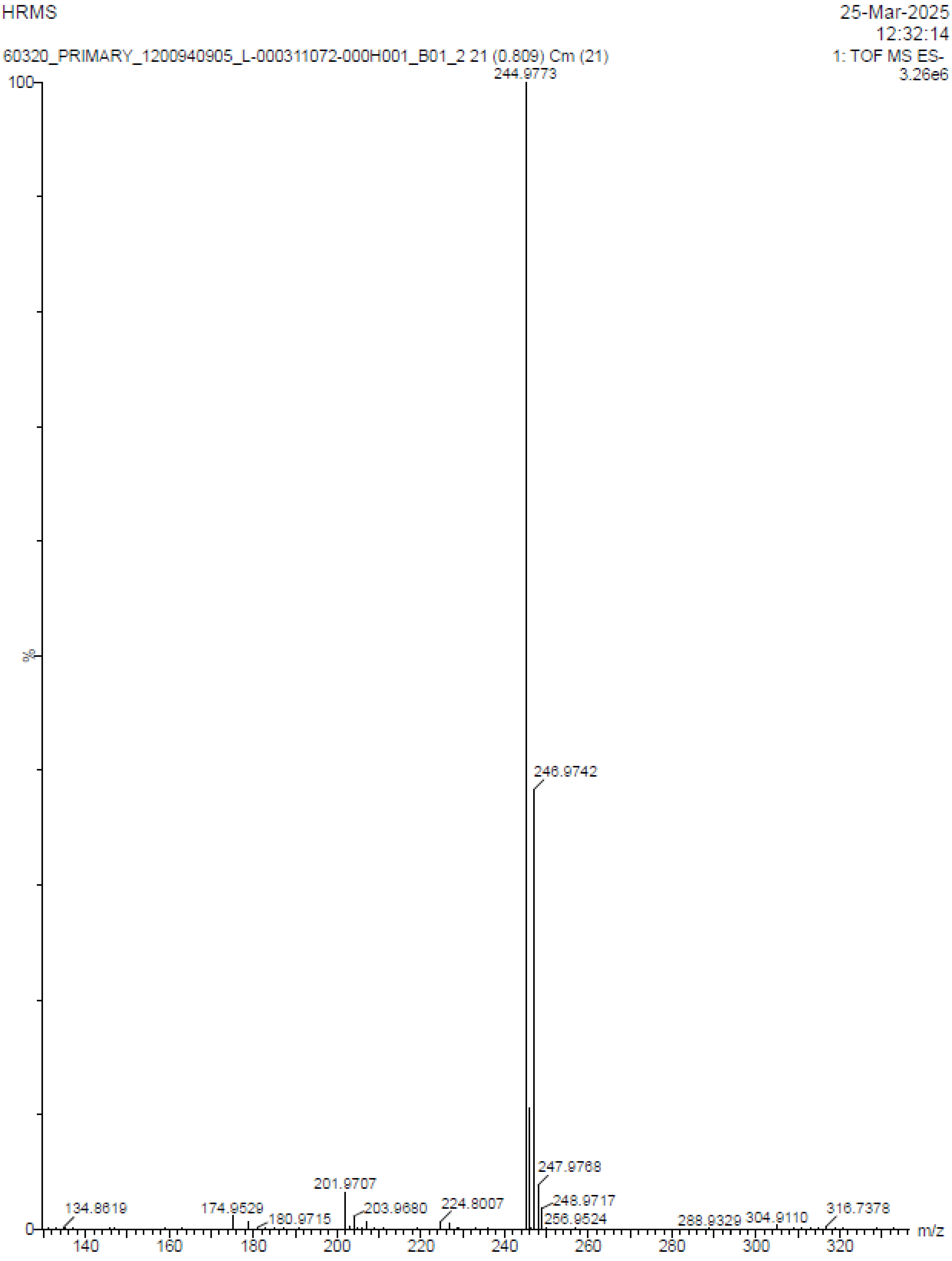

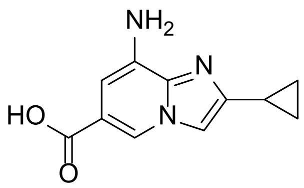

Exact Mass: 217.09

Fragment 4

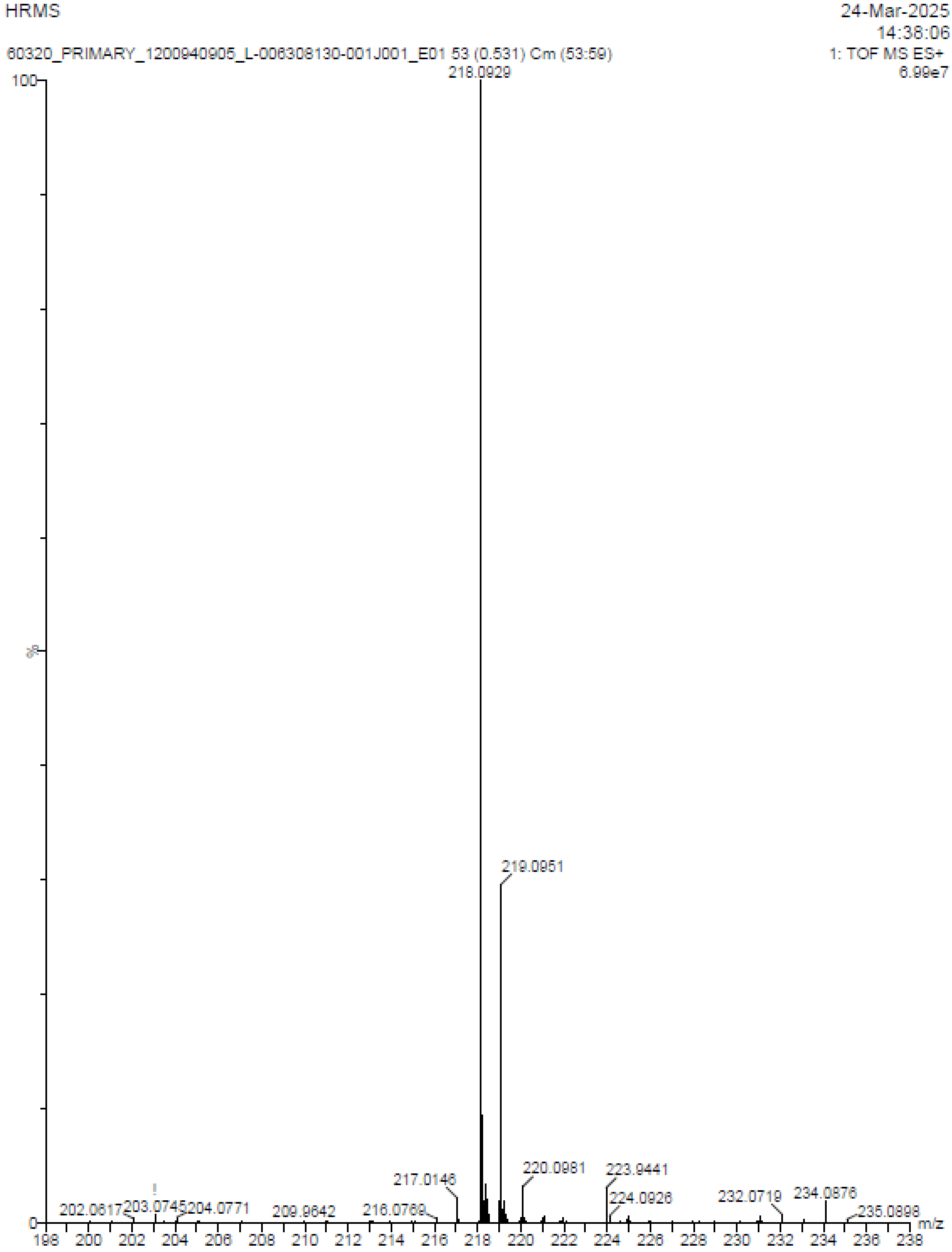

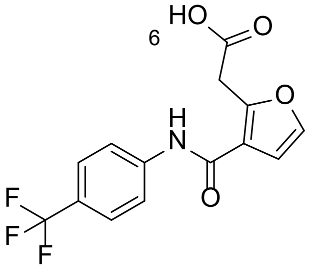

Exact Mass: 313.06

Compound 1

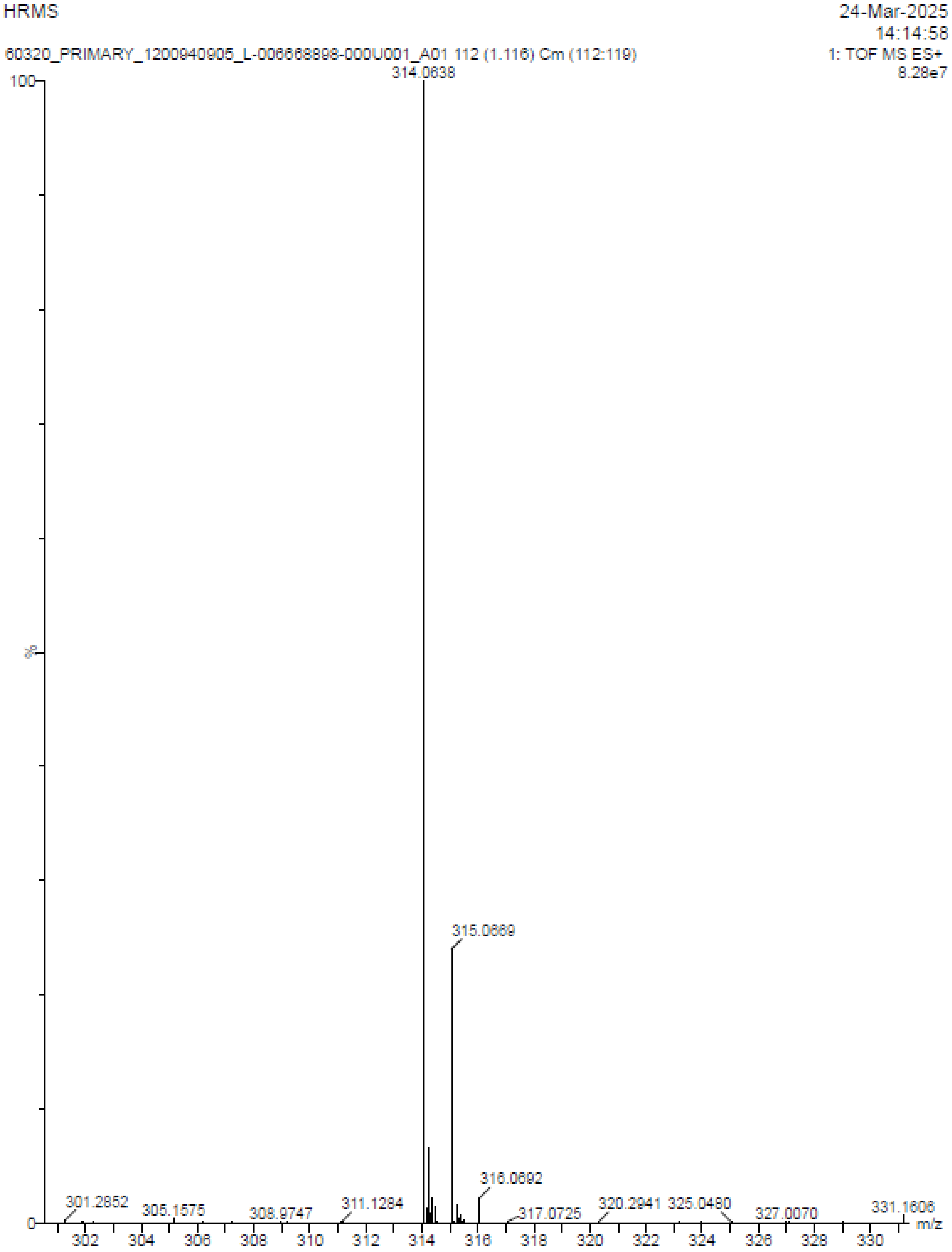

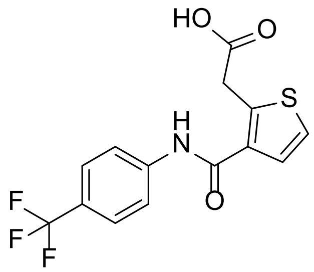

Exact Mass: 329.03

Compound 2

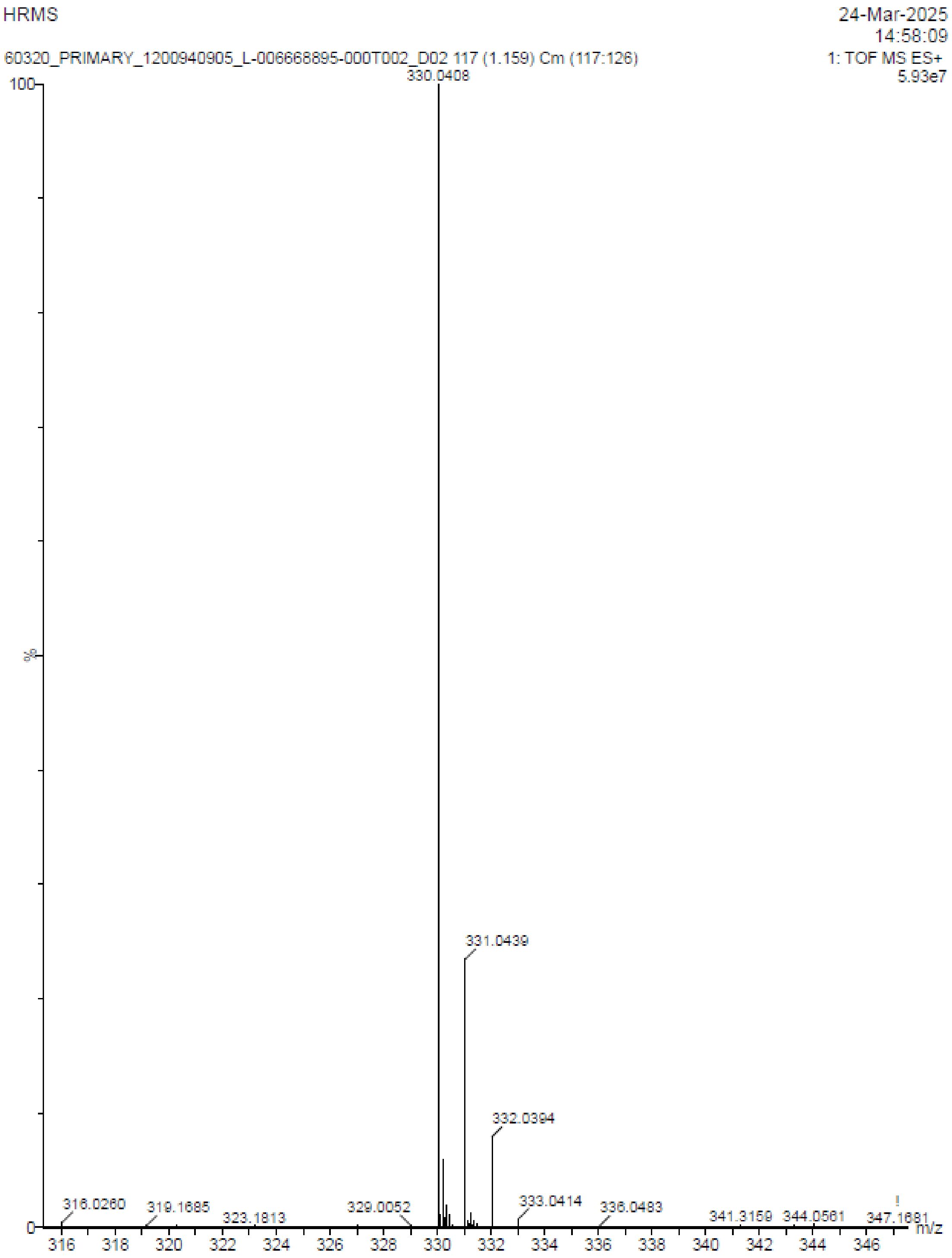

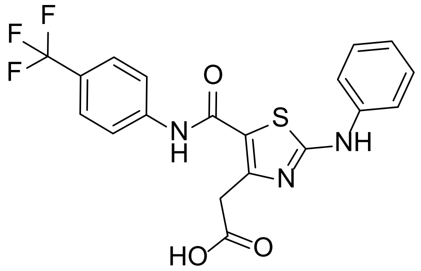

Exact Mass: 421.07

Compound 3

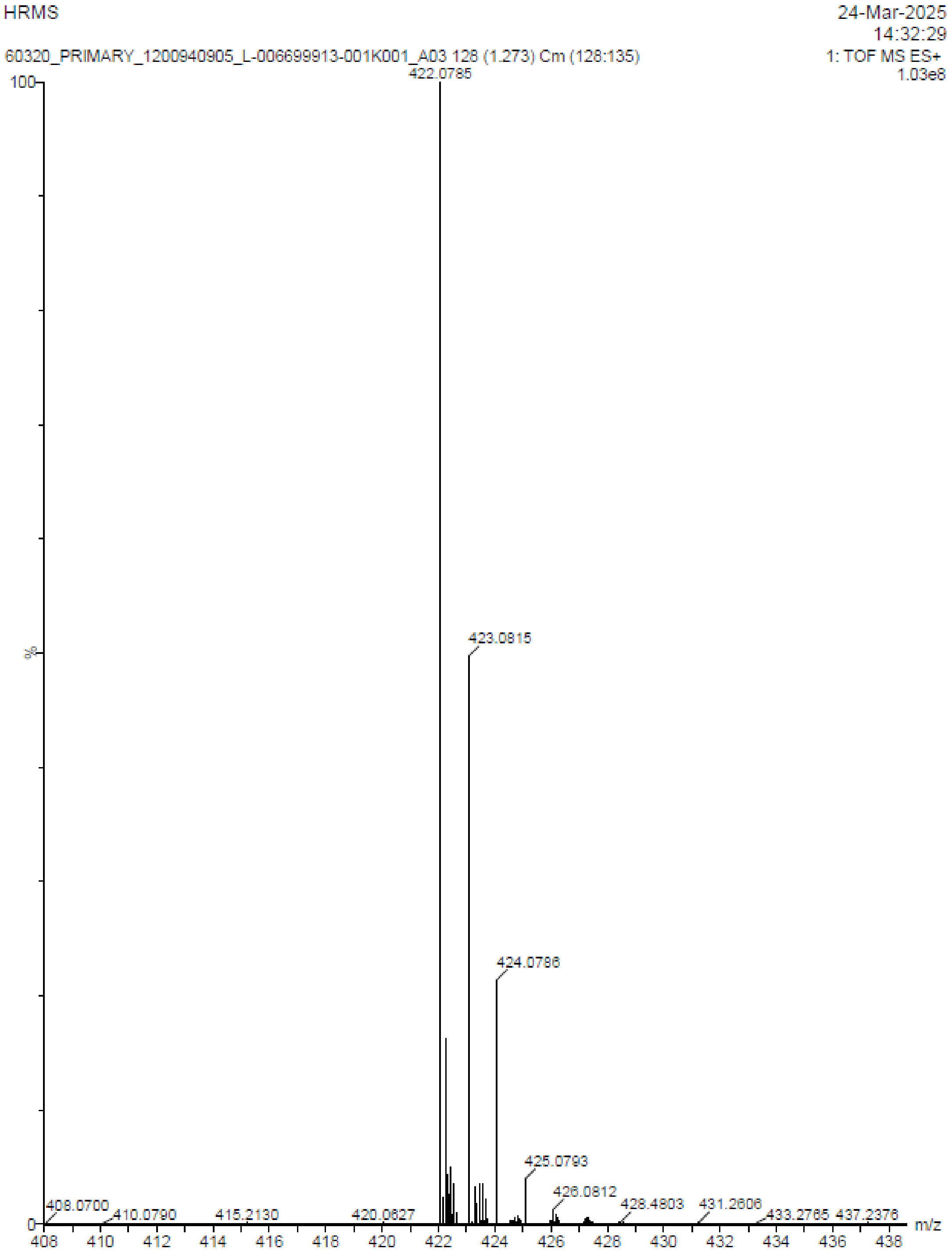

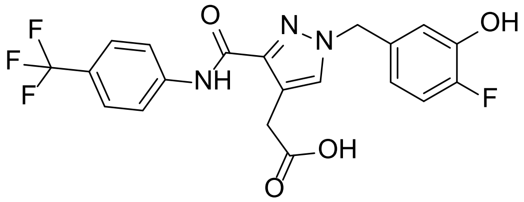

Exact Mass: 437.10

Compound 4

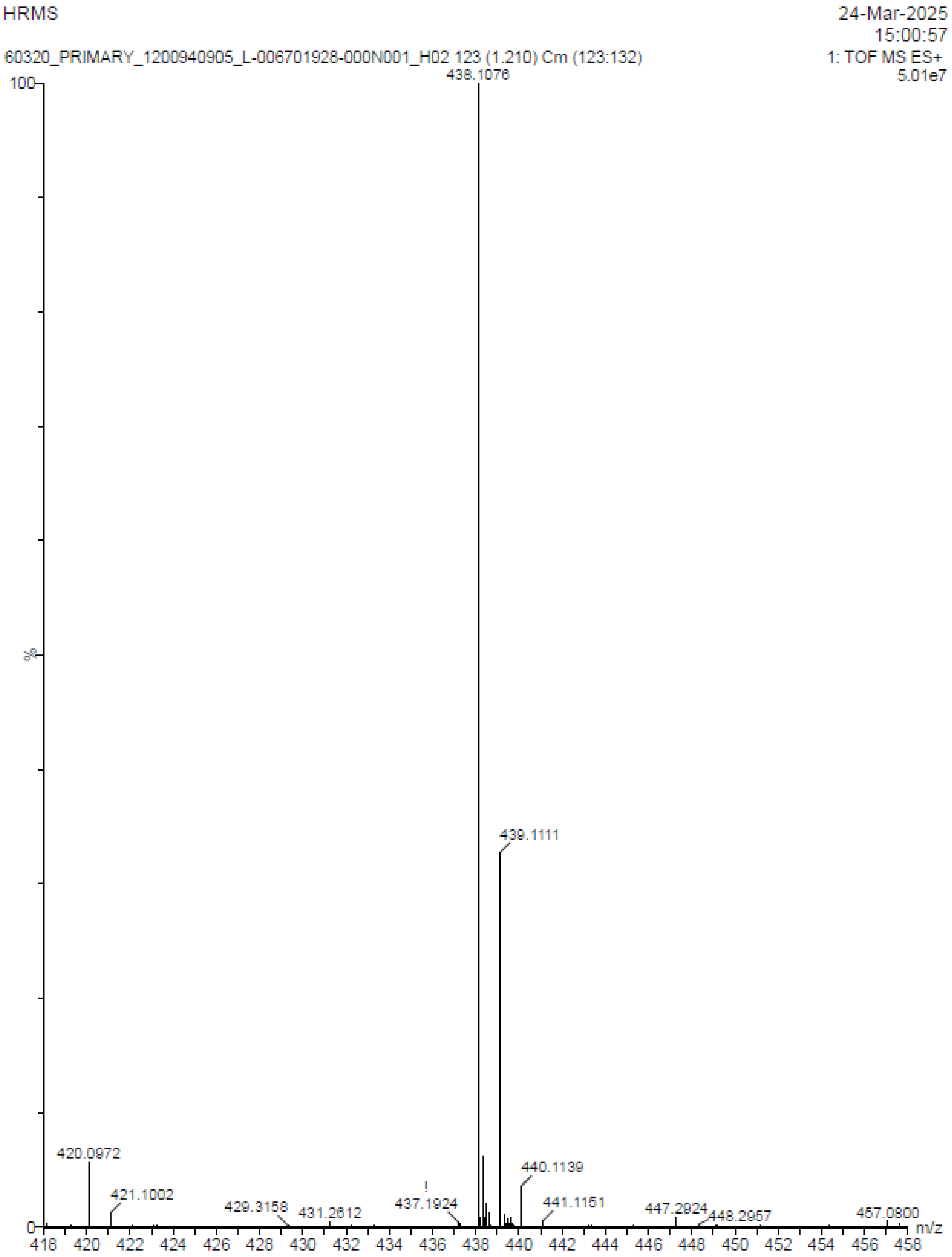

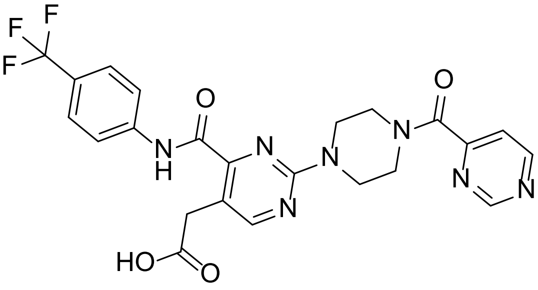

Exact Mass: 515.15

Compound 5

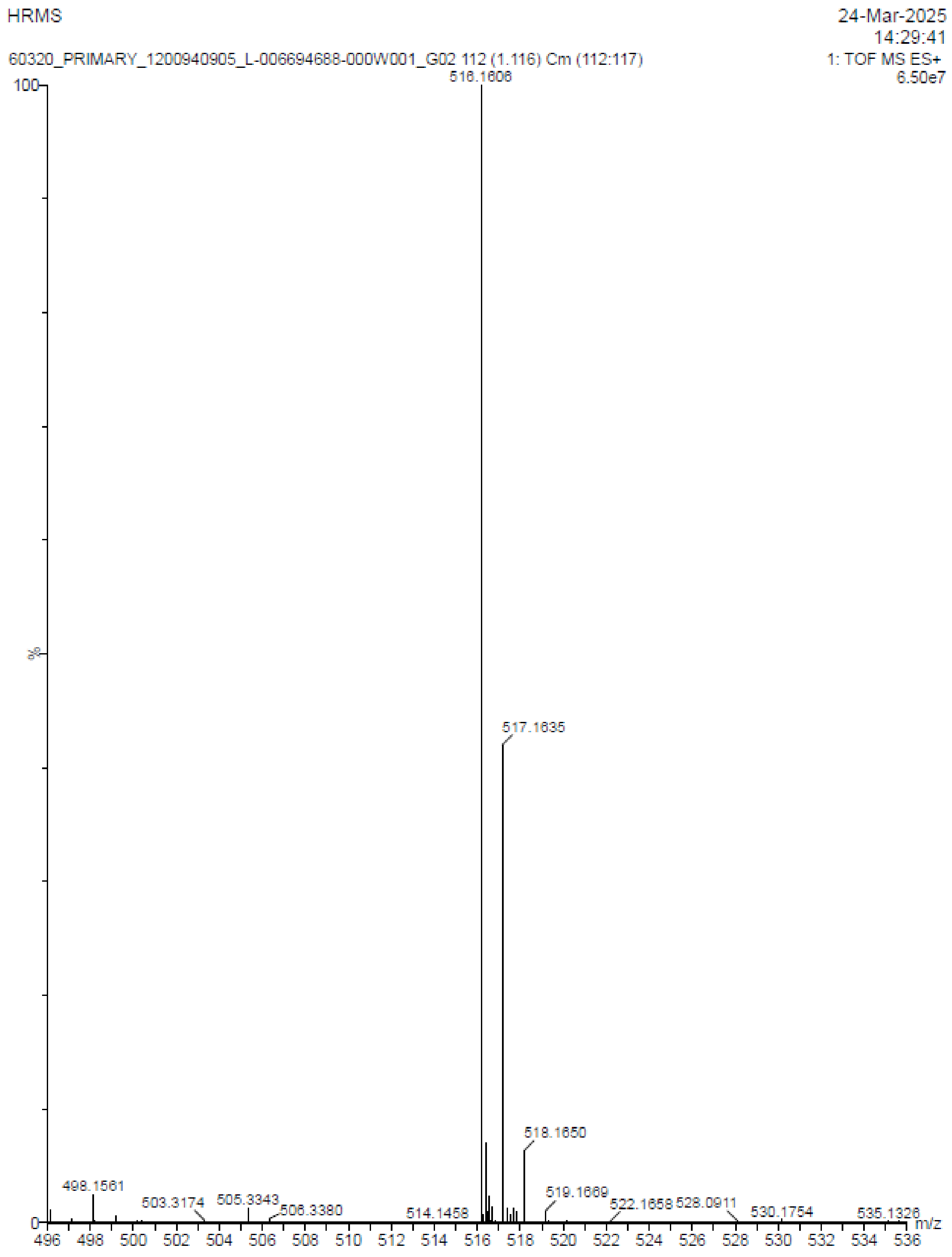

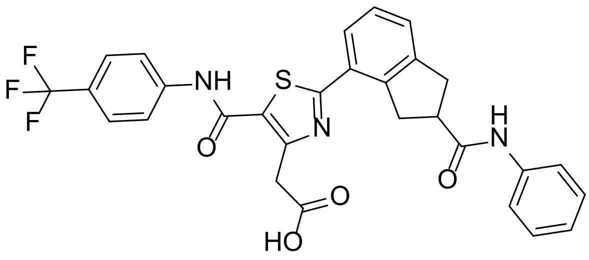

Exact Mass: 565.13

Compound 6

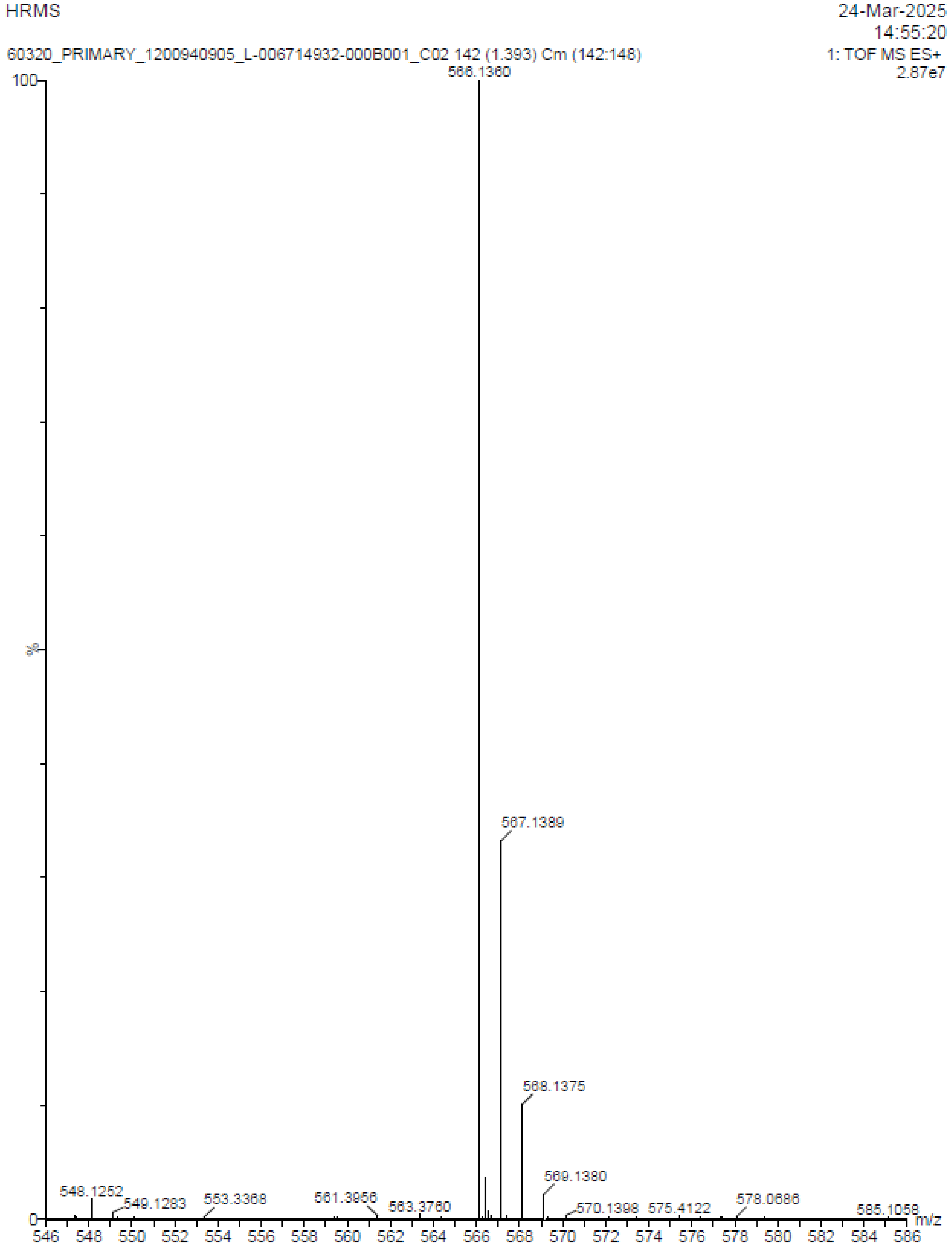

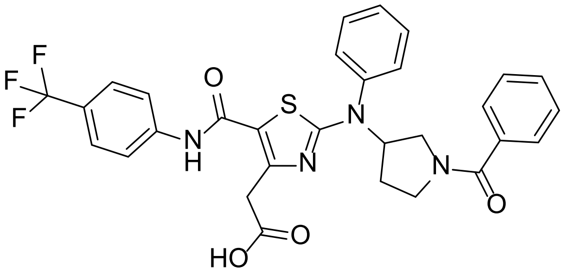

Exact Mass: 594.15

Compound 7

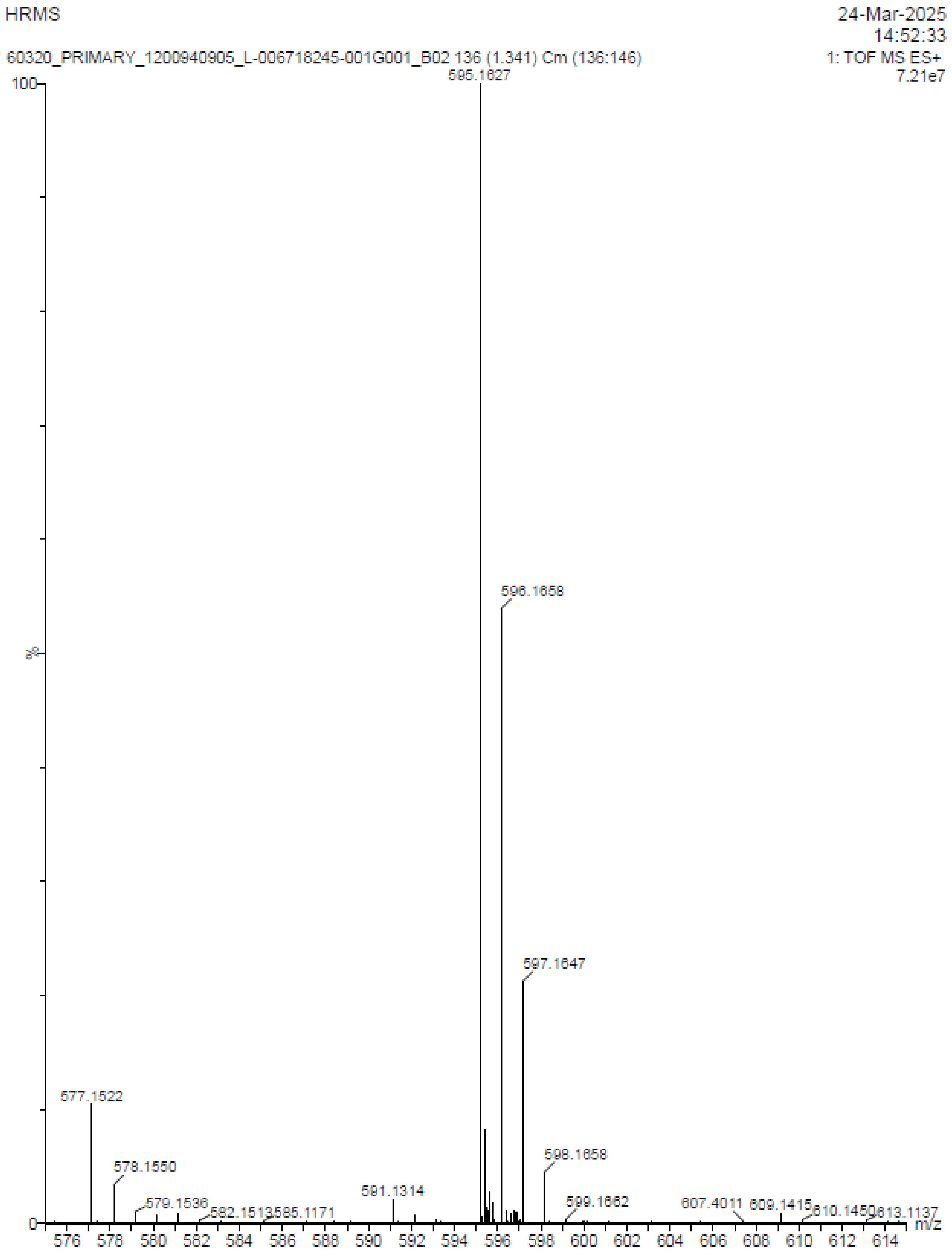

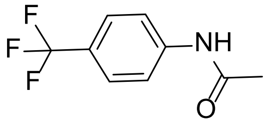

Fragment 1

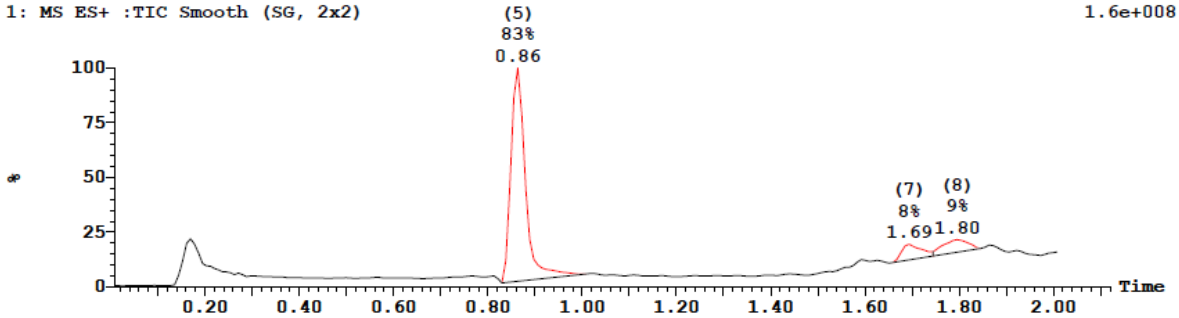

Peak Number	Compound	Time	AreaAbs	Area %Total	Width	Height	Mass Found
5		0.86	6e+006	82.92	0	2e+008	Not Found
7		1.69	6e+005	8.15	0	1e+007	Not Found
8		1.80	6e+005	8.93	0	9e+006	Not Found

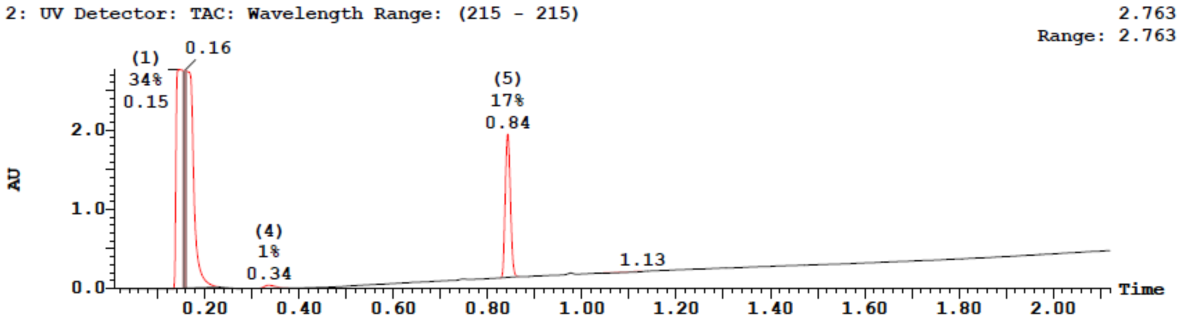

Peak Number	Compound	Time	AreaAbs	Area %Total	Width	Height	Mass Found
1		0.15	5e+004	33.70	0	3e+006	Not Found
2		0.16	1e+004	10.14	0	3e+006	Not Found
3		0.16	5e+004	38.15	0	3e+006	Not Found
4		0.34	1e+003	0.78	0	4e+004	Not Found
5		0.84	2e+004	16.93	0	2e+006	Not Found
6		1.13	4e+002	0.30	0	6e+003	Not Found

5 0.86 Not Found

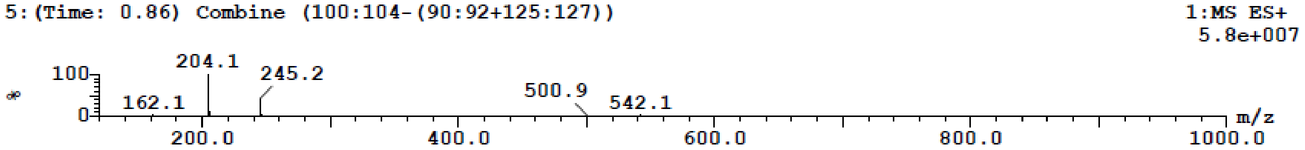

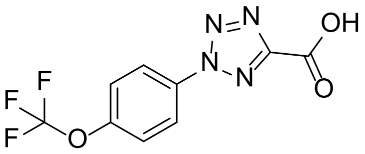

Fragment 2

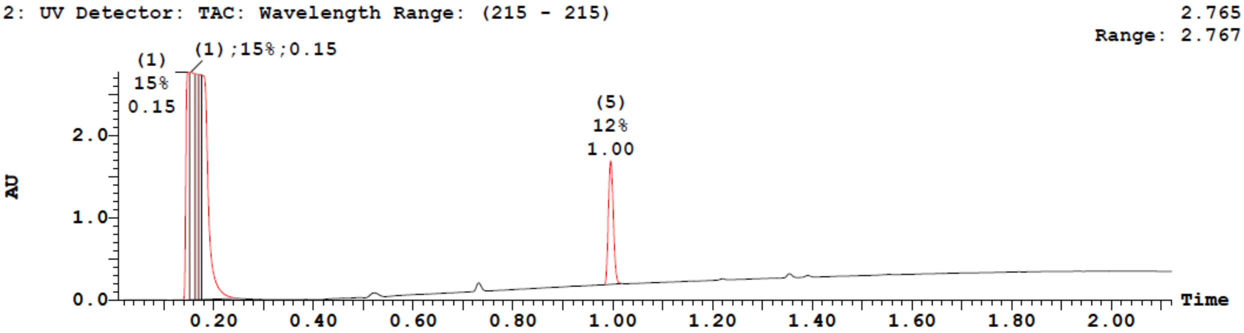

Peak Number	Time	Area %Total	Mass Found
1	0.15	14.87	Not Found
2	0.15	20.29	Not Found
3	0.17	13.96	Not Found
4	0.17	10.83	Not Found
4	0.18	28.32	Not Found
5	1.00	11.73	Not Found

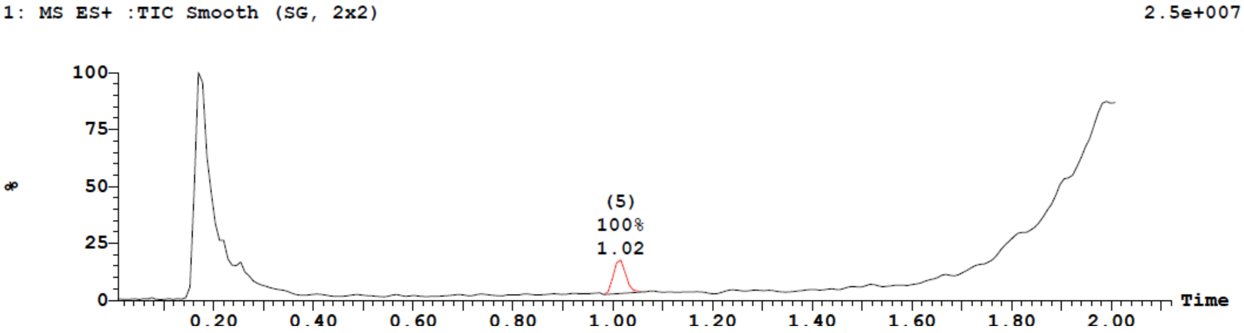

Peak ID	Time	Mass Found
5	1.02	Not Found

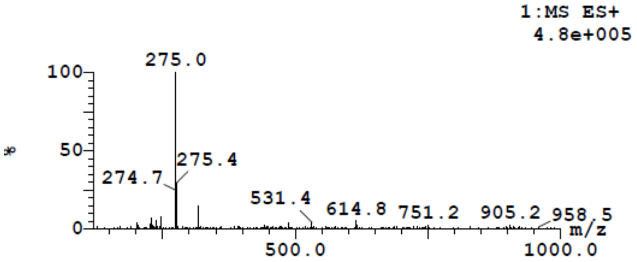

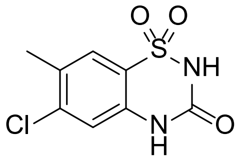

Fragment 3

1: MS ES+ :TIC Smooth (SG, 2x2) 2.3e+007

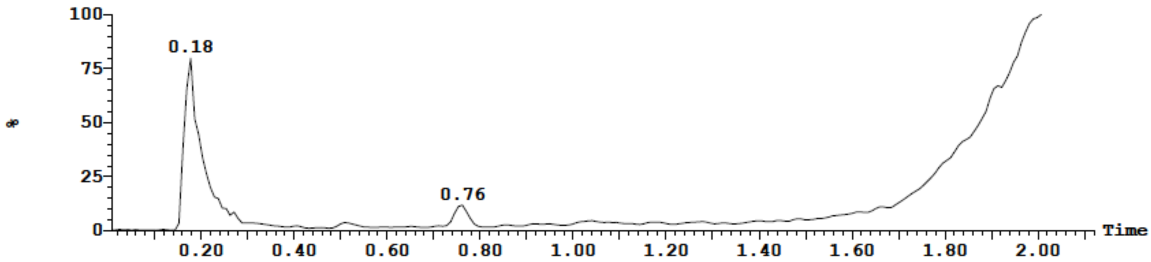

Peak Number	Compound	Time	AreaAbs	Area %Total	Width	Height	Mass Found

2: UV Detector: TAC: Wavelength Range: (215 - 215) 2.843

Range: 2.844

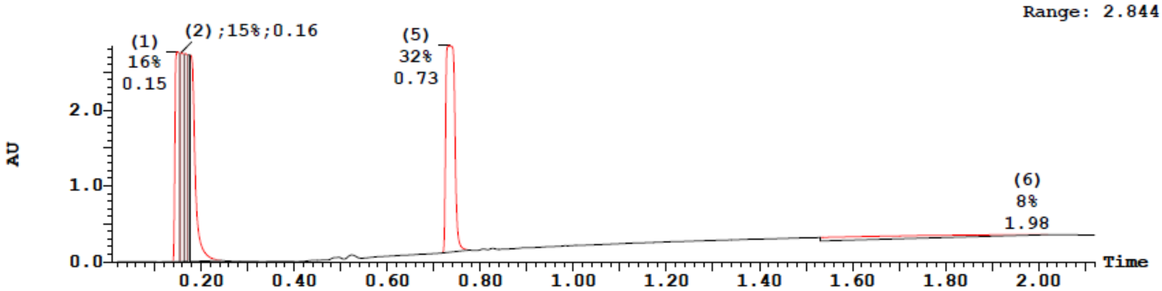

Peak Number	Compound	Time	AreaAbs	Area %Total	Width	Height	Mass Found
1		0.15	3e+004	16.07	0	3e+006	Not Found
2		0.16	3e+004	14.63	0	3e+006	Not Found
3		0.17	2e+004	10.93	0	3e+006	Not Found
4		0.18	3e+004	18.67	0	3e+006	Not Found
5		0.73	6e+004	32.10	0	3e+006	Not Found
6		1.98	1e+004	7.59	0	9e+003	Not Found

Peak ID	Compound	Time	Mass Found
5		0.73	Not Found

5: (Time: 0.73) Combine (84:89-(77:79+98:100)) 1:MS ES+

7.1e+004

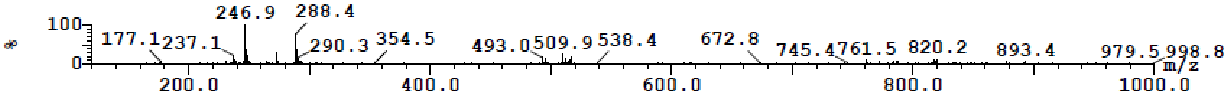

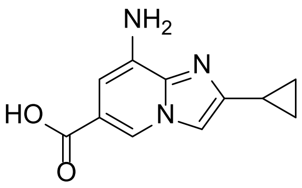

Fragment 4

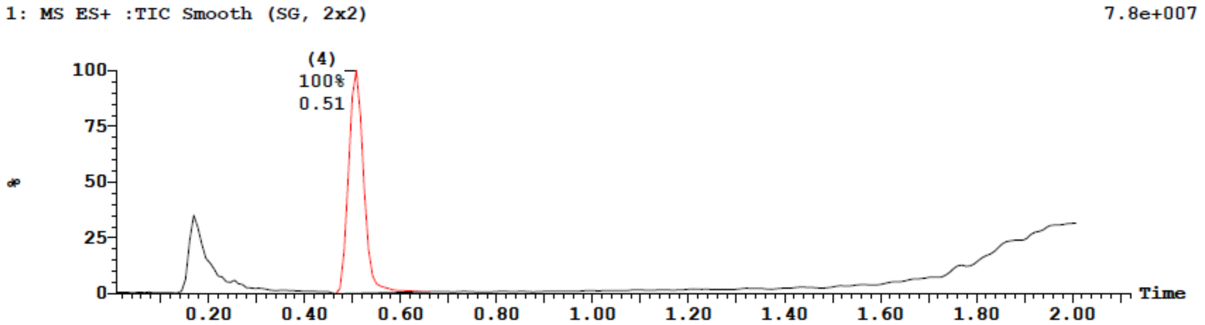

Peak Number	Compound	Time	AreaAbs	Area %Total	Width	Height	Mass Found
4		0.51	3e+006	100.00	0	8e+007	Not Found

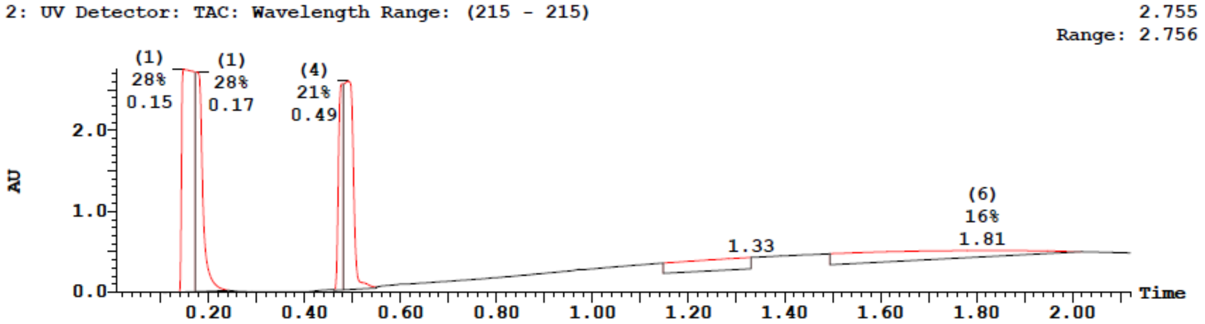

Peak Number	Compound	Time	AreaAbs	Area %Total	Width	Height	Mass Found
1		0.15	8e+004	27.96	0	3e+006	Not Found
2		0.17	5e+004	16.76	0	3e+006	Not Found
3		0.48	3e+004	9.66	0	3e+006	Not Found
4		0.49	6e+004	21.04	0	3e+006	Not Found
5		1.33	2e+004	8.74	0	1e+005	Not Found
6		1.81	4e+004	15.85	1	8e+004	Not Found

3 0.48 Not Found

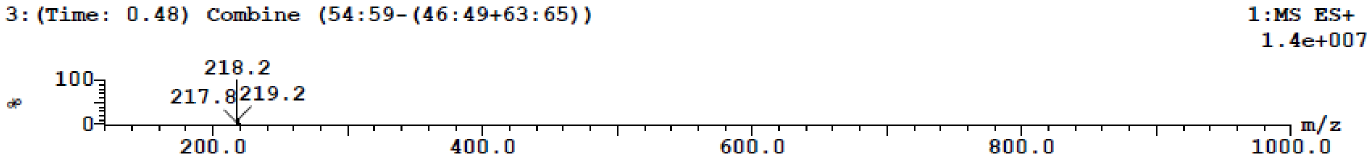

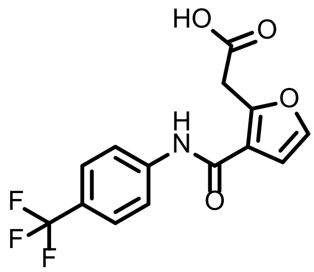

Exact Mass: 313.06

Compound 1

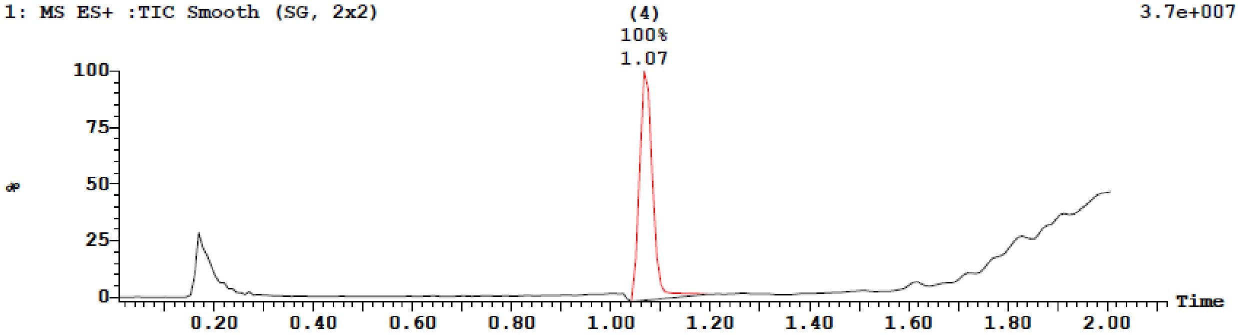

Peak Number	Compound	Time	AreaAbs	Area %Total	Width	Height	Mass Found
4		1.07	1e+006	100.00	0	4e+007	Not Found

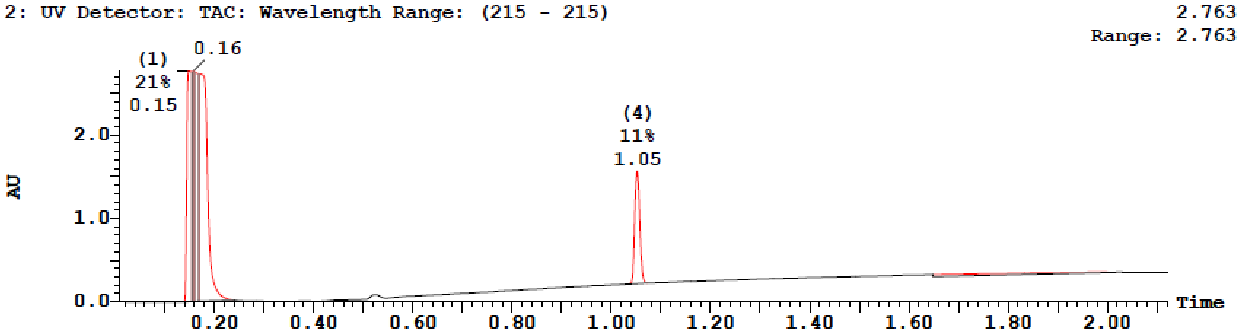

Peak Number	Compound	Time	AreaAbs	Area %Total	Width	Height	Mass Found
1		0.15	3e+004	21.24	0	3e+006	Not Found
2		0.16	1e+004	9.57	0	3e+006	Not Found
2		0.16	3e+004	17.46	0	3e+006	Not Found
3		0.17	5e+004	37.15	0	3e+006	Not Found
4		1.05	2e+004	10.91	0	1e+006	Not Found
5		2.02	5e+003	3.67	0	4e+002	Not Found

[illegible]

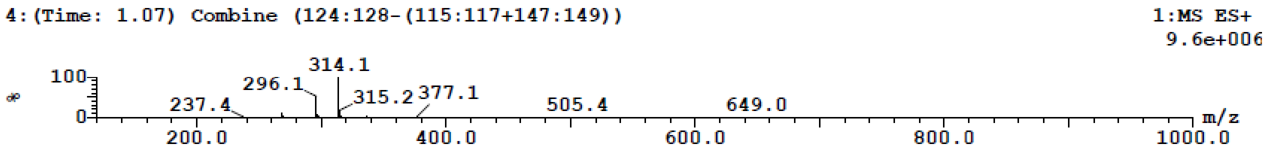

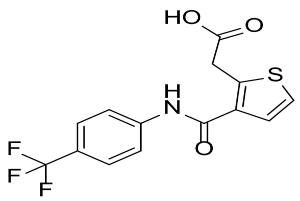

Exact Mass: 329.03

Compound 2

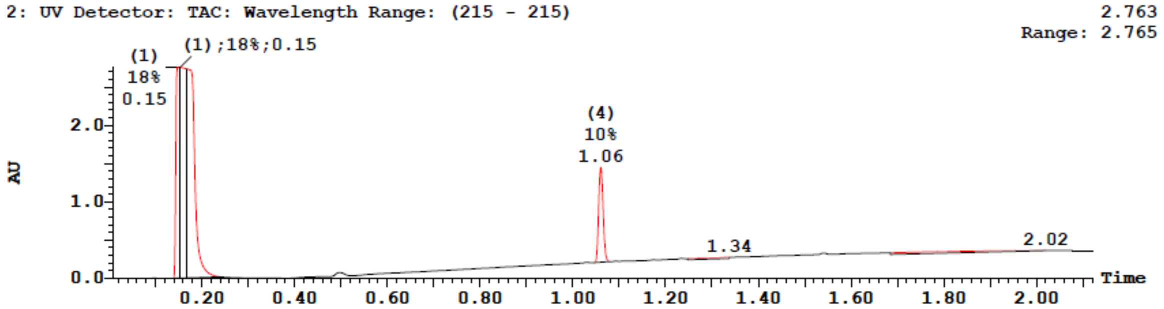

Peak Number	Time	Area %Total	Mass Found
1	0.15	17.72	Not Found
2	0.15	29.43	Not Found
3	0.17	38.48	Not Found
4	1.06	9.94	Not Found
5	1.34	0.86	Not Found
6	2.02	3.57	Not Found

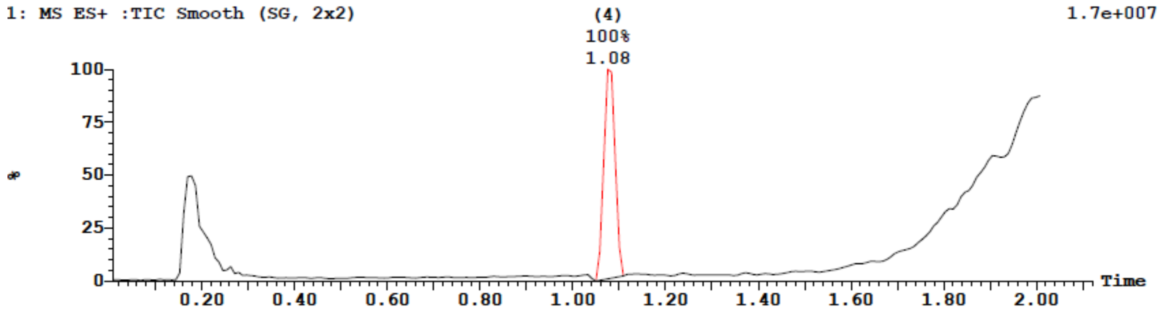

Peak ID	Time	Mass Found
4	1.08	Not Found

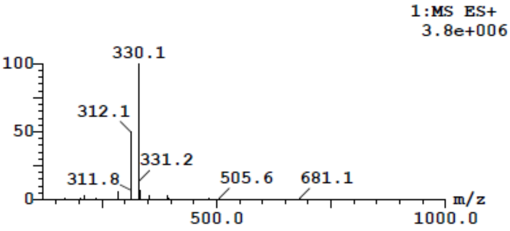

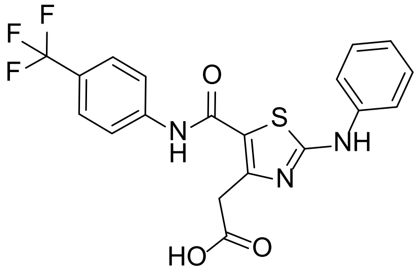
F
F
F
O
N
H
S
NH
N
HO
O

Compound 3

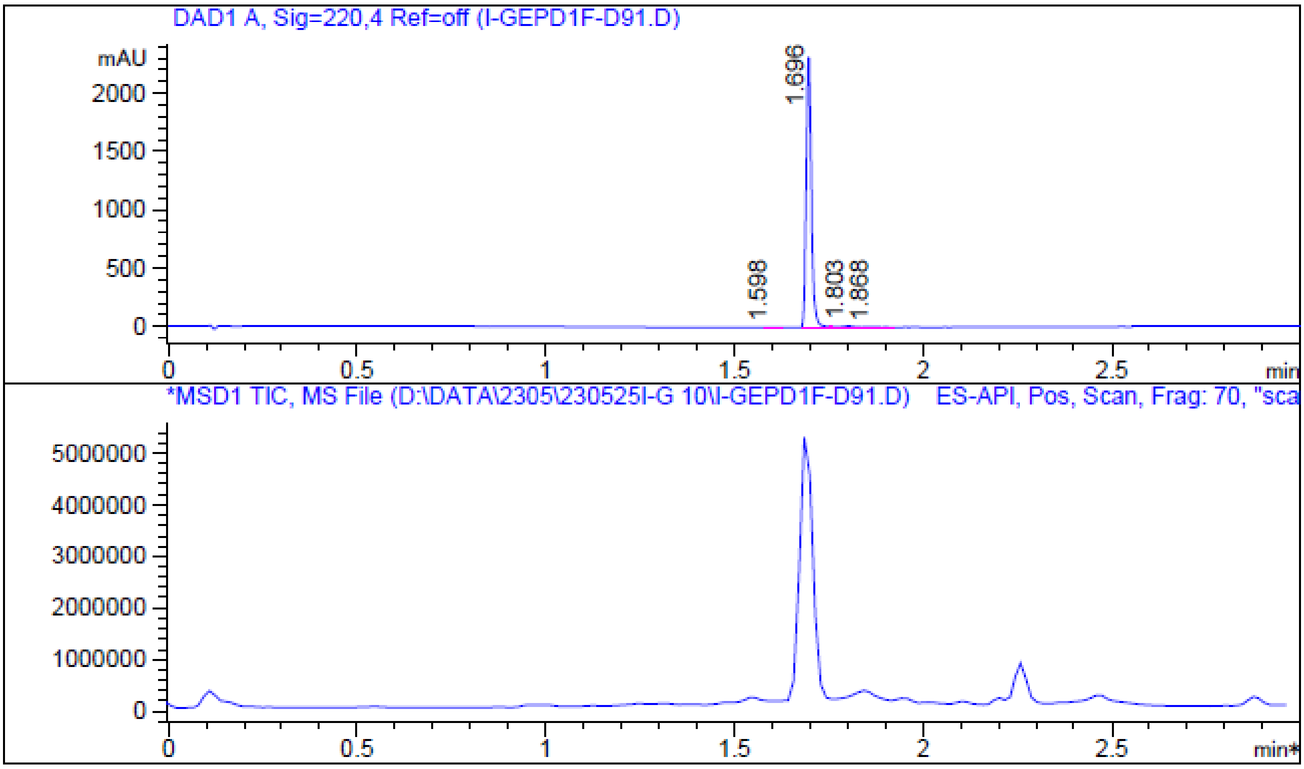
DAD1 A, Sig=220,4 Ref=off (I-GEPD1F-D91.D)
mAU
2000
1500
1000
500
0
1.696
1.598
1.803
1.868
0
0.5
1
1.5
2
2.5
min
*MSD1 TIC, MS File (D:\DATA\2305\230525I-G 10\I-GEPD1F-D91.D) ES-API, Pos, Scan, Frag: 70, "sca
5000000
4000000
3000000
2000000
1000000
0
0
0.5
1
1.5
2
2.5
min*

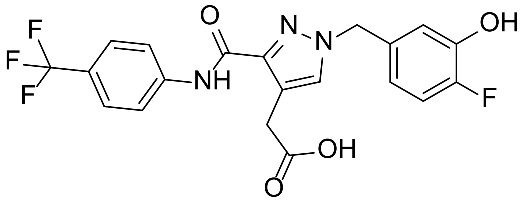
F
F
F
O
NH
N
N
OH
F
OH
O

Compound 4

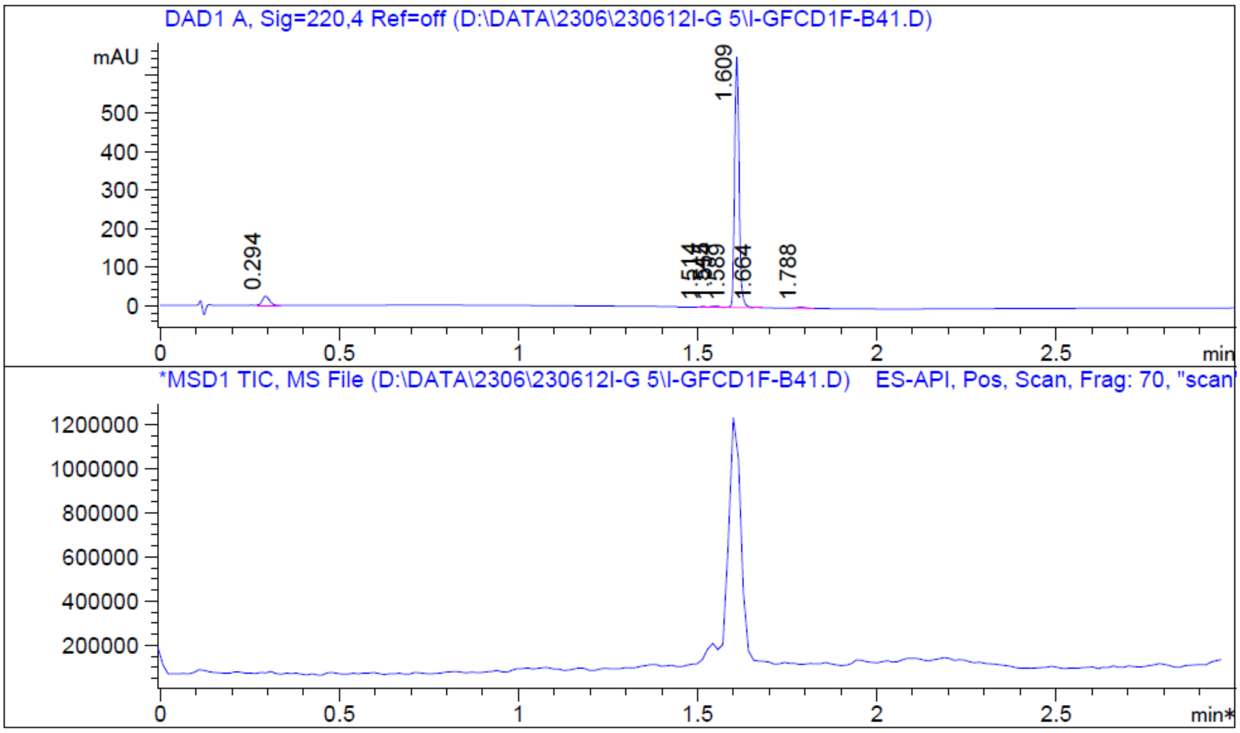
DAD1 A, Sig=220,4 Ref=off (D:\DATA\2306\230612I-G 5\I-GFCD1F-B41.D)
mAU
1.609
0.294
1.664
1.788
*MSD1 TIC, MS File (D:\DATA\2306\230612I-G 5\I-GFCD1F-B41.D) ES-API, Pos, Scan, Frag: 70, "scan"
min

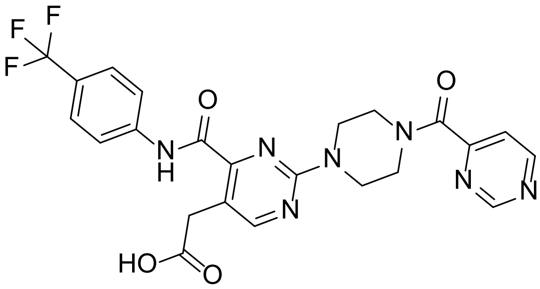

Compound 5

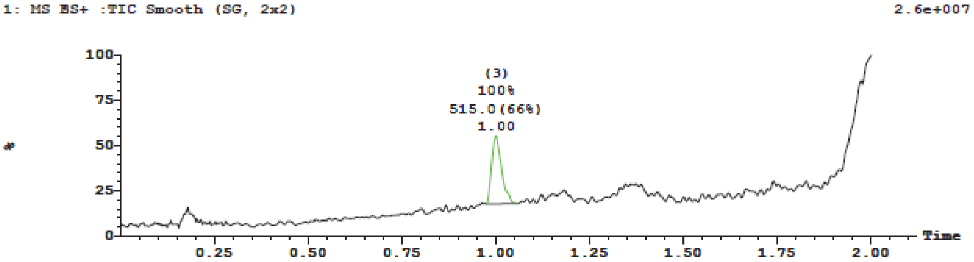

Peak Number	Compound	Time	AreaAbs	Area %Total	Width	Height	Mass Found
3	Found	1.00	3e+005	100.00	0	1e+007	515.0000

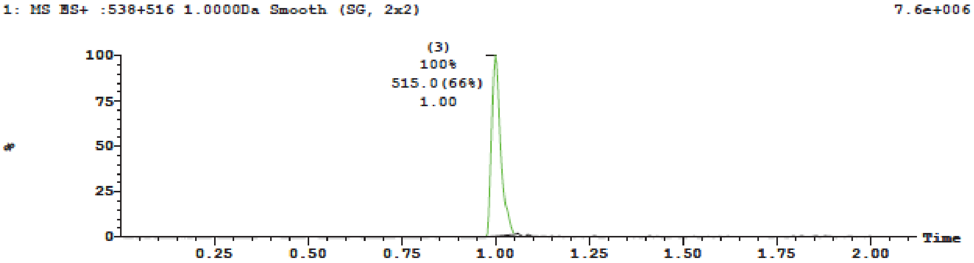

Peak Number	Compound	Time	AreaAbs	Area %Total	Width	Height	Mass Found
3	Found	1.00	2e+005	100.00	0	8e+006	515.0000

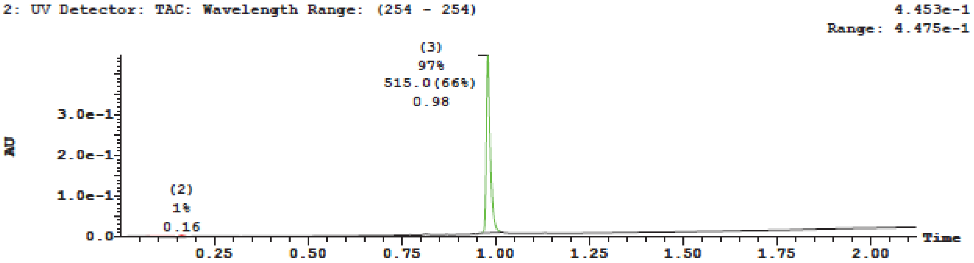

Peak Number	Compound	Time	AreaAbs	Area %Total	Width	Height	Mass Found
1		0.07	8e+001	1.50	0		Not Found
2		0.16	7e+001	1.30	0	4e+003	Not Found
3	Found	0.98	5e+003	97.20	0	4e+005	515.0000

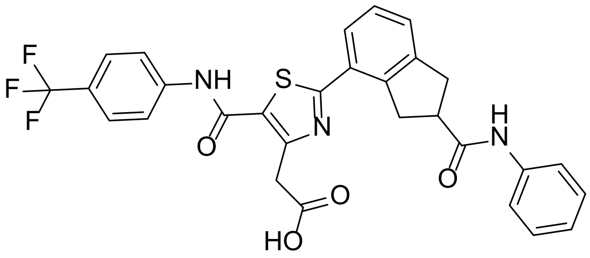

Compound 6

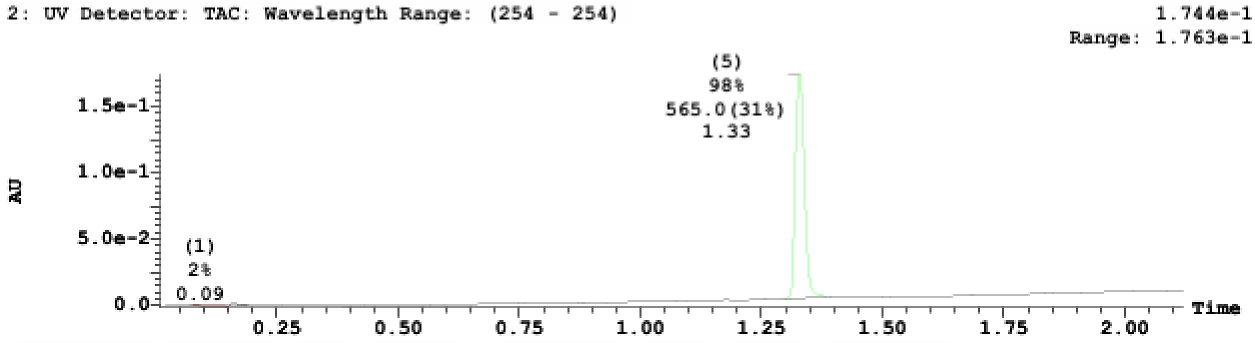

Peak Number	Compound	Time	AreaAbs	Area %Total	Width	Height	Mass Found
1		0.09	7e+001	1.76	0	5e+002	Not Found

Sample Report (continued):

5	Found	1.33	4e+003	98.24	0	2e+005	565.0000

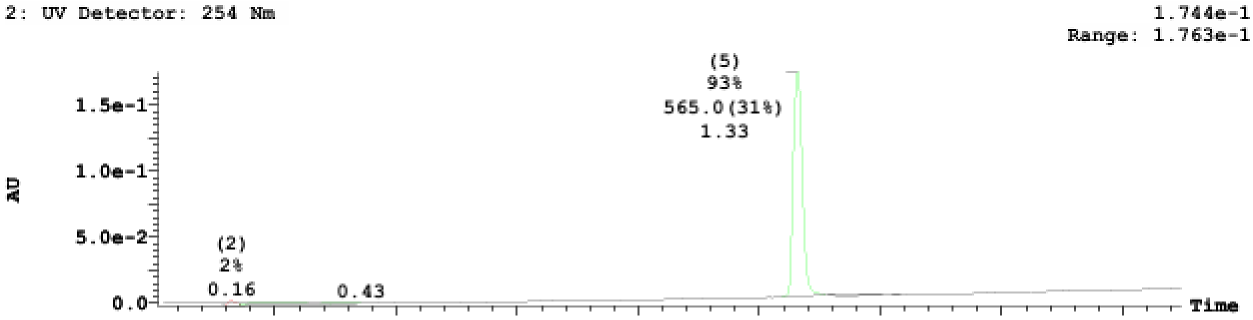

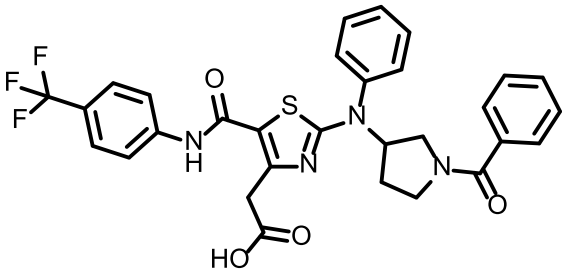

Compound 7

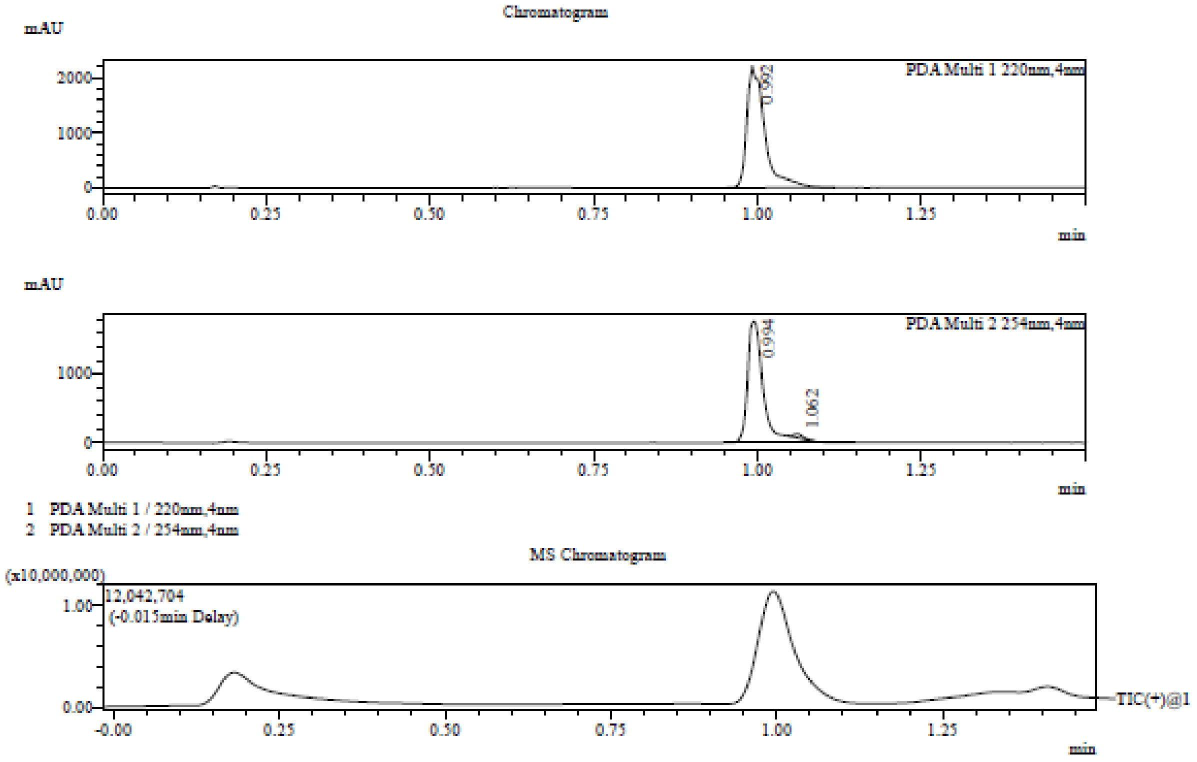

==

Integration Result

==

Peak Table

PDA Ch1 220nm

Peak#	Ret. Time	Height	Height%	USP Width	Area	Area%
1	0.992	2208411	100.000	0.049	4177663	100.000

^{1}H and ^{13}C NMR and HRMS for Fragments **1**-**4**.

Fragment 1: ^{1}H NMR (600 MHz, DMSO) δ 10.29 (s, 1H), 7.79 (d, *J* = 8.5 Hz, 2H), 7.66 (d, *J* = 8.5 Hz, 1H), 2.09 (s, 3H). ^{13}C NMR (151 MHz, DMSO) δ 169.44, 143.32 (q, *J* = 1.3 Hz), 126.47 (q, *J* = 3.9 Hz), 124.87 (q, *J* = 271.3 Hz), 123.44 (q, *J* = 32.1 Hz), 119.22, 24.58.

Fragment 2: ^{1}H NMR (600 MHz, DMSO) δ 8.30 – 8.21 (m, 2H), 7.73 – 7.67 (m, 2H). ^{13}C NMR (151 MHz, DMSO) δ 161.88, 159.32, 149.46, 135.39, 123.18, 122.78, 120.44 (q, *J* = 257.4 Hz). HRMS (ESI): m/z calcd Fragment **2** (C9H5F3N4O3 - H)$^{-}$, 273.0236; found, 273.0221.

Fragment 3: ^{1}H NMR (600 MHz, DMSO) δ 11.25 (s, 1H), 7.83 (s, 1H), 7.27 (s, 1H), 2.36 (s, 3H). ^{13}C NMR (151 MHz, DMSO) δ 151.15, 138.90, 134.63, 131.66, 124.55, 121.79, 117.24, 19.20. HRMS (ESI): m/z calcd Fragment **3** (C8H7ClN2O3S - H)$^{-}$, 244.9788; found, 244.9773.

Fragment 4: ^{1}H NMR (600 MHz, DMSO) δ 13.5 (bs, 1H), 8.60 (d, *J* = 1.5 Hz, 1H), 8.02 (s, 1H), 7.17 (s, 1H), 6.3 (bs, 2H), 2.17 (tt, *J* = 8.4, 5.0 Hz, 1H), 1.16 – 1.03 (m, 2H), 0.96 – 0.84 (m, 2H). ^{13}C NMR (151 MHz, DMSO) δ 165.96, 141.94, 134.45, 133.80, 121.69, 119.19, 112.15, 107.20, 8.55, 7.31. HRMS (ESI): m/z calcd Fragment **4** (C11H11N3O2 + H)$^{+}$, 218.0924; found, 218.0929.

1H NMR of Fragment 1 in DMSO-d6

^{1}H NMR (600 MHz, DMSO) δ 10.29 (s, 1H), 7.79 (d, *J* = 8.5 Hz, 2H), 7.66 (d, *J* = 8.5 Hz, 1H), 2.09 (s, 3H).

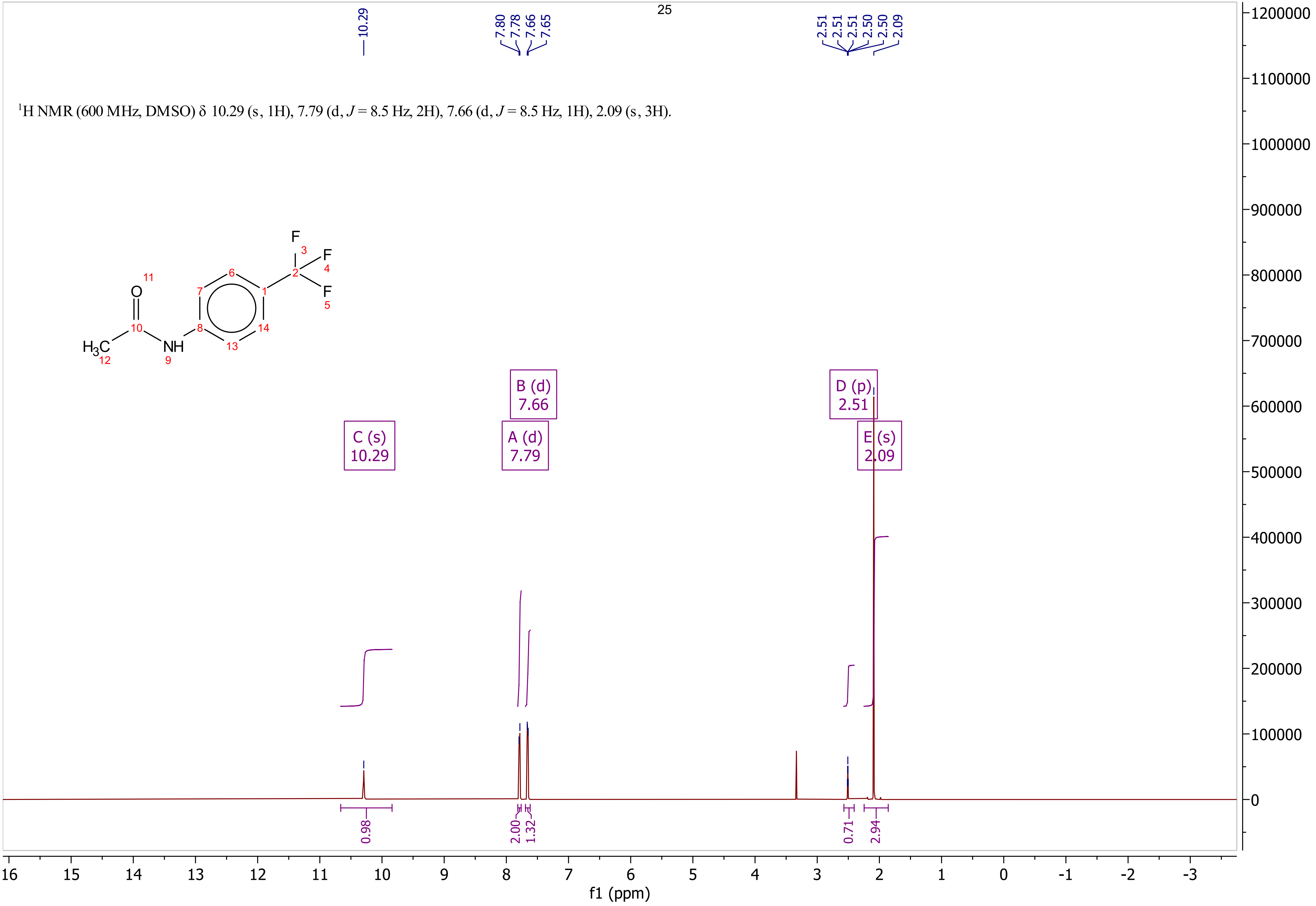

13C NMR of Fragment 1 in DMSO-d6

^{13}C NMR (151 MHz, DMSO) δ 169.44, 143.32 (q, J = 1.3 Hz), 126.47 (q, J = 3.9 Hz), 124.87 (q, J = 271.3 Hz), 123.44 (q, J = 32.1 Hz), 119.22, 24.58.

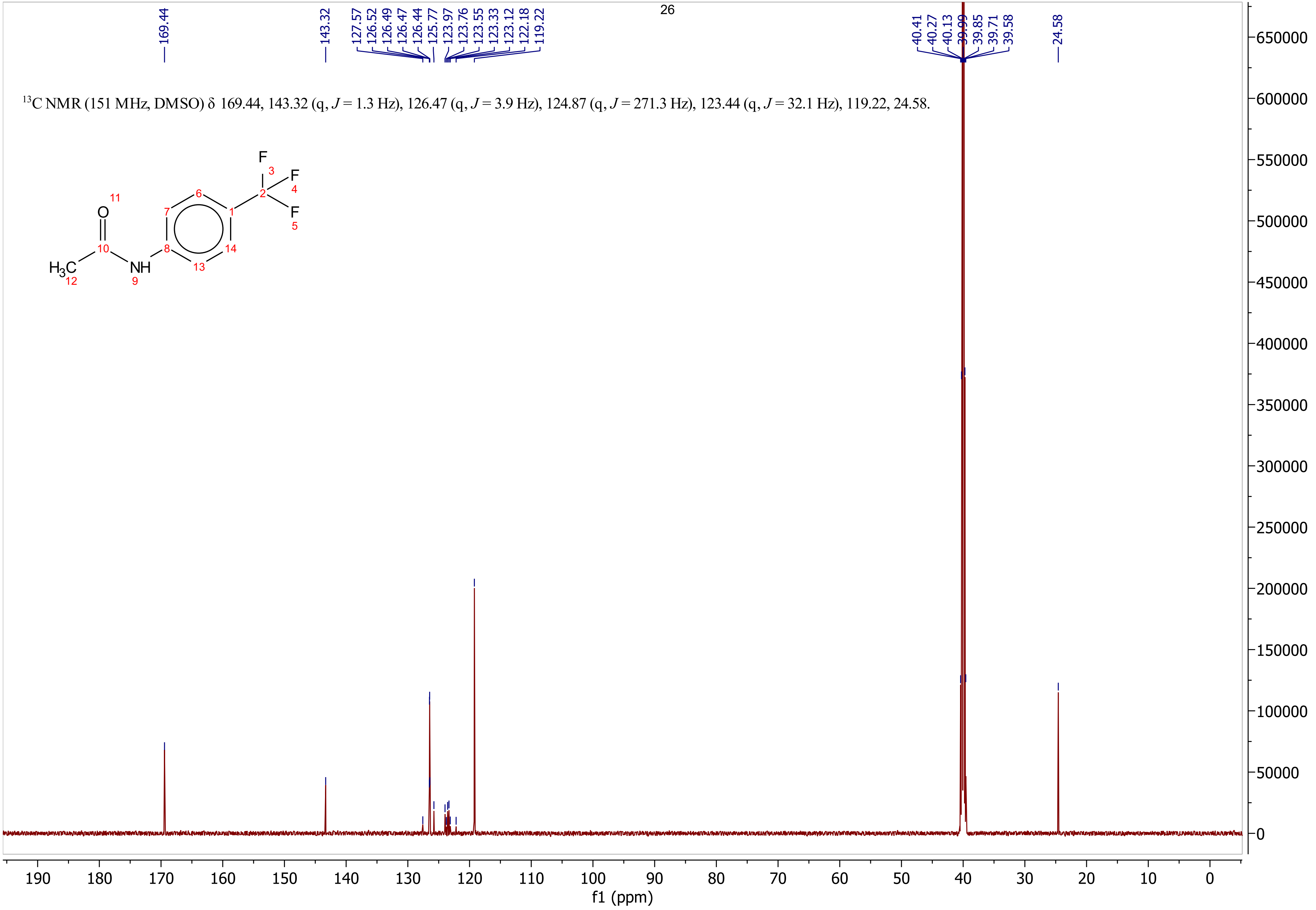

1H NMR of Fragment 2 in DMSO-d6

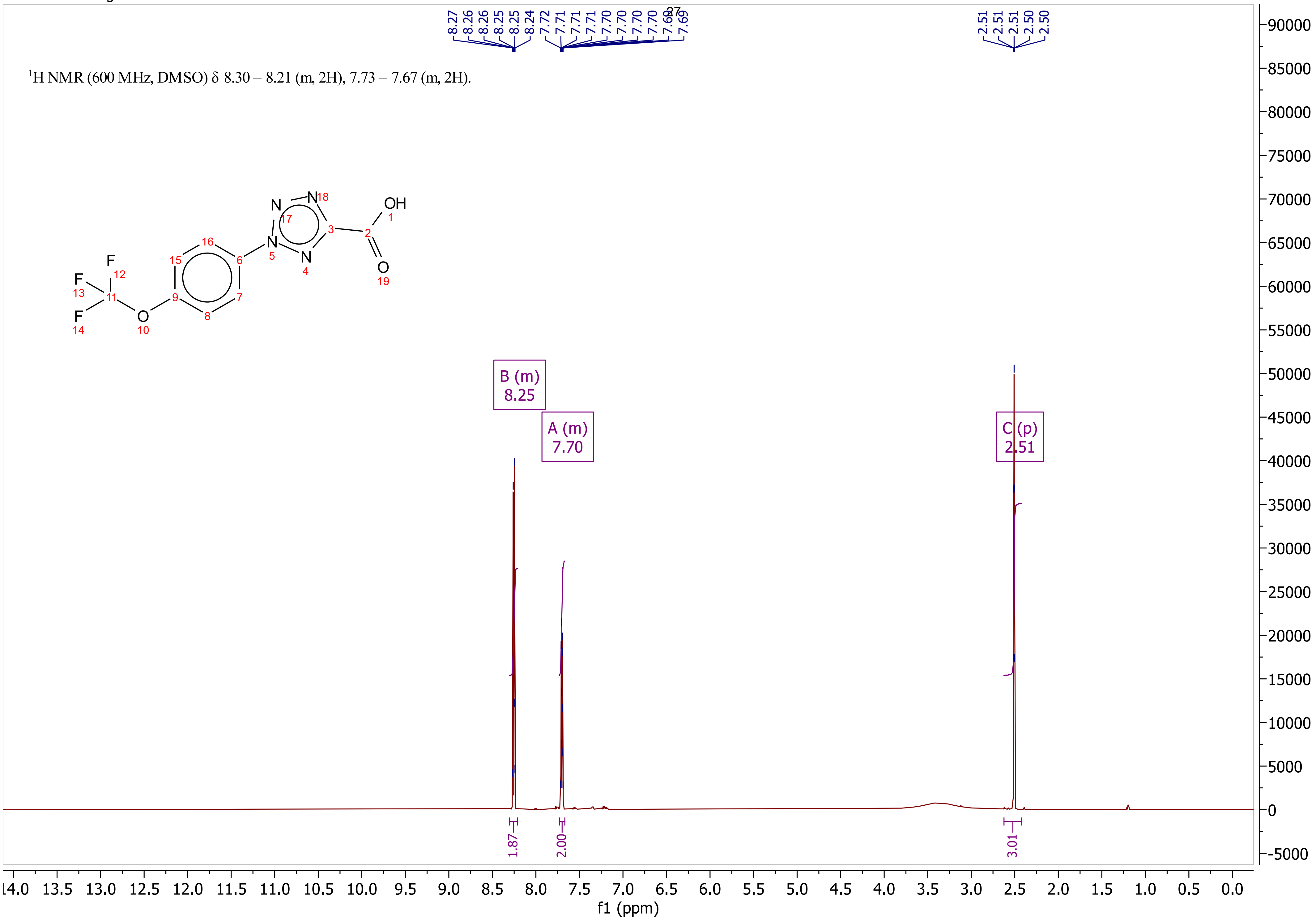

13C NMR of Fragment 2 in DMSO-d6

^{13}C NMR (151 MHz, DMSO) δ 161.88, 159.32, 149.46, 135.39, 123.18, 122.78, 120.44 (q, *J* = 257.4 Hz).

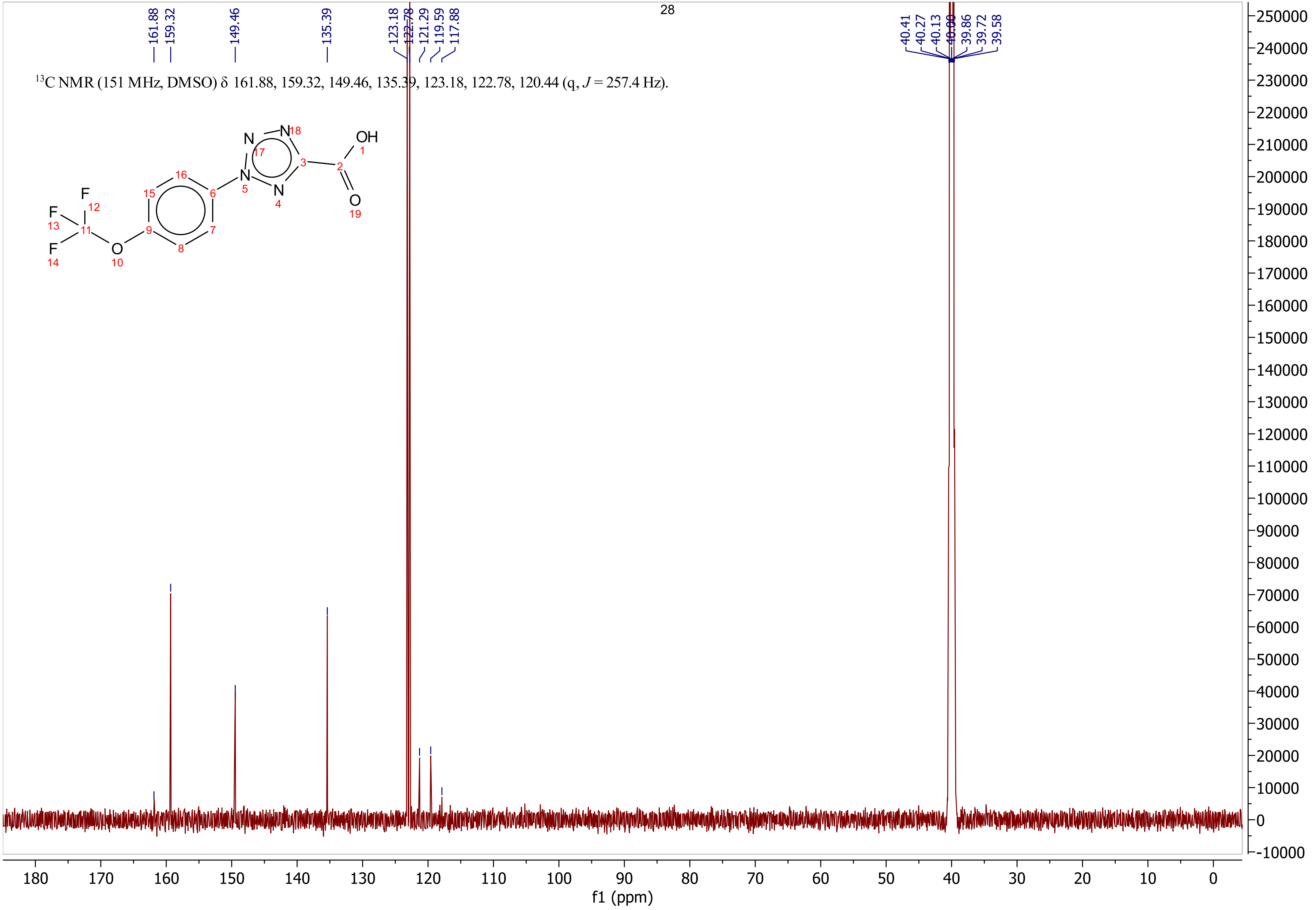

1H NMR of Fragment 3 in DMSO-d6

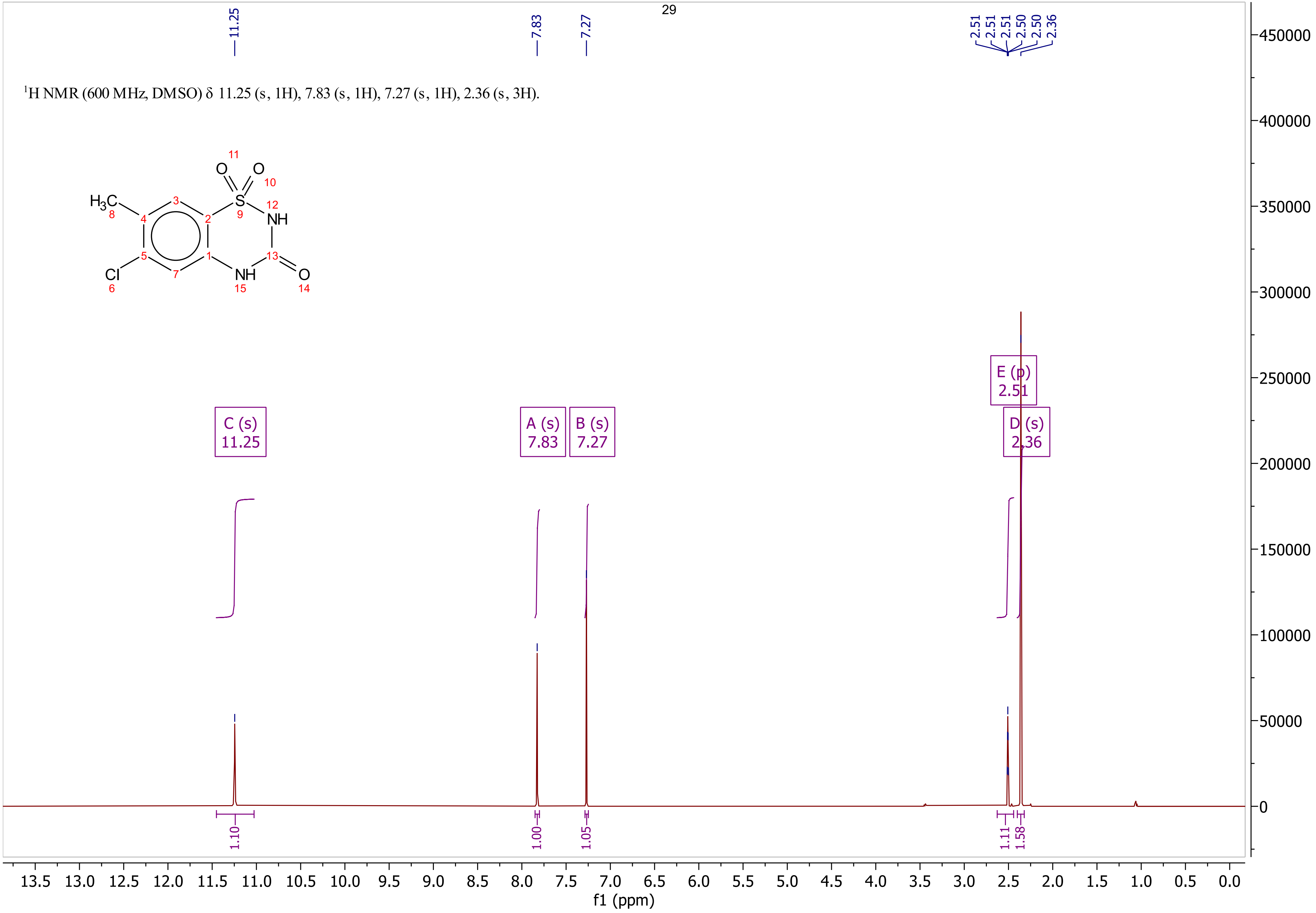

13 NMR of Fragment 3 in DMSO-d6

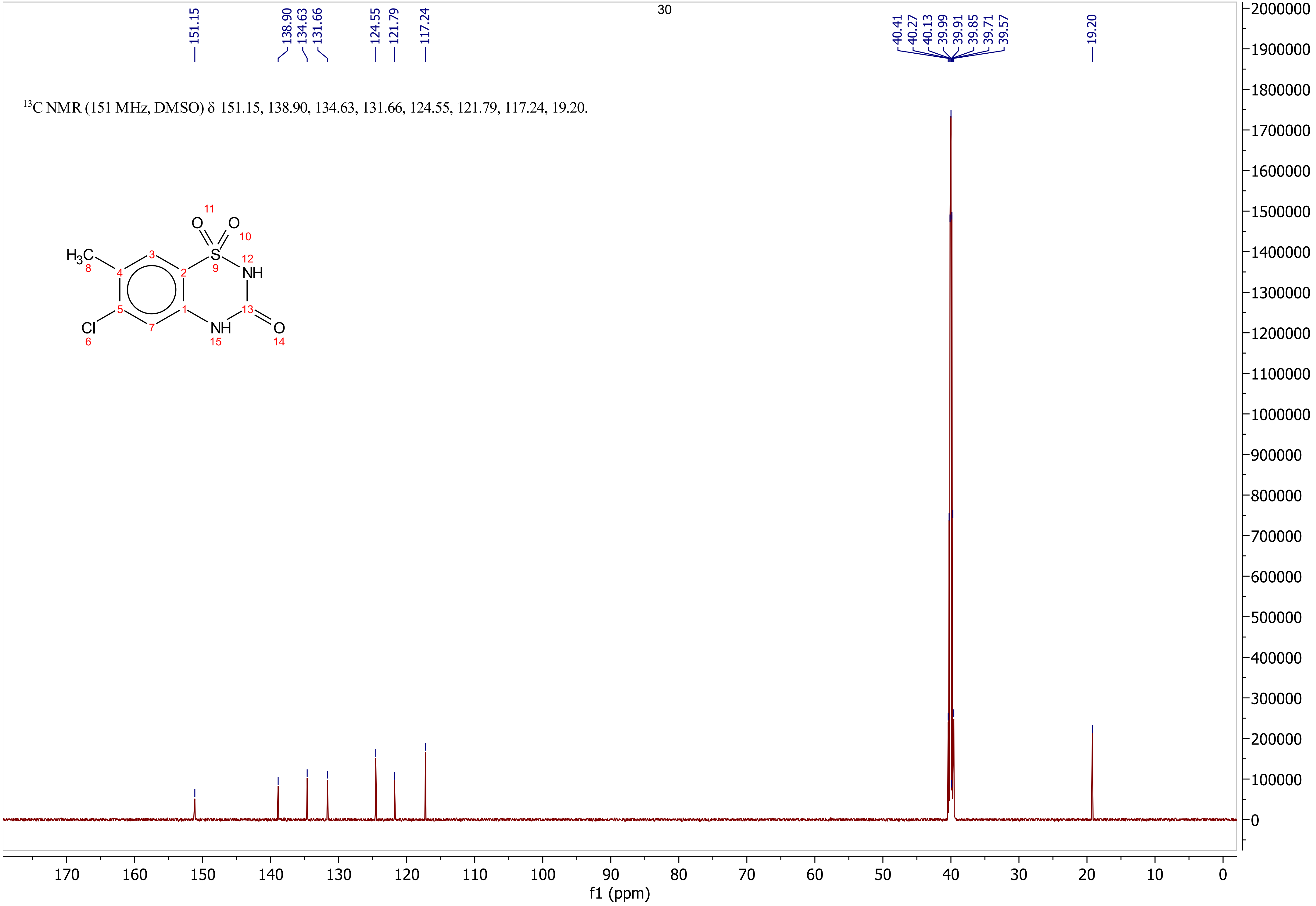

1H NMR of Fragment 4 in DMSO-d6

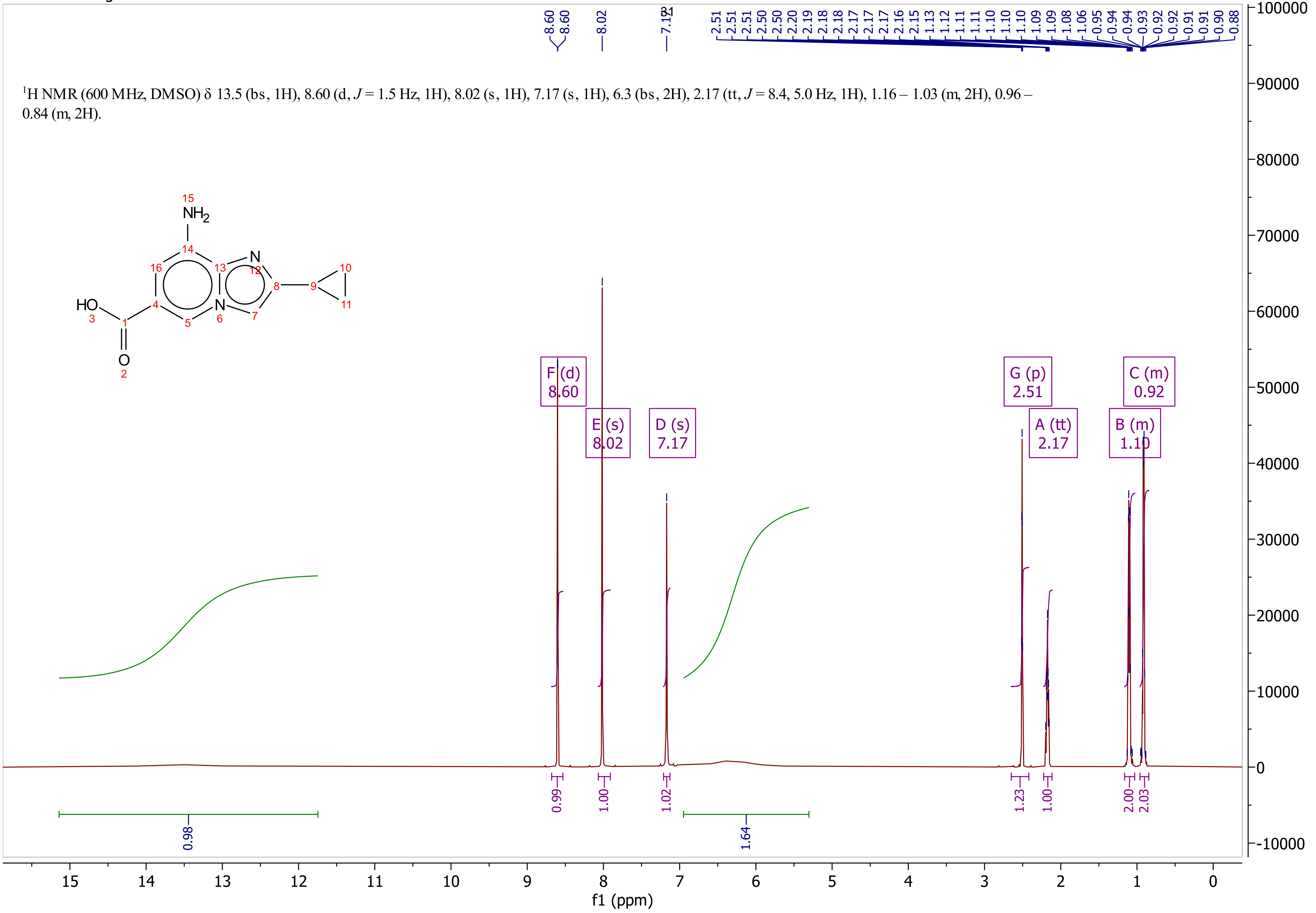

13C NMR of Fragment 4 in DMSO-d6

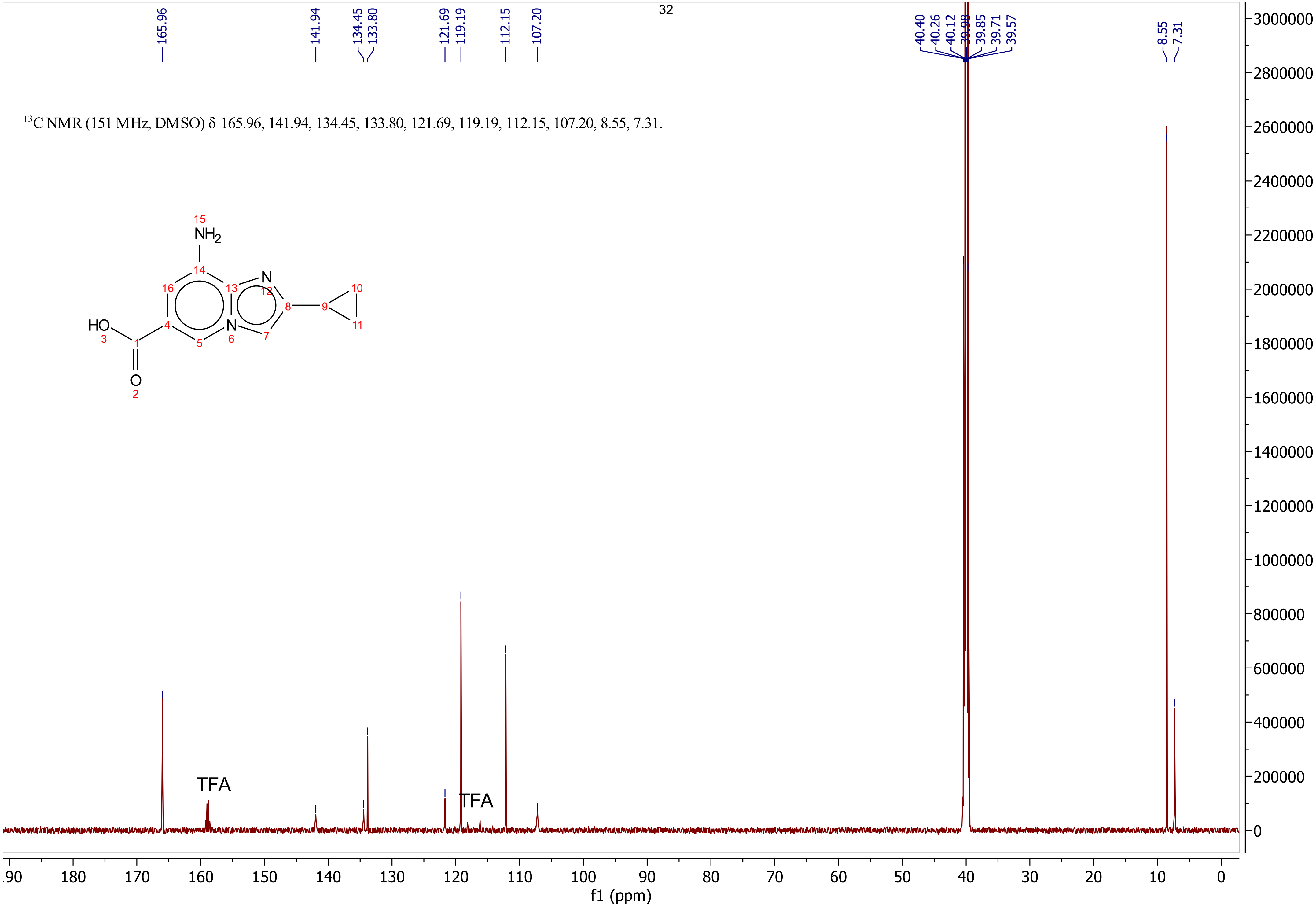

^{13}C NMR (151 MHz, DMSO) δ 165.96, 141.94, 134.45, 133.80, 121.69, 119.19, 112.15, 107.20, 8.55, 7.31.

1H NMR of Compound 1 in DMSO-d6

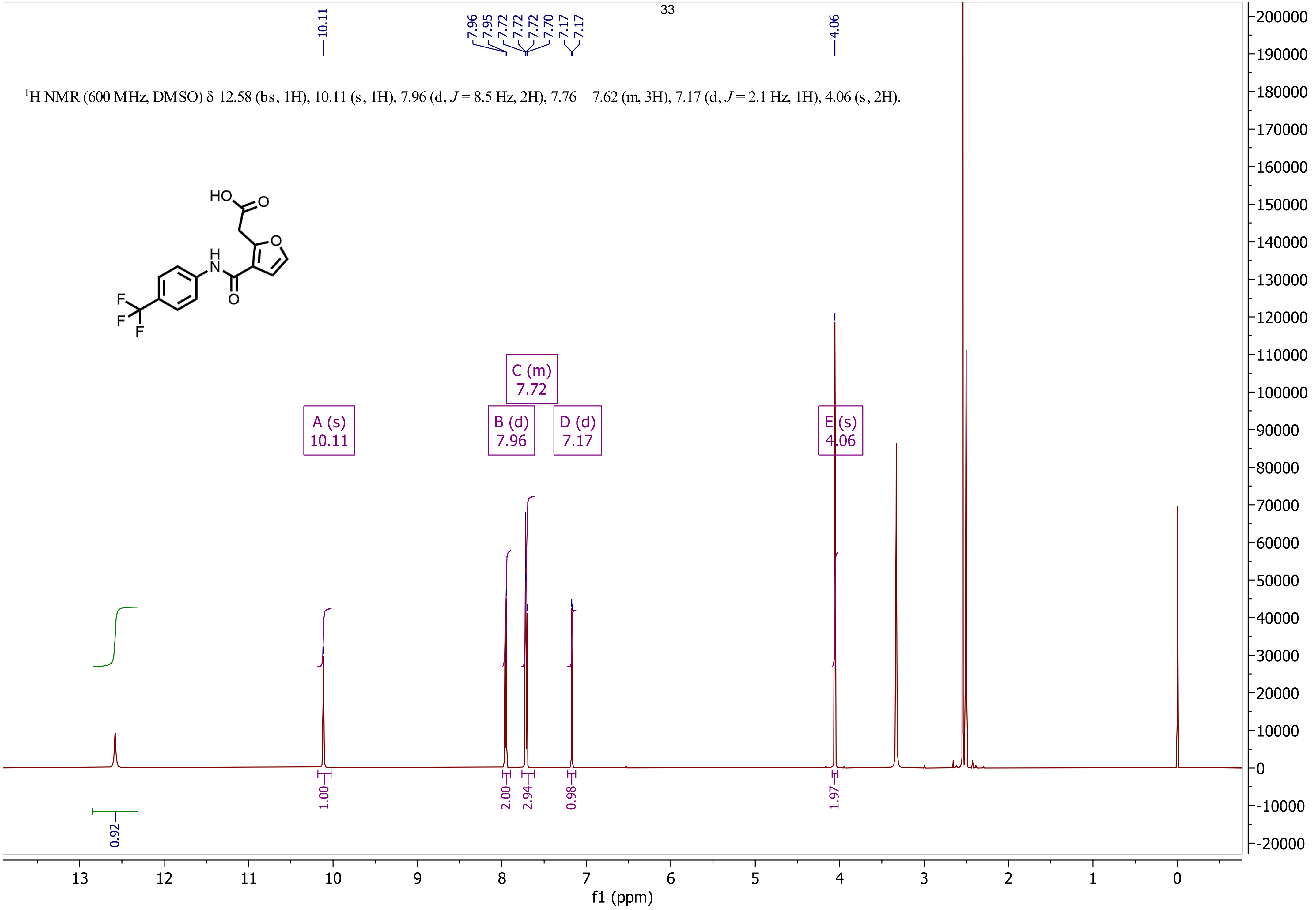

13C NMR of Compound 1 in DMSO-d6

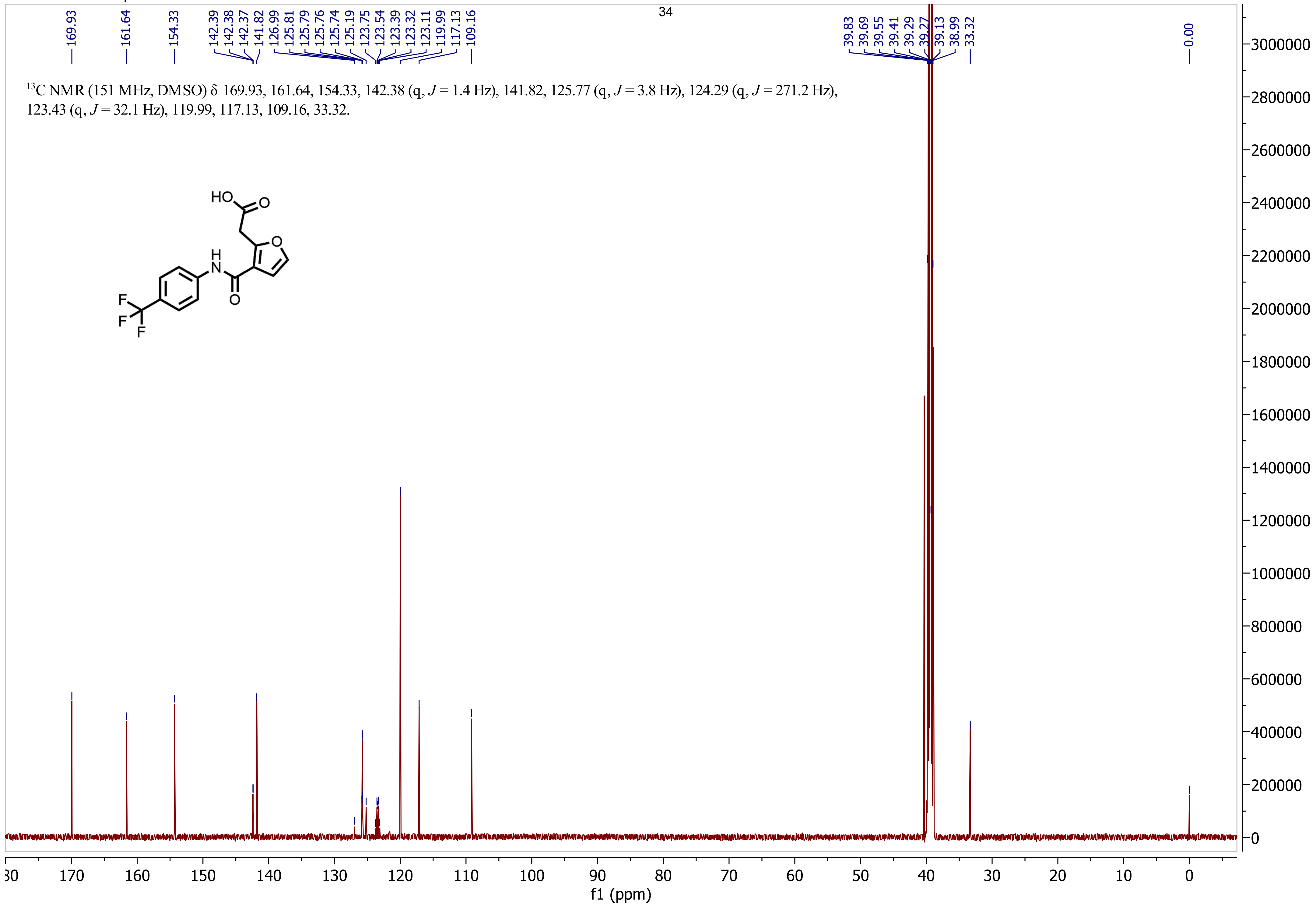

1H NMR of Compound 2 in DMSO-d6

^{1}H NMR (600 MHz, DMSO) δ 12.5 (bs, 1H), 10.33 (s, 1H), 7.94 (d, *J* = 8.6 Hz, 2H), 7.70 (d, *J* = 8.6 Hz, 2H), 7.58 (d, *J* = 5.4 Hz, 1H), 7.52 (d, *J* = 5.4 Hz, 1H), 4.14 (s, 2H).

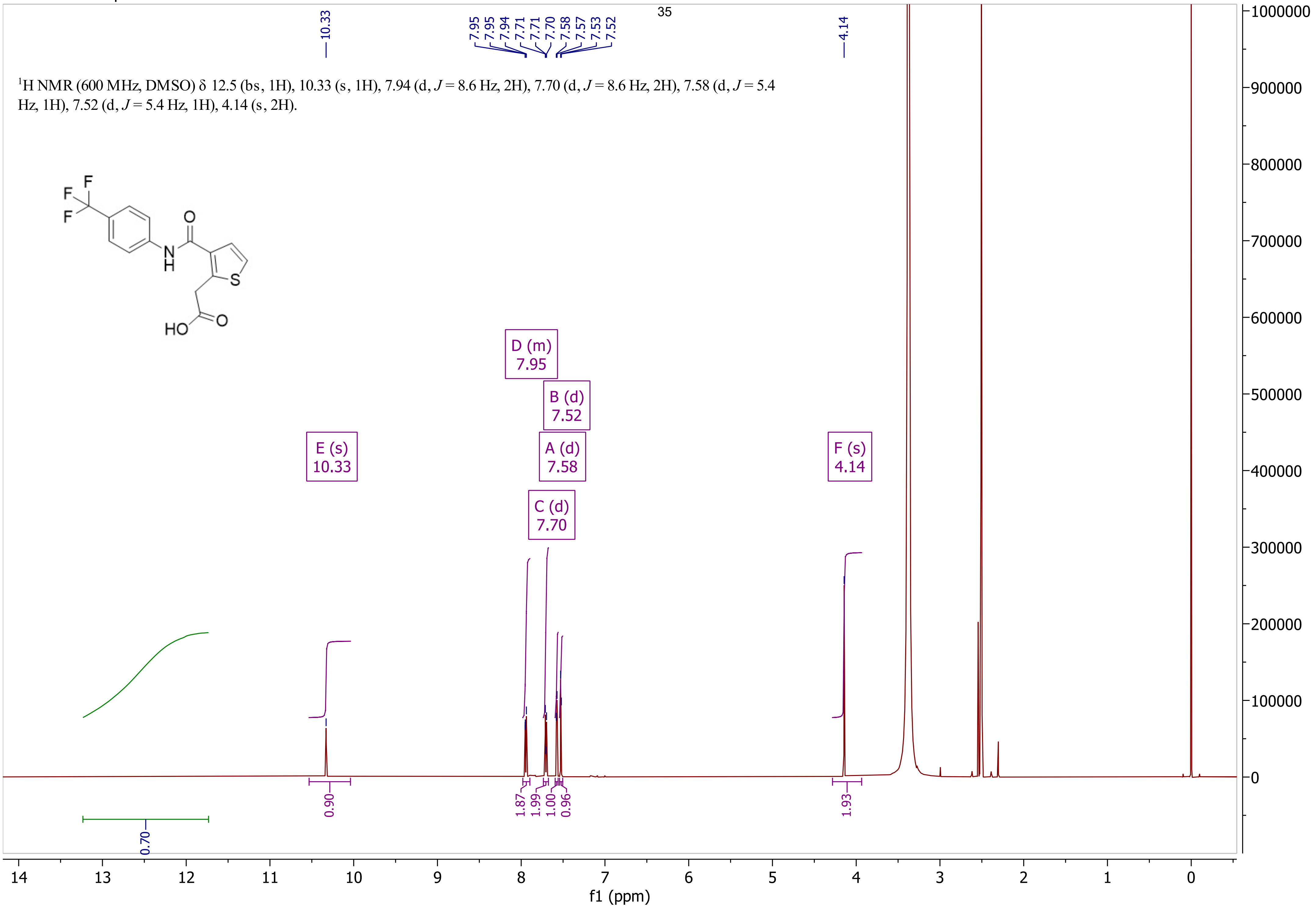

13C NMR of Compound 2 in DMSO-d6

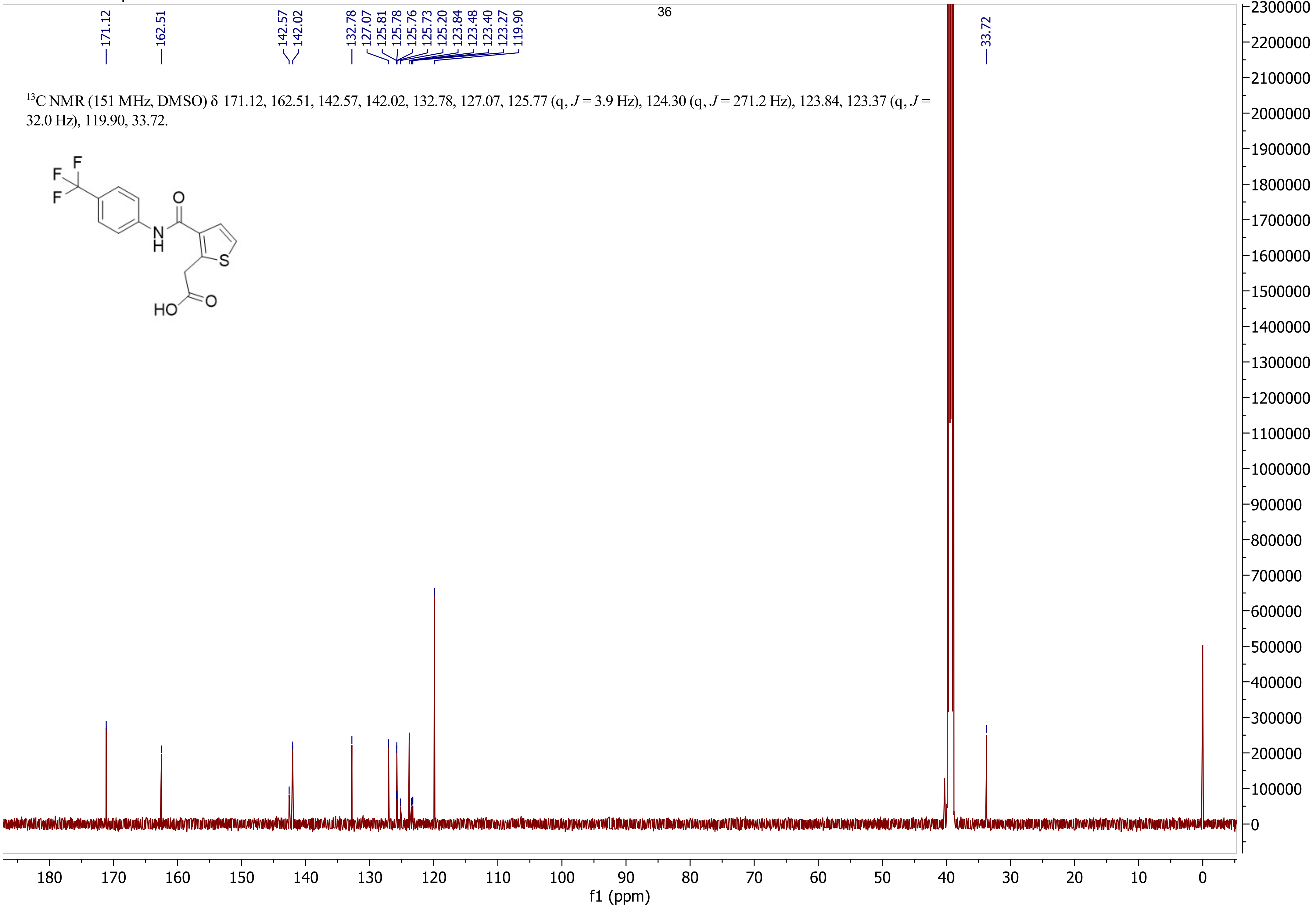

^{13}C NMR (151 MHz, DMSO) δ 171.12, 162.51, 142.57, 142.02, 132.78, 127.07, 125.77 (q, *J* = 3.9 Hz), 124.30 (q, *J* = 271.2 Hz), 123.84, 123.37 (q, *J* = 32.0 Hz), 119.90, 33.72.

1H NMR of Compound 3 in DMSO-d6

^{1}H NMR (600 MHz, DMSO) δ 12.44 (bs, 1H), 10.69 (s, 1H), 10.15 (s, 1H), 7.87 (d, J = 8.5 Hz, 2H), 7.69 (d, J = 8.6 Hz, 2H), 7.63 – 7.56 (m, 2H), 7.41 – 7.33 (m, 2H), 7.05 (tt, J = 7.3, 1.1 Hz, 1H), 3.98 (s, 2H).

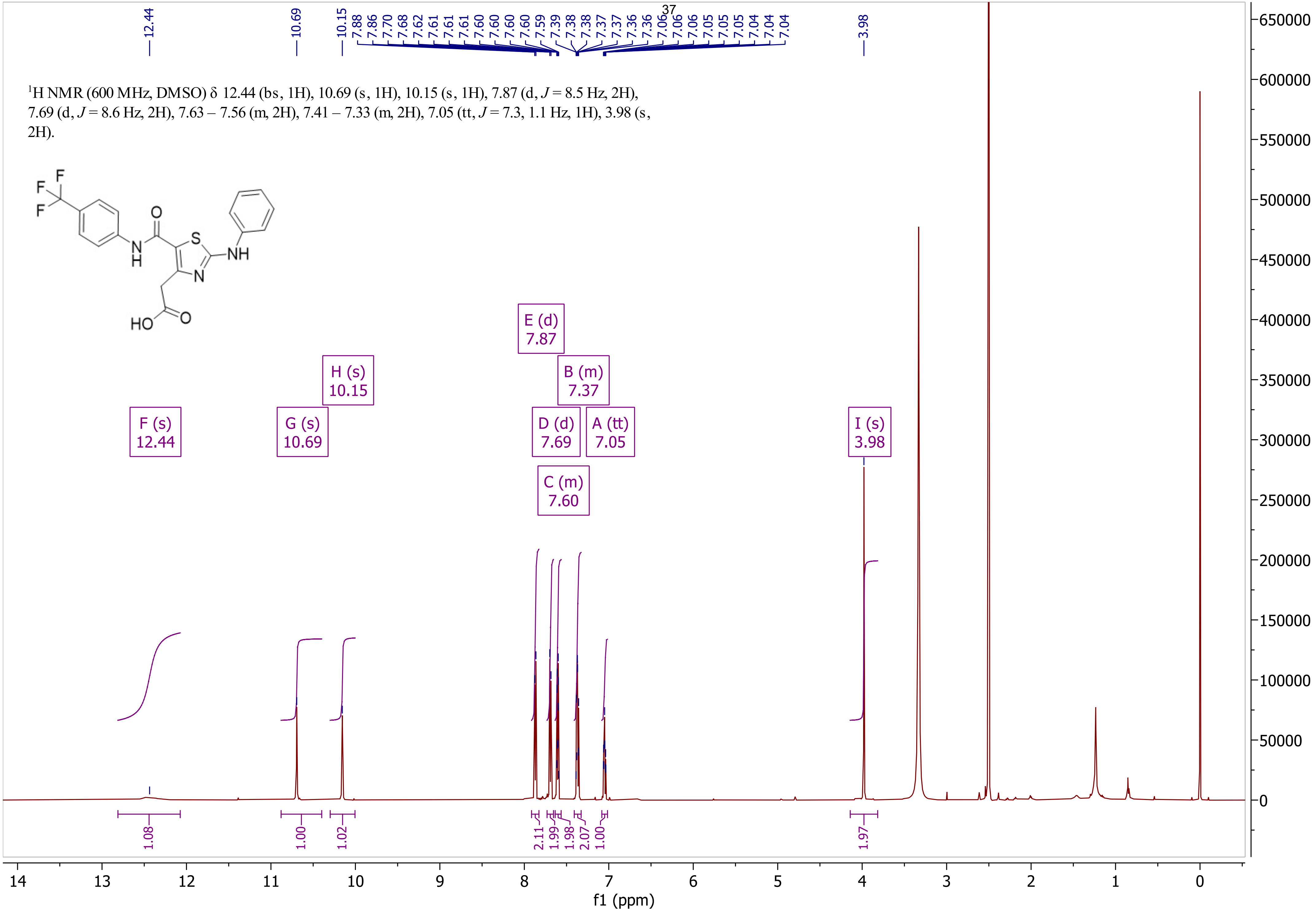

13C NMR of Compound 3 in DMSO-d6

^{13}C NMR (151 MHz, DMSO) δ 171.11, 163.47, 160.23, 152.40, 142.48, 140.14, 129.09, 125.73 (q, *J* = 3.9 Hz), 124.28 (q, *J* = 271.8 Hz), 122.44, 120.17, 117.79, 113.97, 36.81.

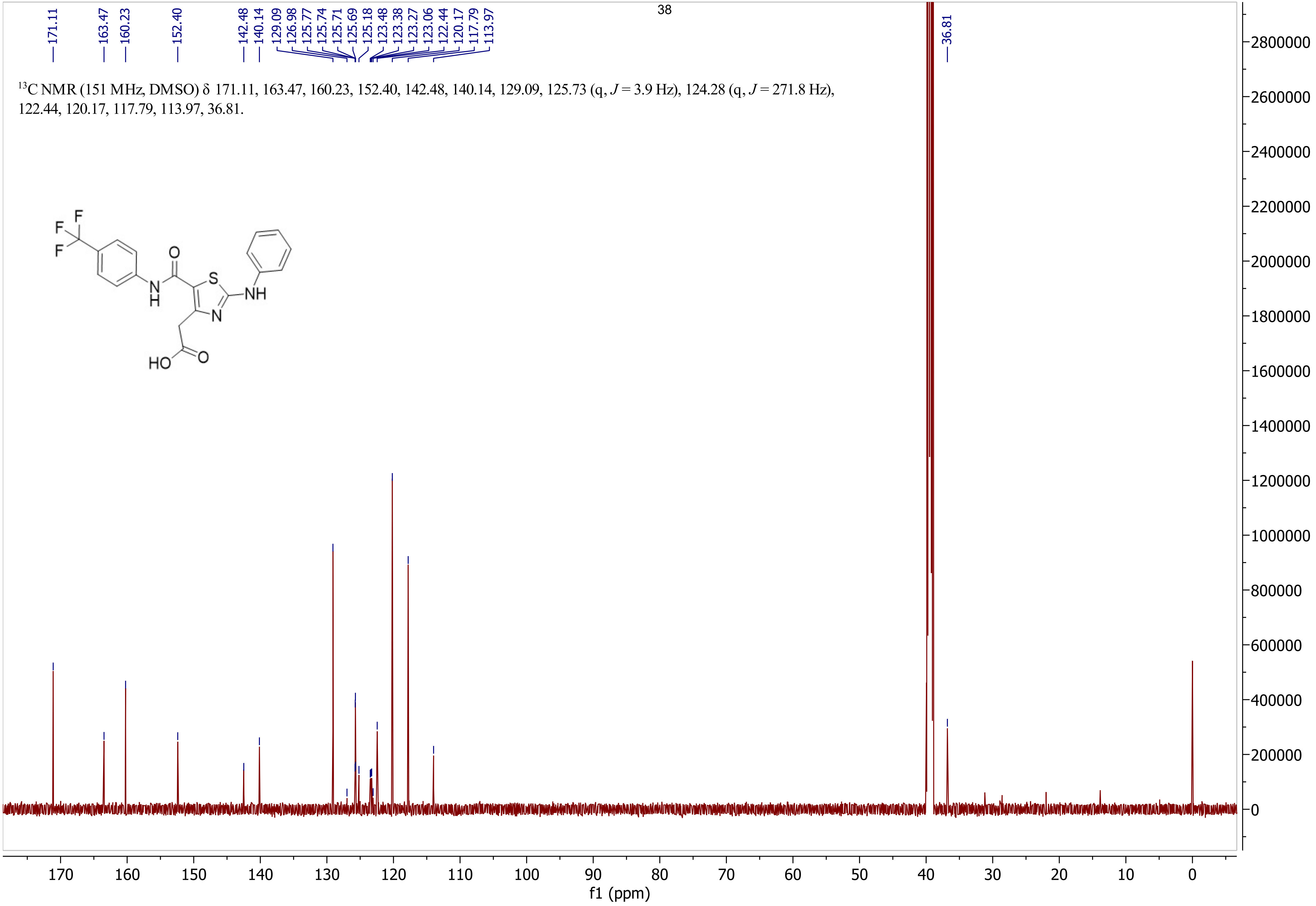

1H NMR of Compound 4 in DMSO-d6

^{1}H NMR (600 MHz, DMSO) δ 12.18 (s, 1H), 10.40 (s, 1H), 9.95 (s, 1H), 8.03 (d, *J* = 8.3 Hz, 2H), 7.85 (s, 1H), 7.67 (d, *J* = 8.3 Hz, 2H), 7.13 (dd, *J* = 11.3, 8.3 Hz, 1H), 6.85 (dd, *J* = 8.5, 2.2 Hz, 1H), 6.74 (ddd, *J* = 8.4, 4.2, 2.2 Hz, 1H), 5.34 (s, 2H), 3.74 (s, 2H).

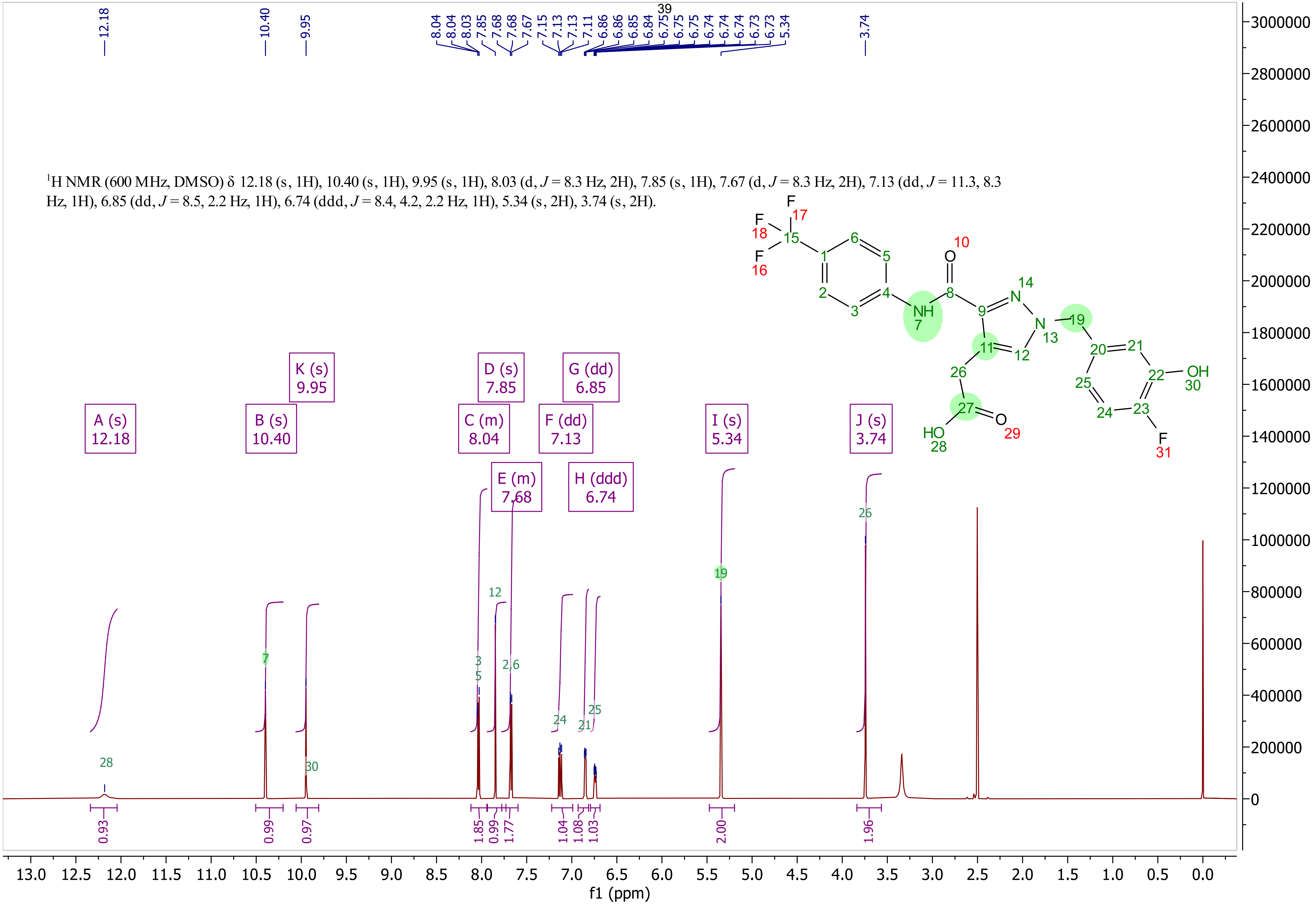

13C NMR of Compound 4 in DMSO-d6

^{13}C NMR (151 MHz, DMSO) δ 172.16, 161.18, 150.41 (d, J = 241.0 Hz), 144.84 (d, J = 12.4 Hz), 142.73, 142.32 (q, J = 1.5 Hz), 133.16 (d, J = 3.4 Hz), 132.53, 125.66 (q, J = 3.8 Hz), 124.32 (q, J = 271.5 Hz), 123.19 (q, J = 31.8 Hz), 119.93, 118.48 (d, J = 6.8 Hz), 116.84, 116.77 (d, J = 3.2 Hz), 116.16 (d, J = 18.4 Hz), 54.52, 29.45.

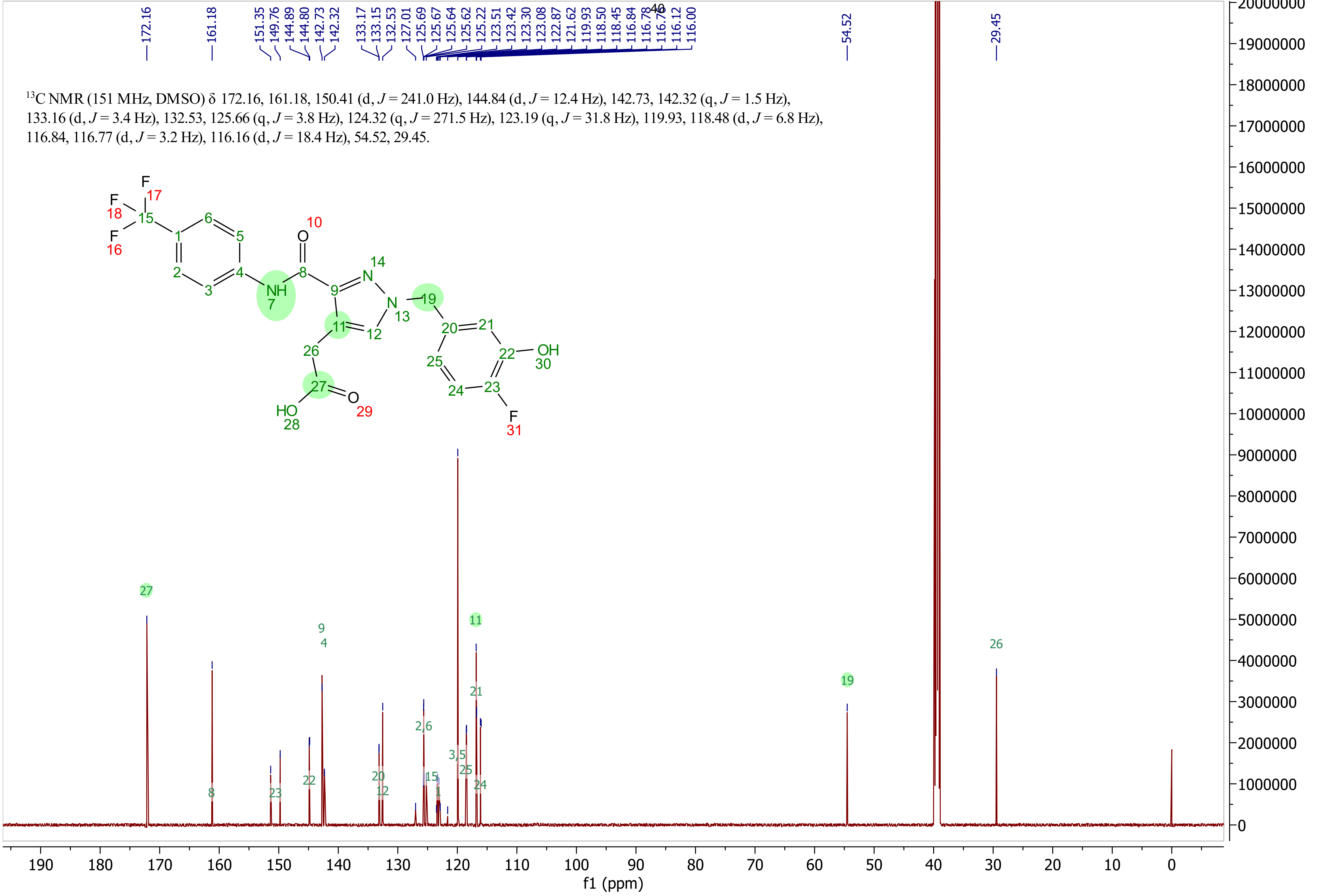

COSY of Compound 4 in DMSO-d6

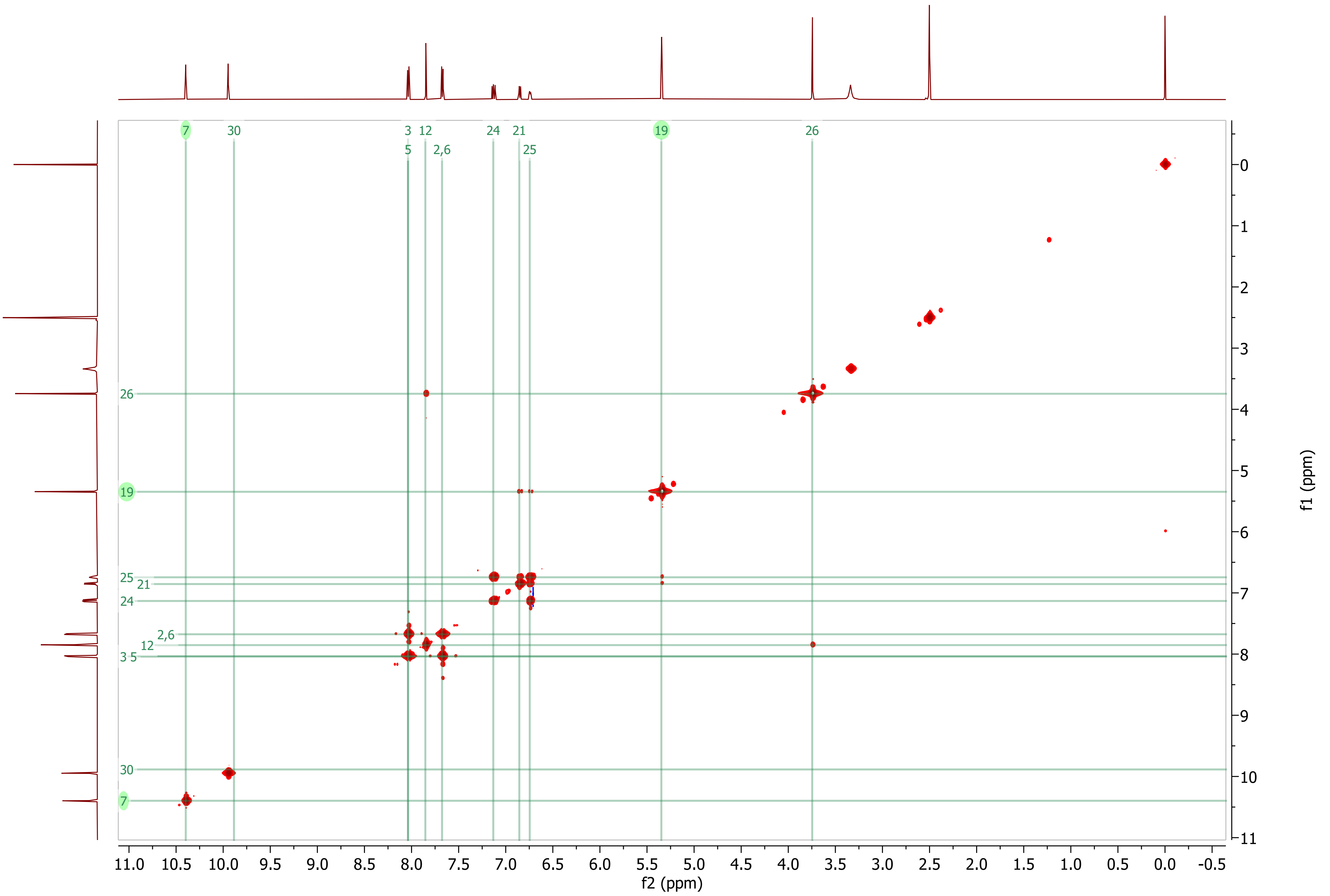

NOESY of Compound 4 in DMSO-d6

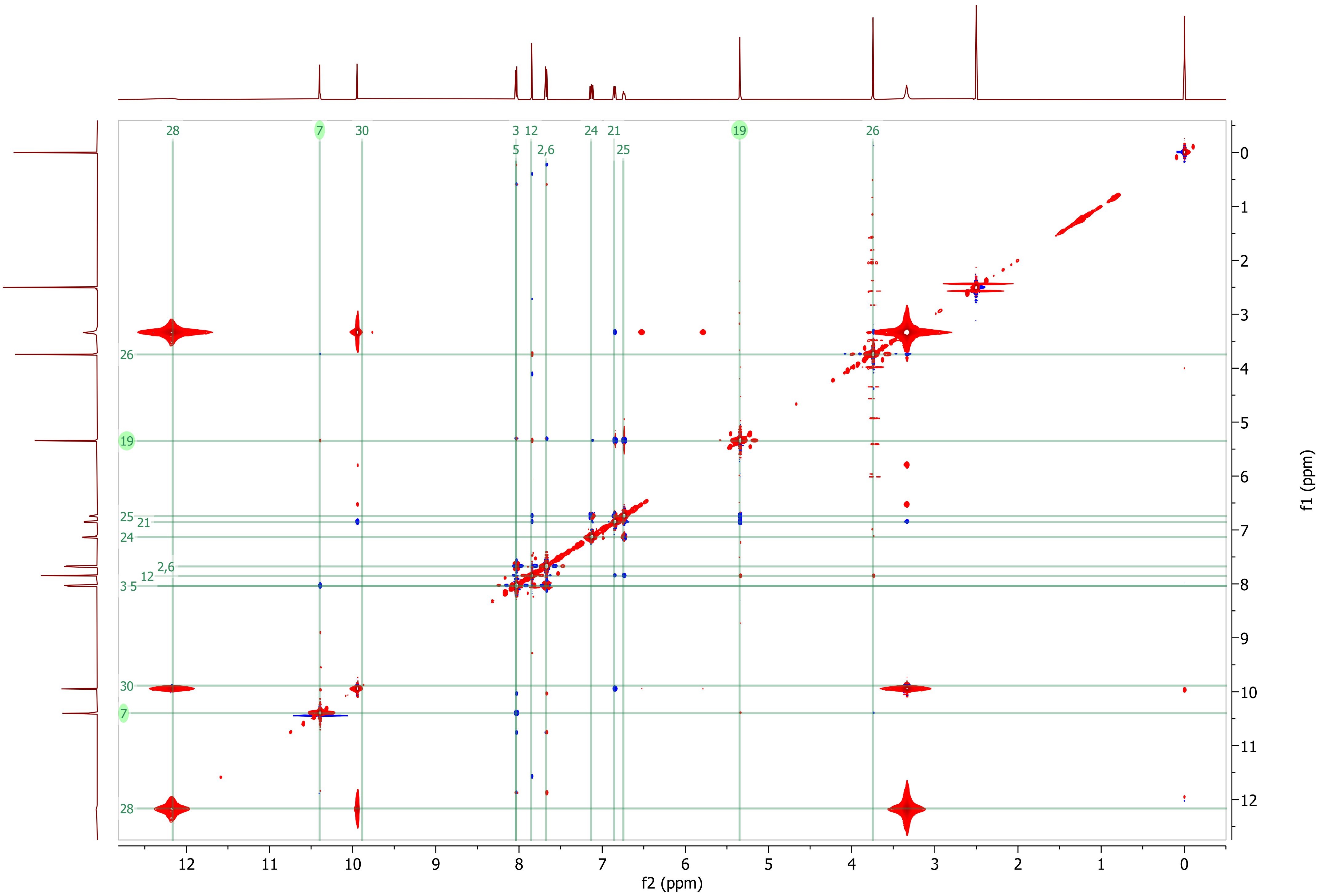

HSQC of Compound 4 in DMSO-d6

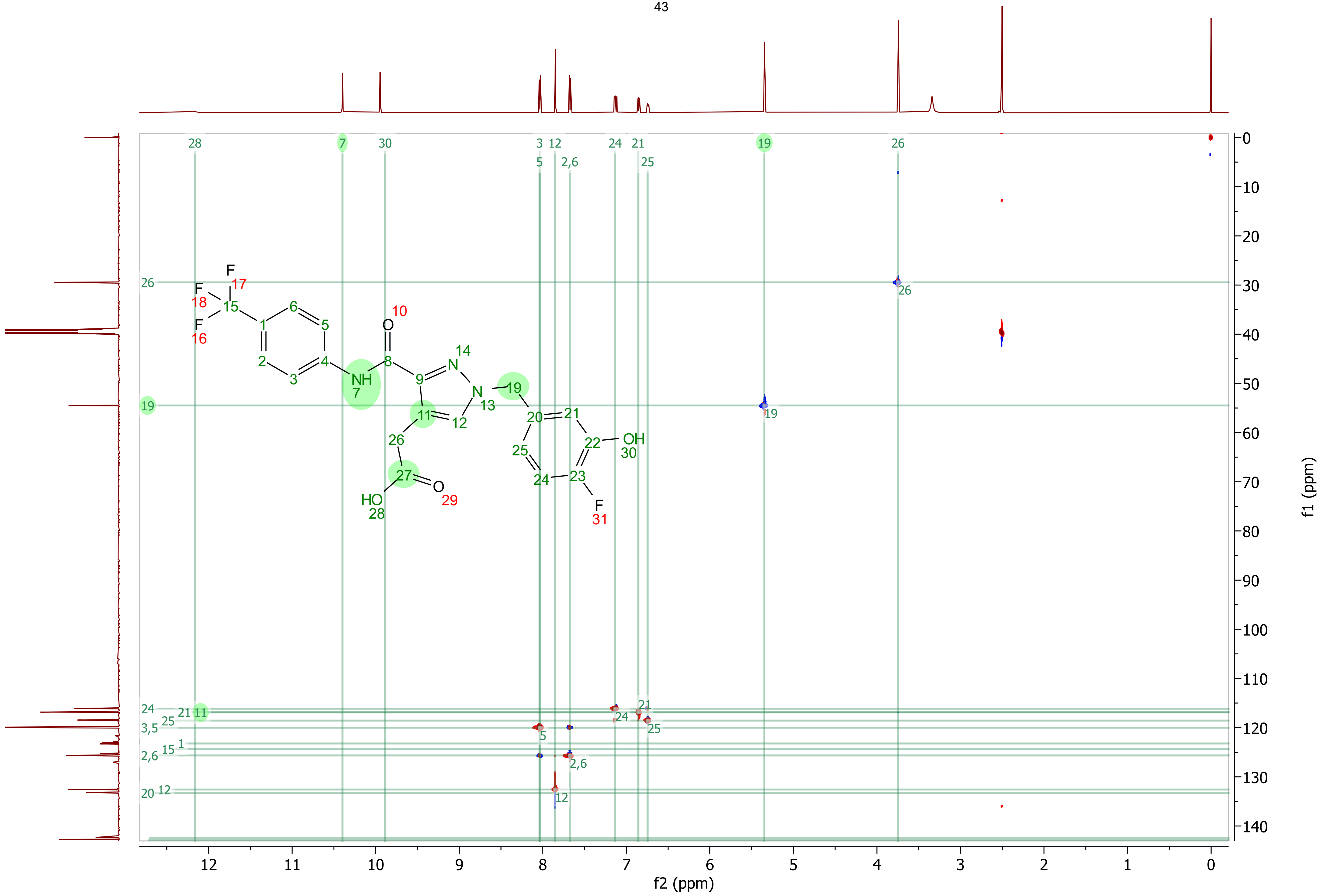

HMBC of Compound 4 in DMSO-d6

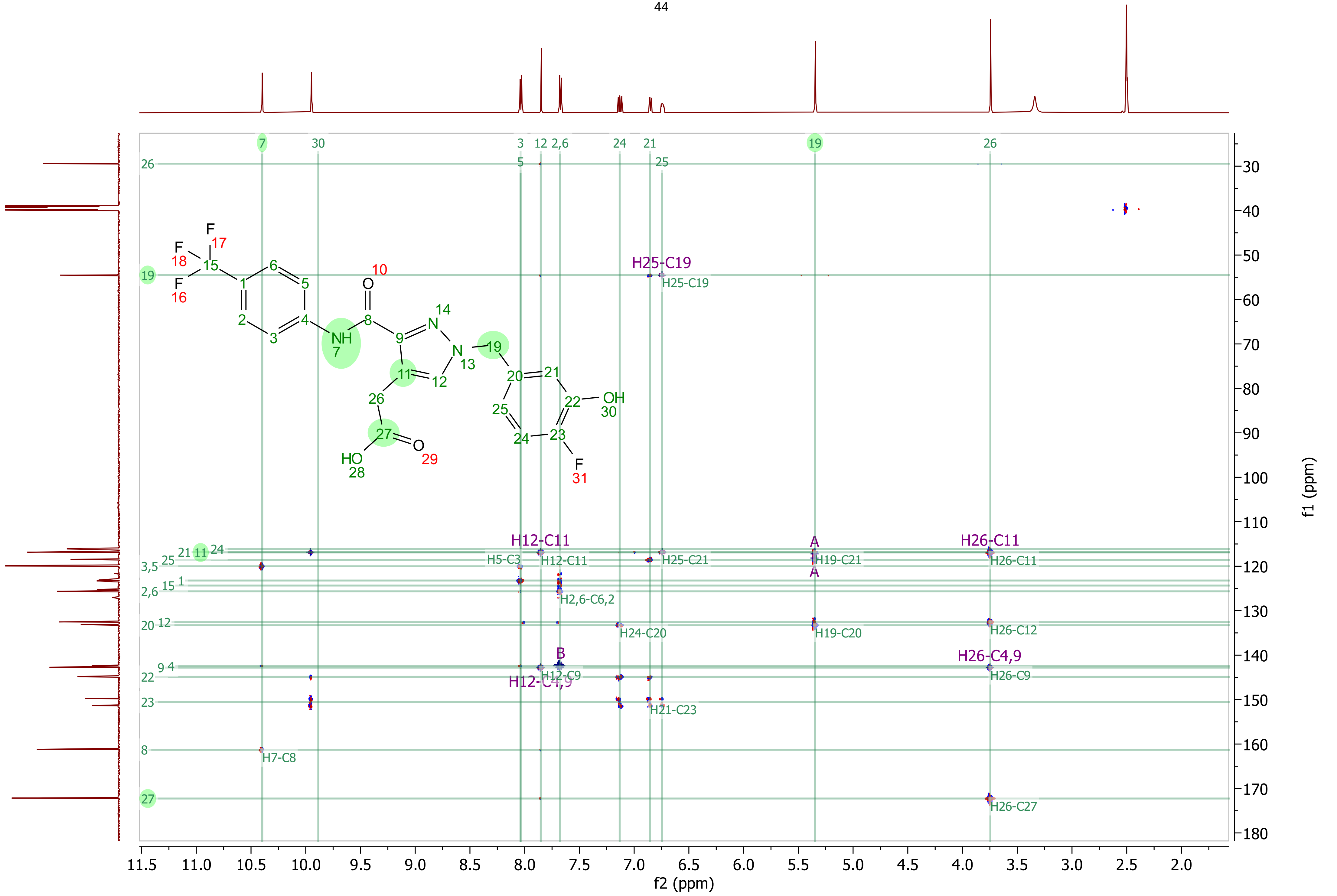

HMBC_N15 of Compound 4 in DMSO-d6

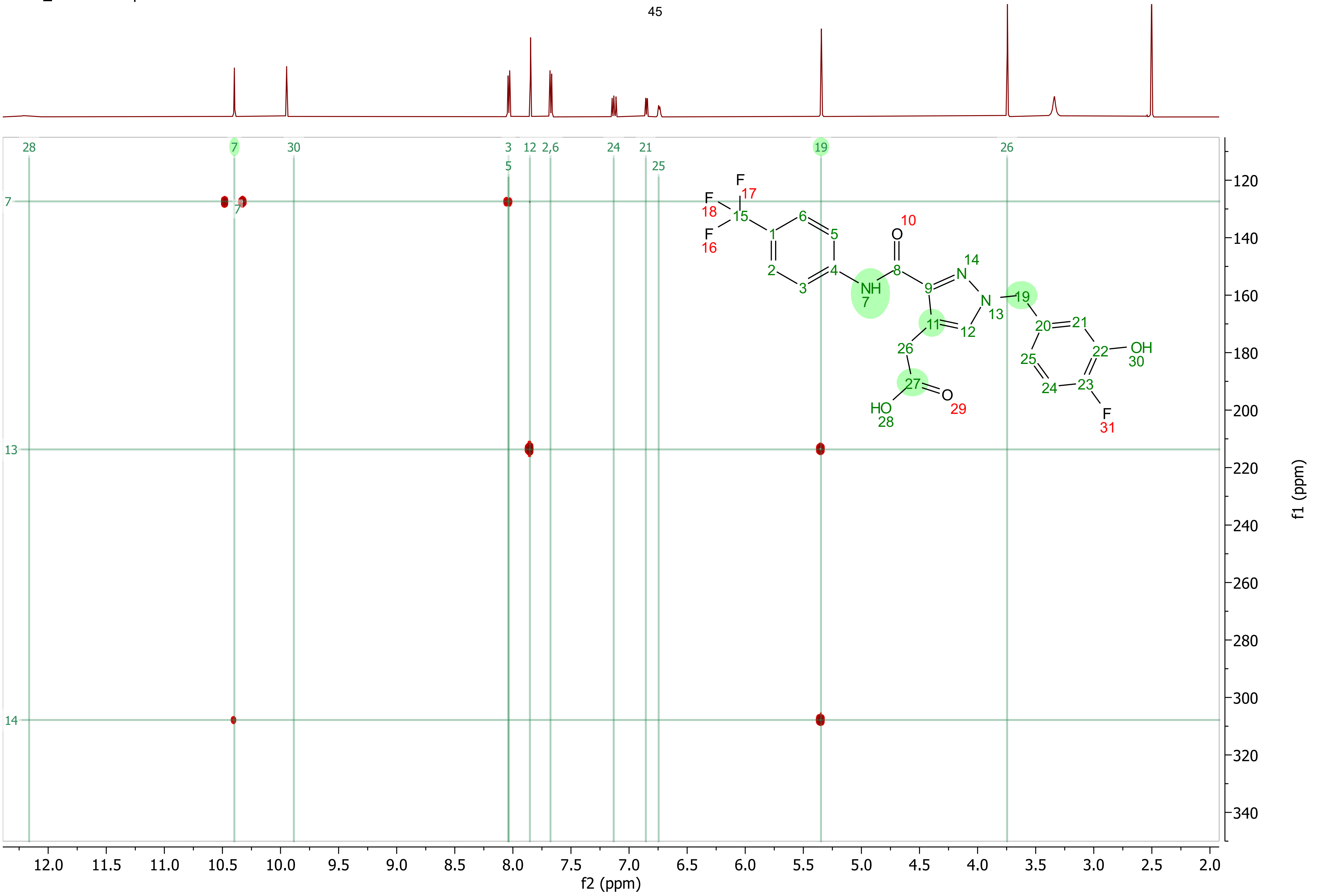

1H NMR of Compound 5 in DMSO-d6

^{1}H NMR (600 MHz, DMSO) δ 10.72 (bs, 1H), 9.29 (d, J = 1.5 Hz, 1H), 9.01 (d, J = 5.1 Hz, 1H), 8.49 (s, 1H), 7.99 (d, J = 8.4 Hz, 2H), 7.77 – 7.71 (m, 3H), 4.01– 3.98 (m, 2H), 3.92 – 3.85 (m, 2H), 3.84 – 3.76 (m, 4H), 3.54 – 3.49 (m, 2H).

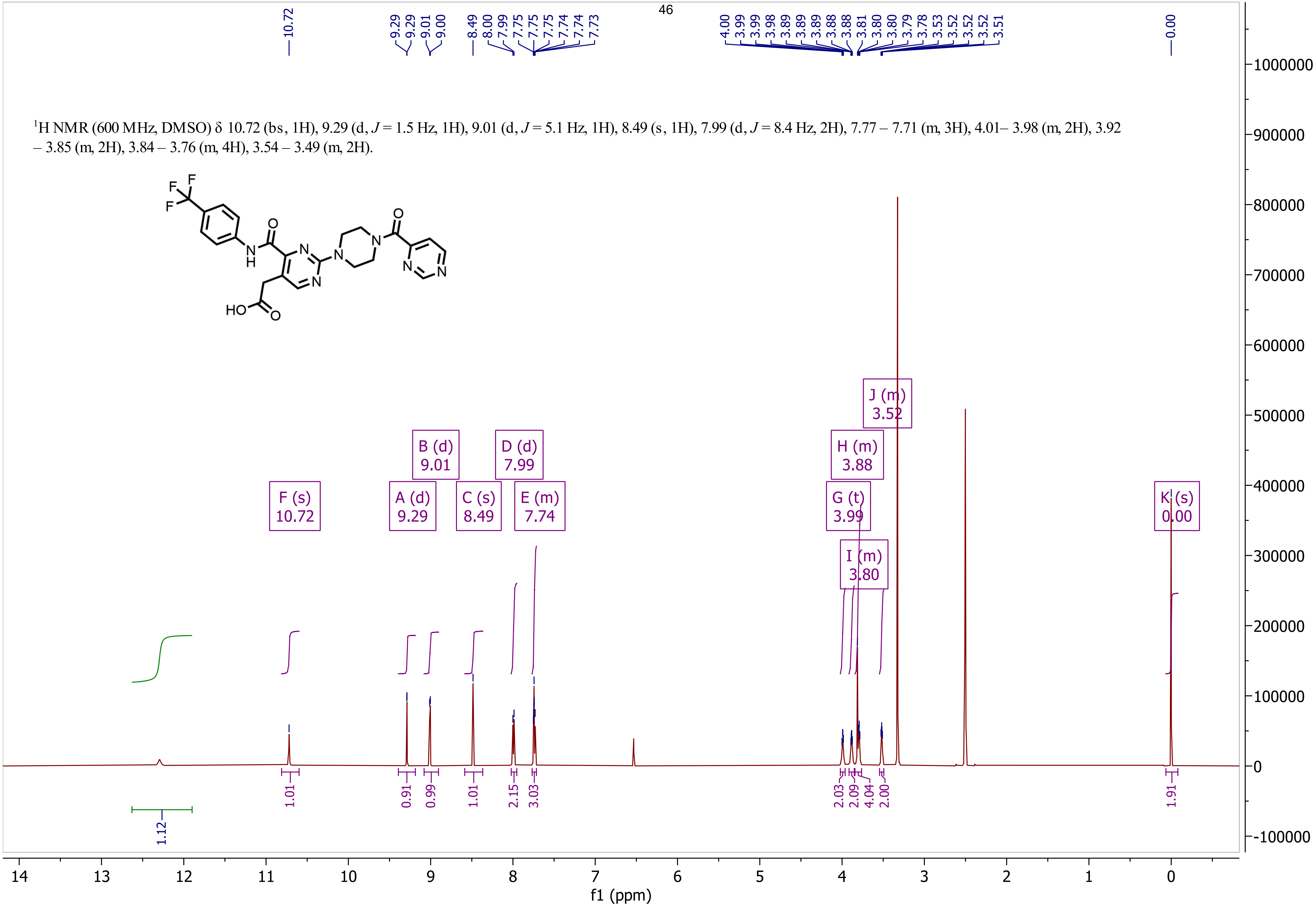

13C NMR of Compound 5 in DMSO-d6

^{13}C NMR (151 MHz, DMSO) δ 172.20, 164.90, 163.96, 162.62, 160.68, 159.61, 158.74, 157.83, 155.76, 141.58, 125.83 (q, J = 3.8 Hz), 124.21 (q, J = 271.2 Hz), 124.02 (q, J = 32.1 Hz), 120.30, 119.98, 115.68, 46.0, 43.69, 43.04, 41.29, 34.16.

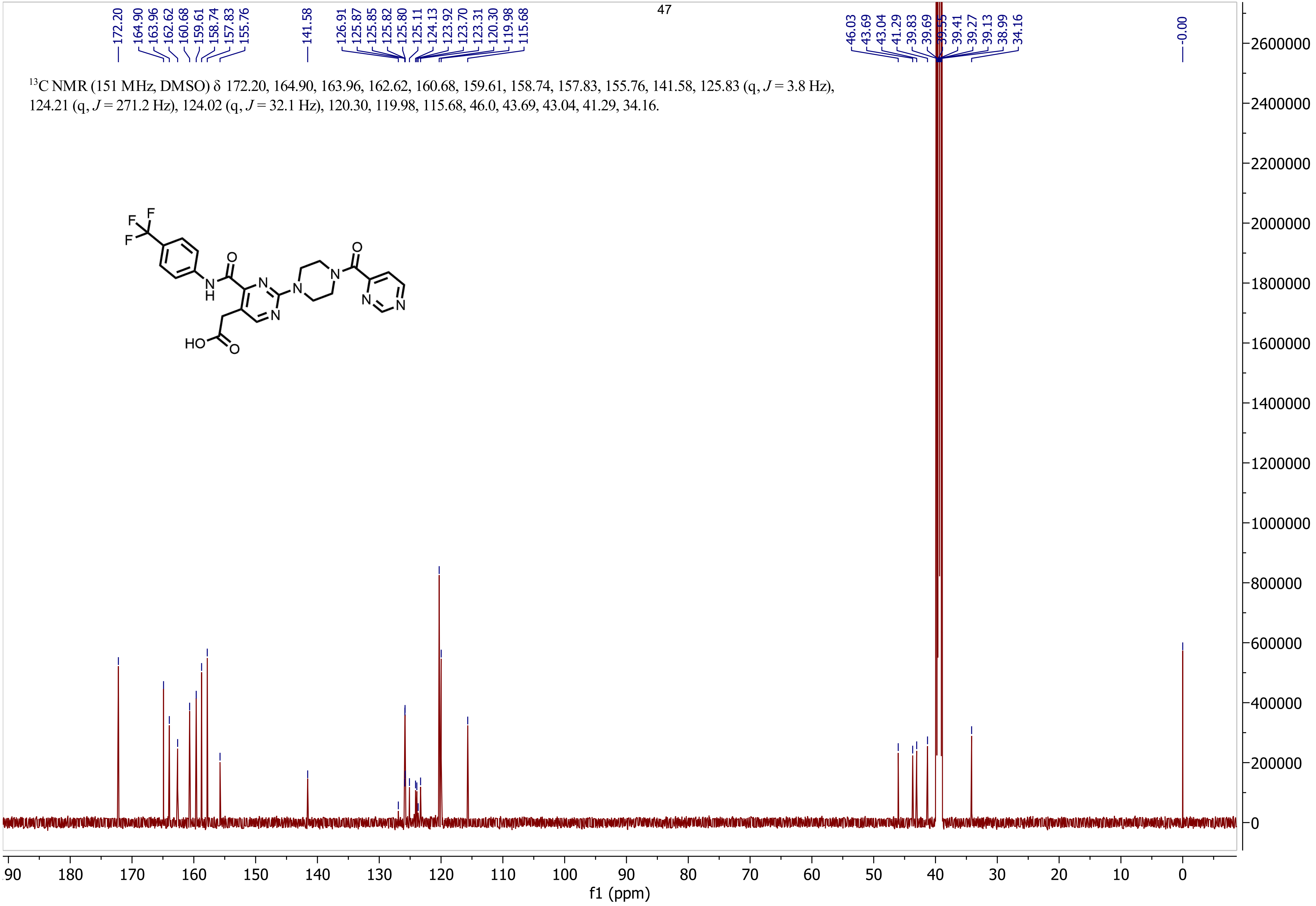

^{1}H NMR of Compound 6

1H NMR (600 MHz, DMSO) δ 10.71 (bs, 1H), 10.15 (s, 1H), 7.91 (d, J = 8.5 Hz, 2H), 7.87 (d, J = 7.7 Hz, 1H), 7.73 (d, J = 8.6 Hz, 2H), 7.67 – 7.61 (m, 2H), 7.44 (d, J = 7.4 Hz, 1H), 7.38 (t, J = 7.6 Hz, 1H), 7.34 – 7.29 (m, 2H), 7.08 – 7.02 (m, 1H), 4.14 (s, 2H), 3.64 (dd, J = 16.1, 8.4 Hz, 1H), 3.53 (p, J = 8.1 Hz, 1H), 3.47 (dd, J = 16.0, 7.4 Hz, 1H), 3.31 (dd, J = 16.2, 8.1 Hz, 1H), 3.25 (dd, J = 16.2, 8.1 Hz, 1H).

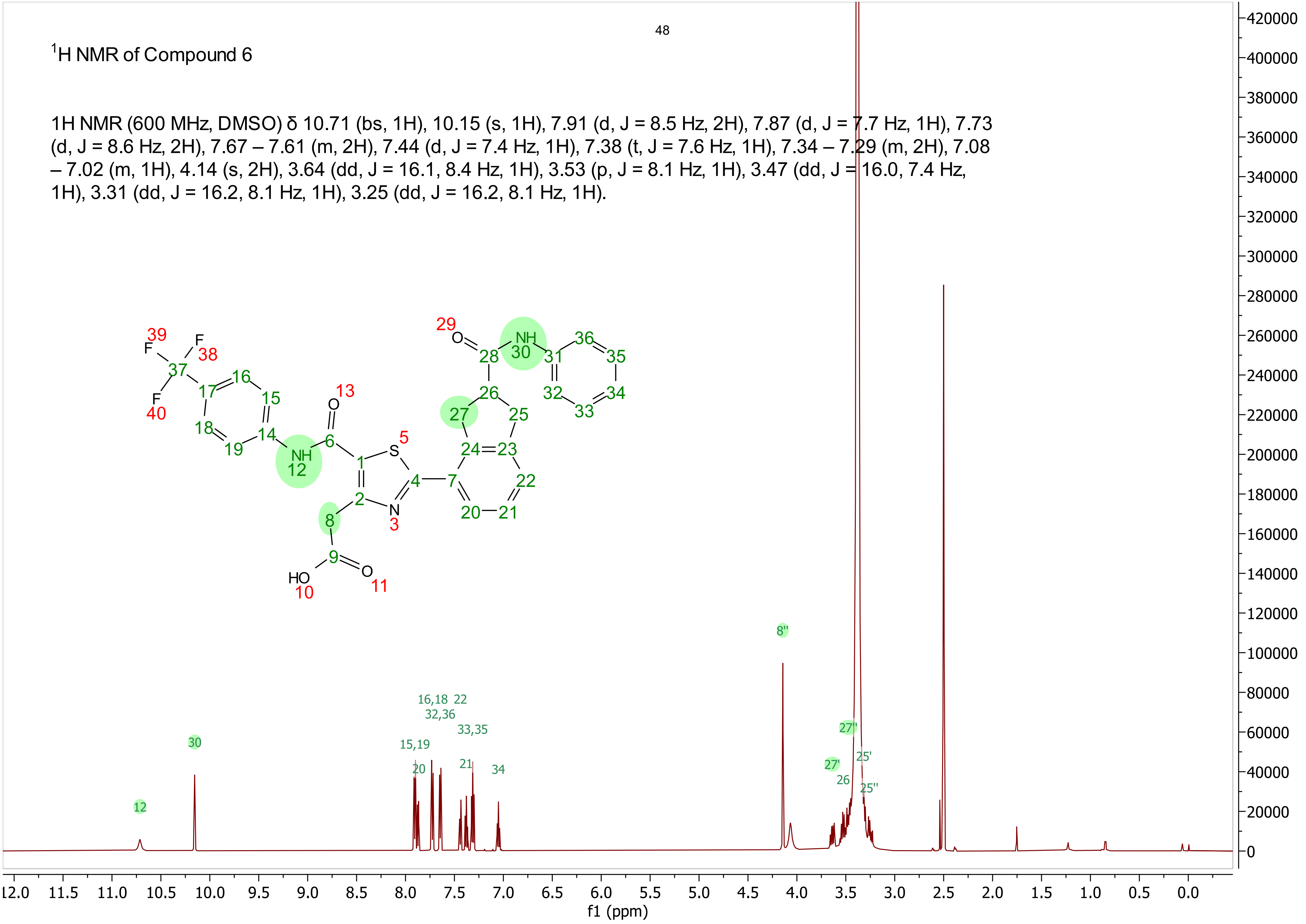

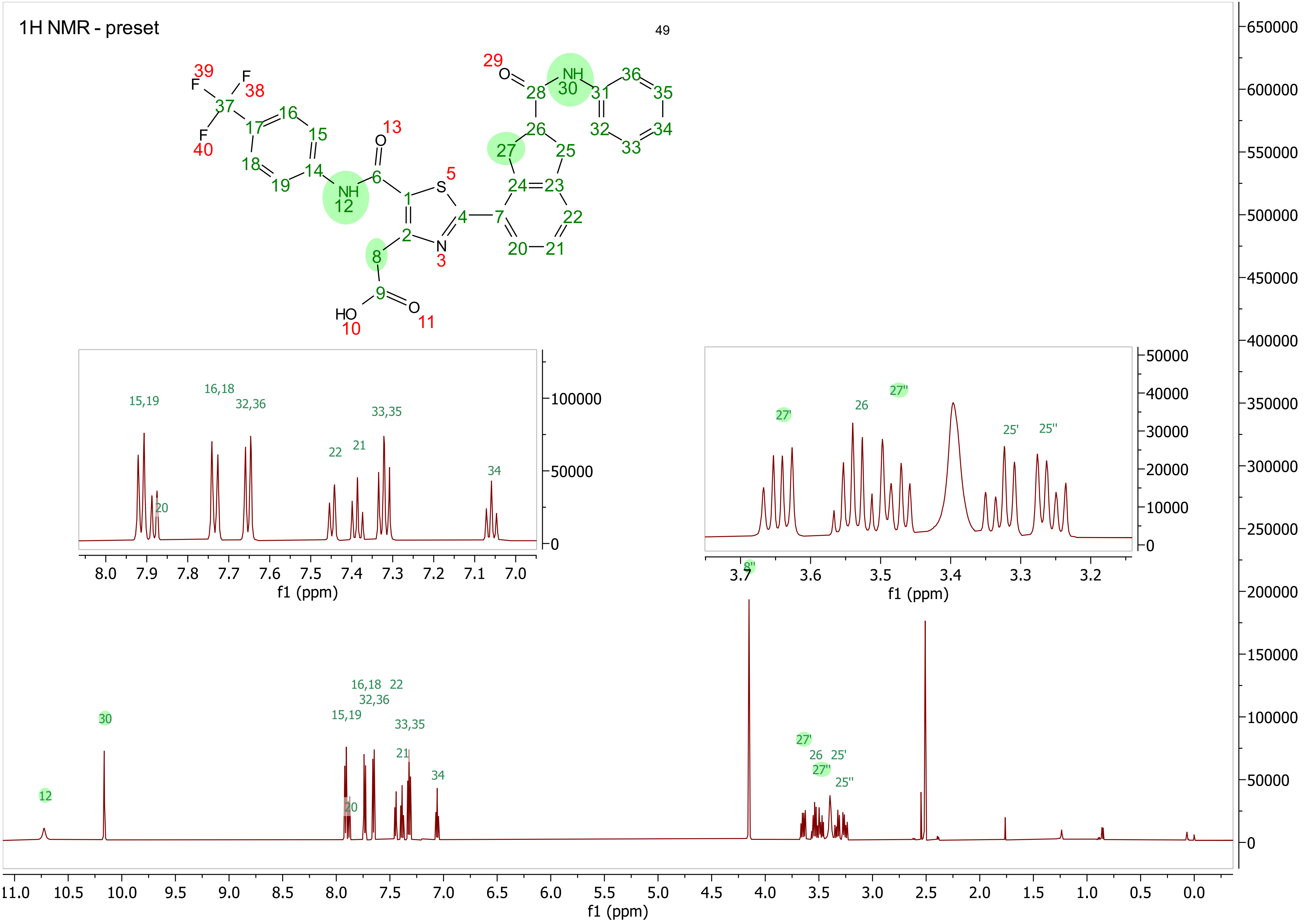

1H NMR - preset
f1 (ppm)
15,19
16,18
32,36
33,35
22
21
20
34
30
12
27'
26
27''
25'
25''
8''

^{13}C NMR of Compound 6

13C NMR (151 MHz, DMSO) δ 173.07, 171.10, 165.96, 159.91, 154.24, 144.35, 142.20, 140.28, 139.28, 128.78, 128.49, 127.65, 126.78, 126.69, 126.02 (q, J = 3.9 Hz), 125.87, 124.35 (q, J = 271.3 Hz), 124.05 (q, J = 32.2 Hz), 123.25, 120.46, 119.22, 44.68, 37.66, 36.80, 36.44.

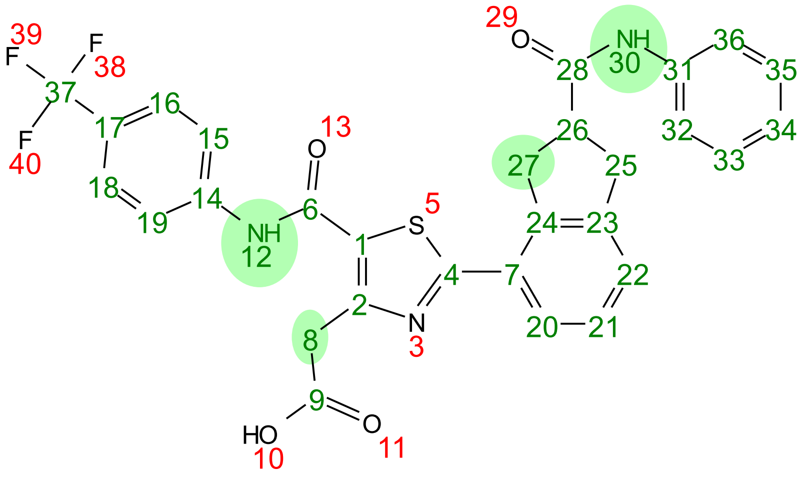

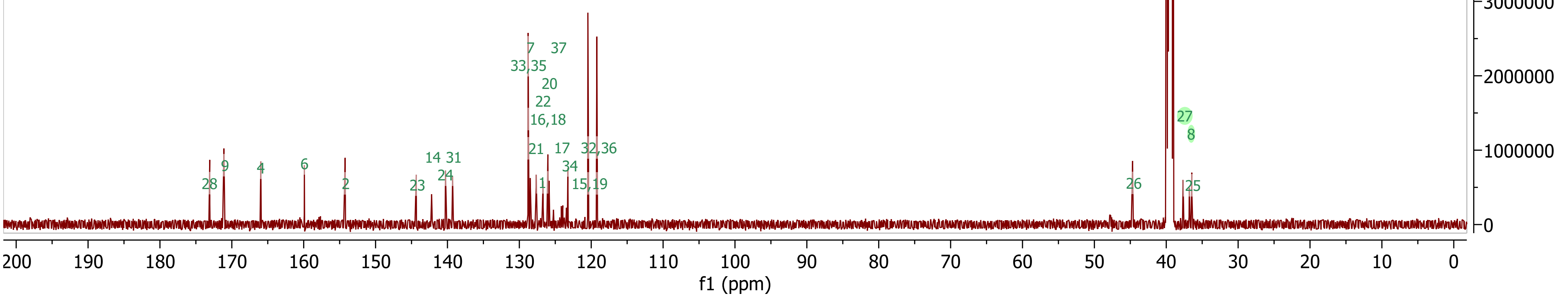

COSY of Compound 6

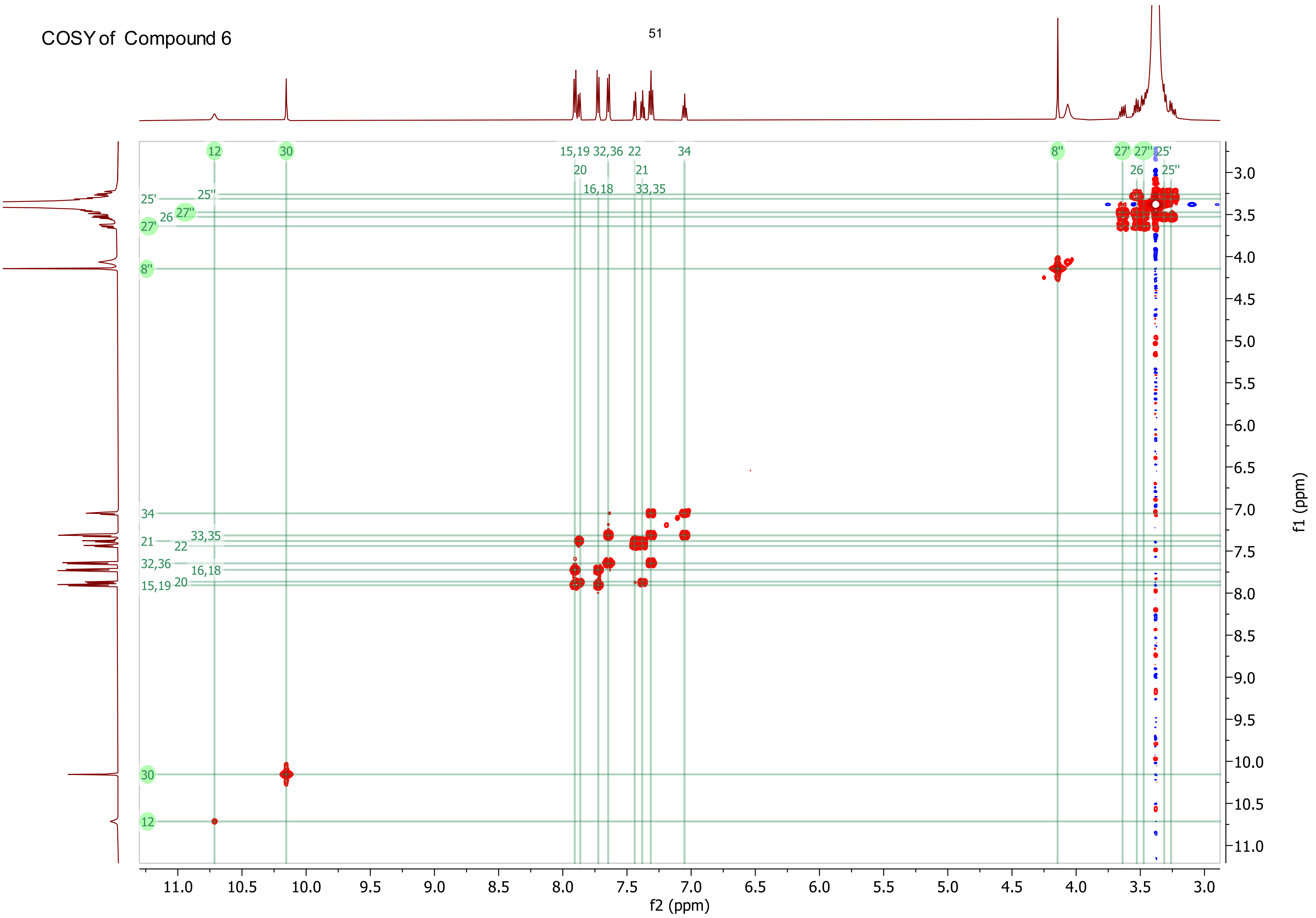

ROESY of Compound 6

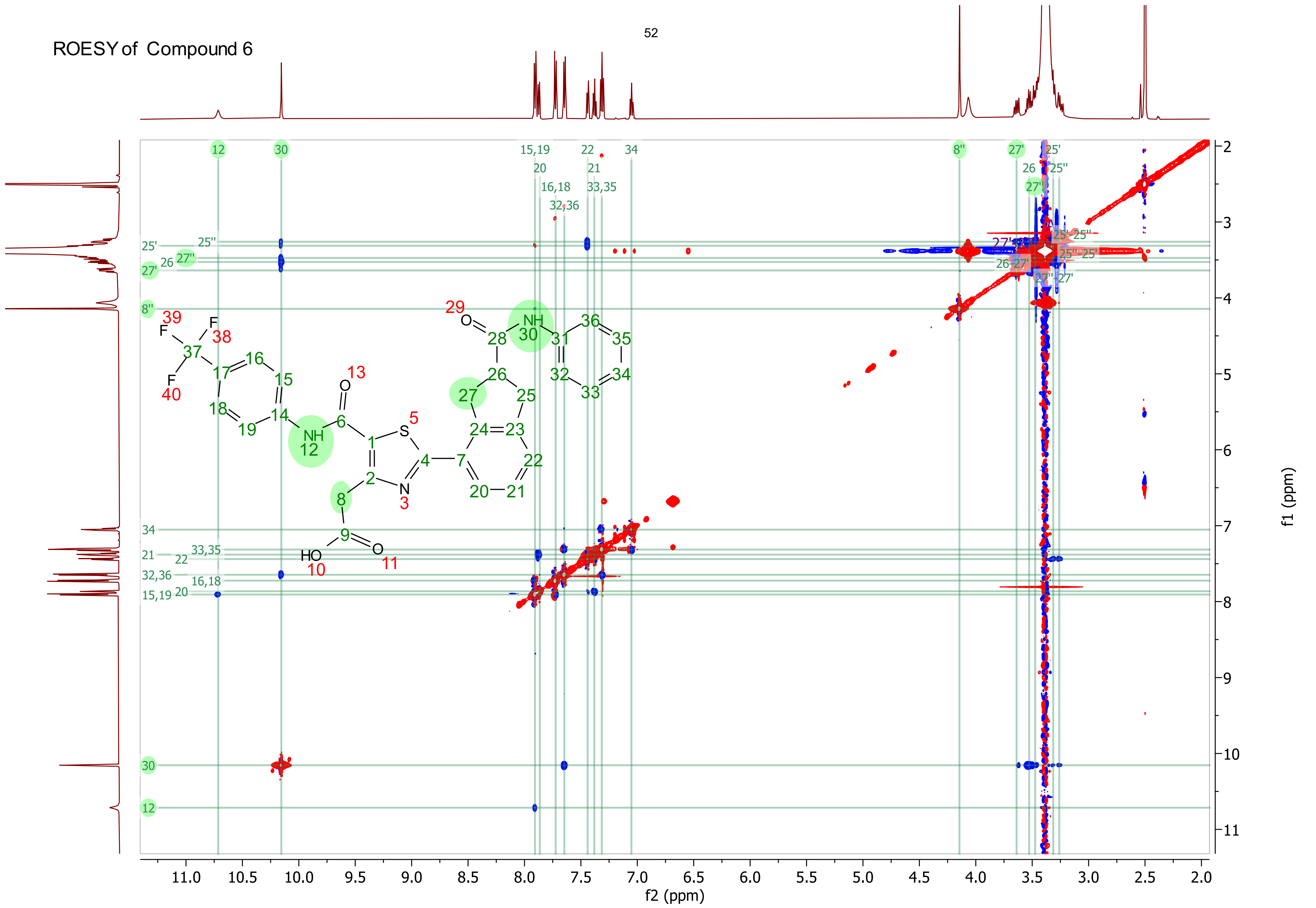

HSQC of Compound 6

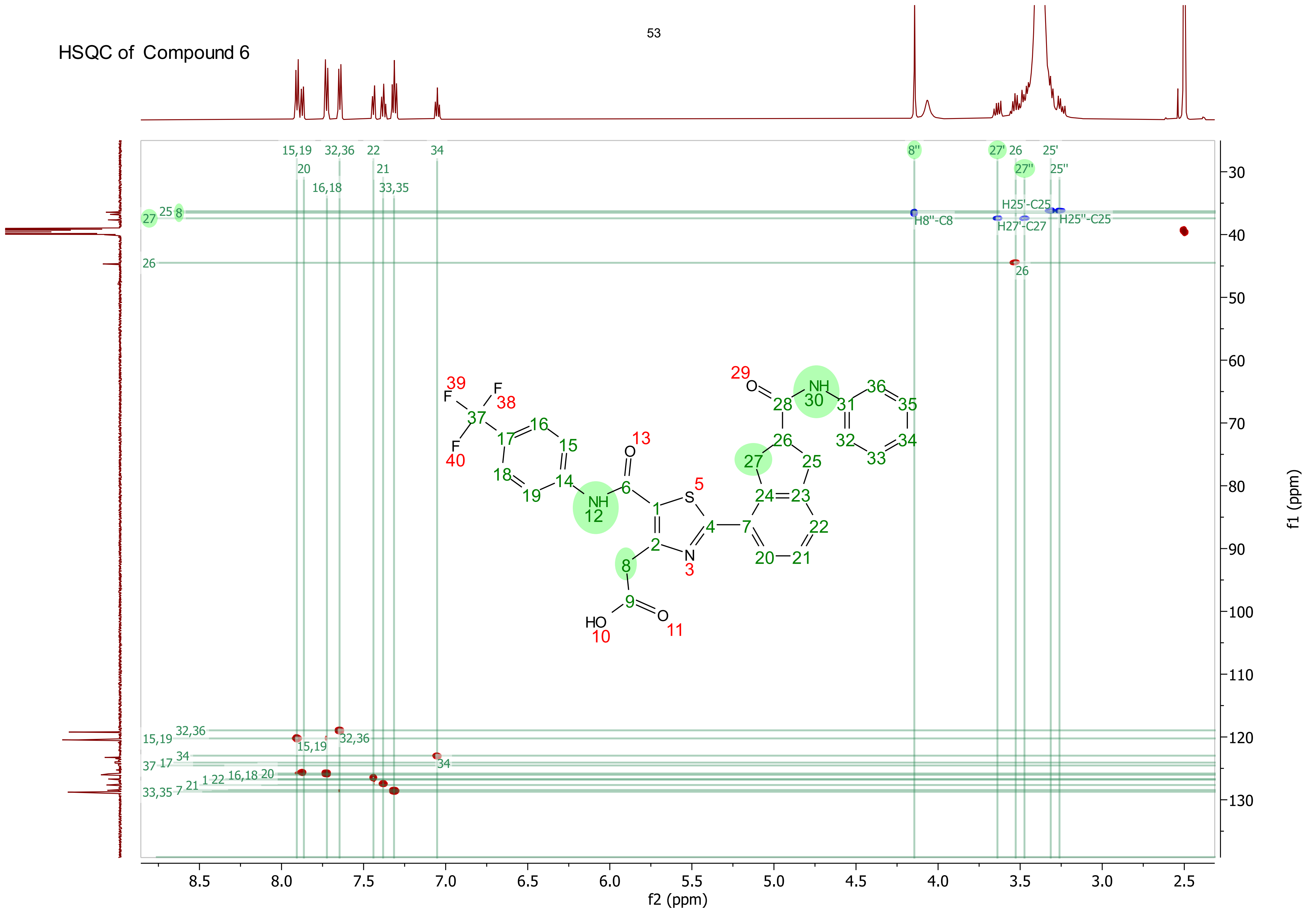

HMBC of Compound 6

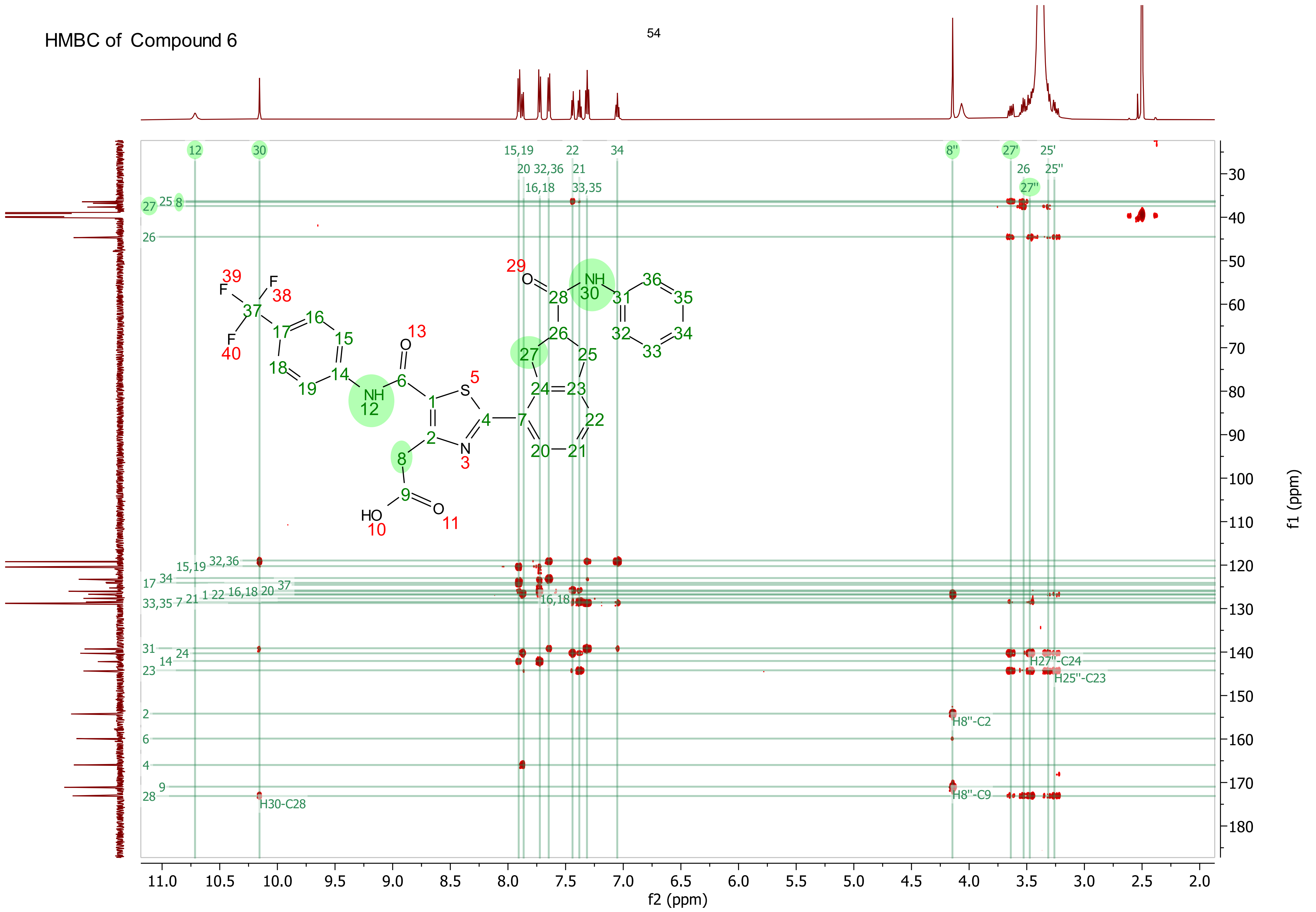

1H NMR of Compound 7 in DMSO-d6

^{1}H NMR (600 MHz, DMSO, sample is a mixture of two rotamers in ratio 53:47) δ 12.4 (bs, 1H), 9.89 (s, 0.53H), 9.85 (s, 0.47H), 7.82 – 7.75 (m, 2H) , 7.66 – 7.49 (m, 6H), 7.48 – 7.32 (m, 6H), 5.25 (p, J = 6.9 Hz, 0.54H), 5.08 (p, J = 6.1 Hz, 0.47H), 3.98 (s, 1H), 3.97 – 3.91 (m, 1H), 3.90 – 3.80 (m, 1H), 3.56 (dd, J = 12.5, 6.5 Hz, 0.53H), 3.50 (dd, J = 11.3, 5.3 Hz, 0.47H), 3.46 – 3.39 (m, 1H), 3.38 – 3.22 (m, 0.47H), 3.12 – 3.07 (m, 0.53H), 3.34 – 3.20 (m, 1H), 2.10 – 2.00 (m, 0.47H), 1.95 – 1.85 (m, 0.53H).

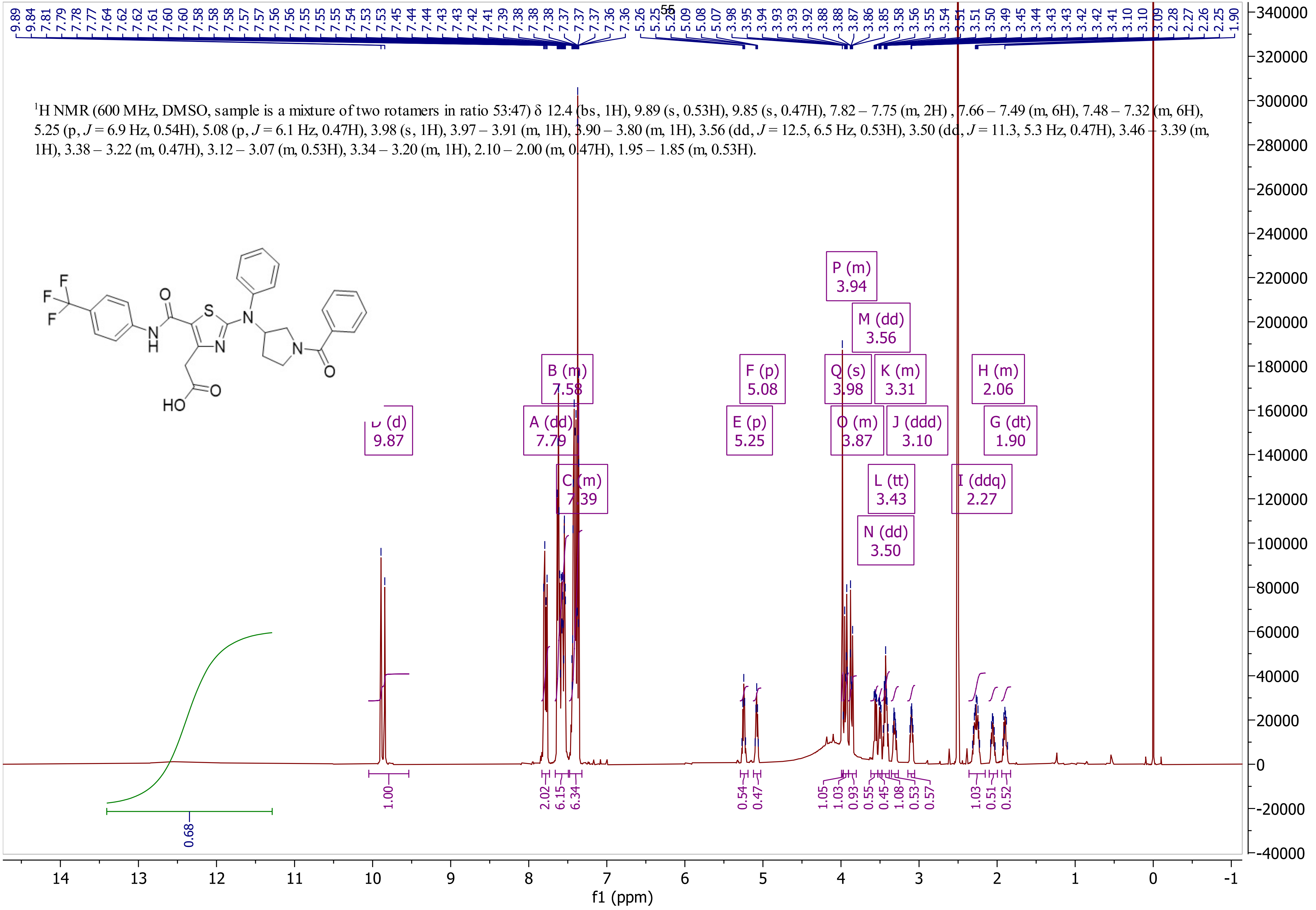

13C NMR of Compound 7 in DMSO-d6

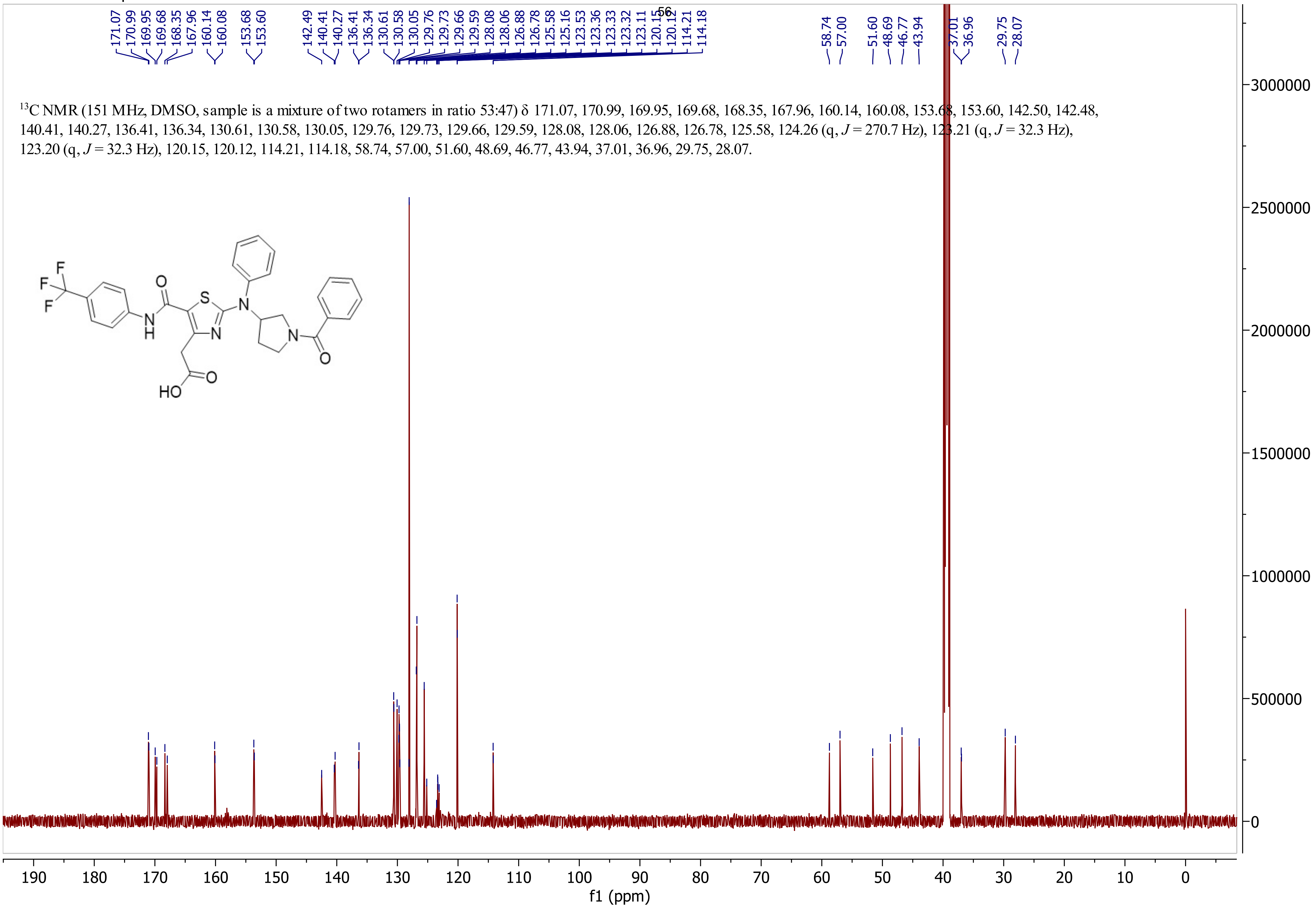

COSY of Compound 7 in DMSO-d6

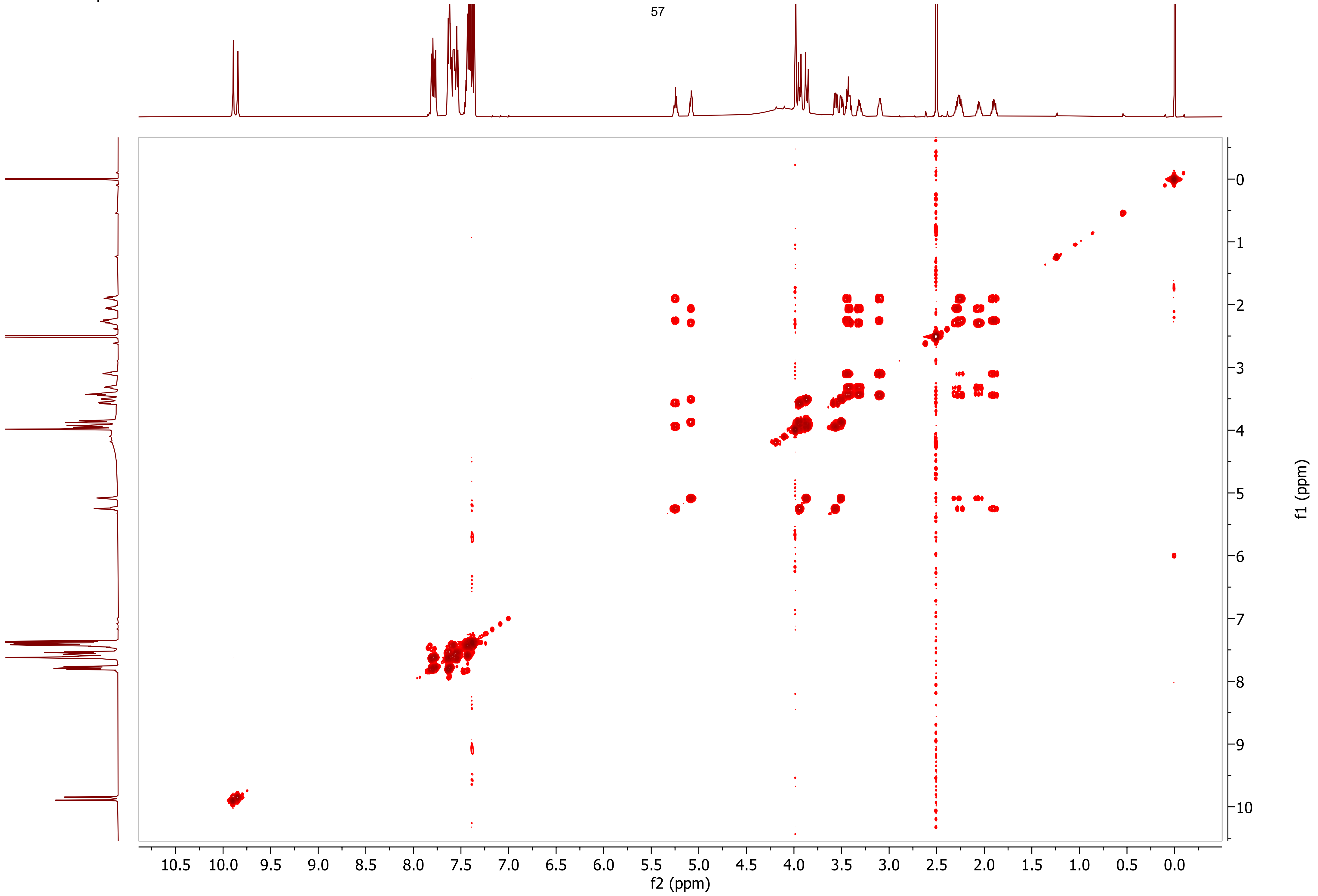

NOESY of Compound 7 in DMSO-d6

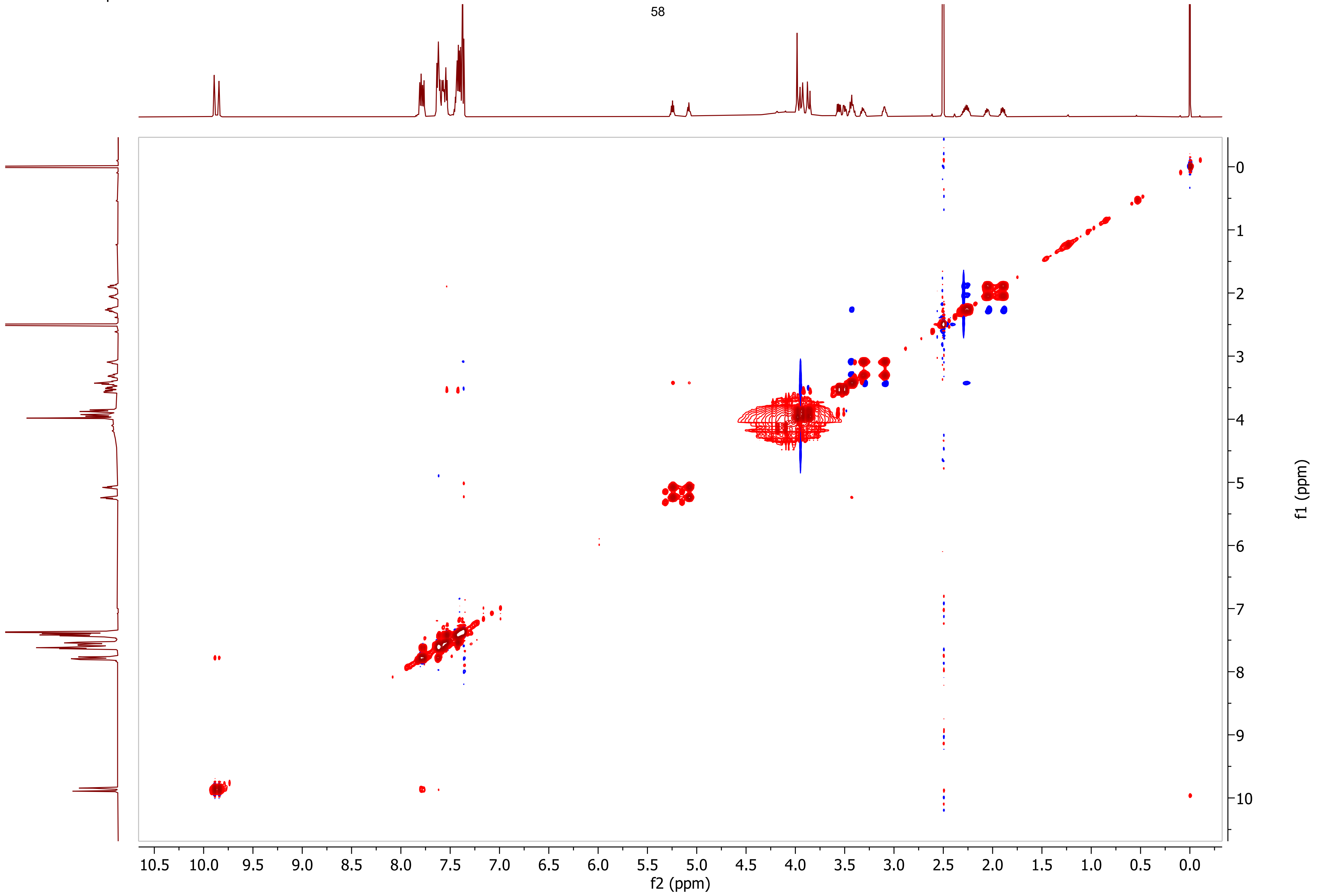

HSQC of Compound 7 in DMSO-d6

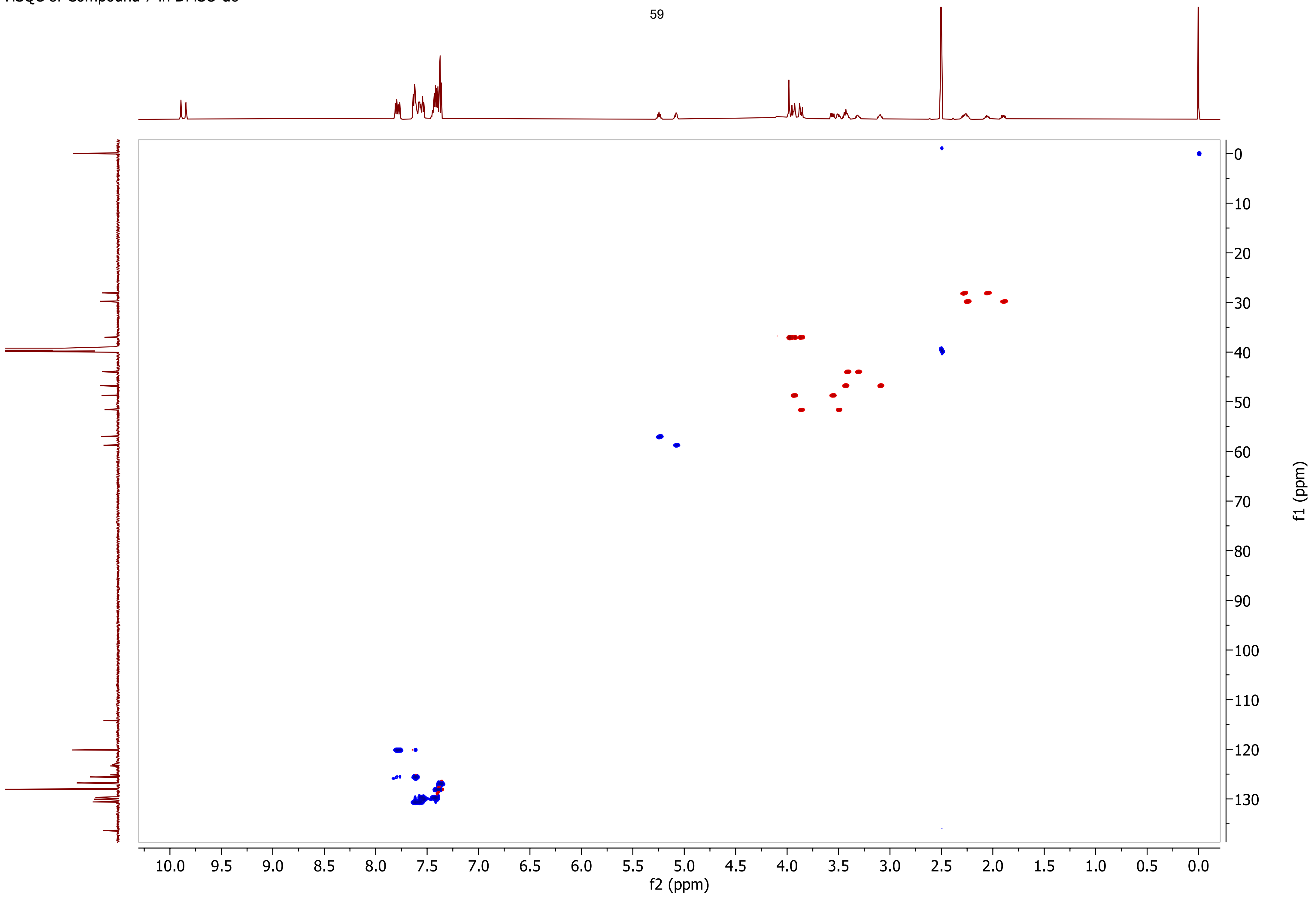

Supplement: Supplementary file 2 — Supplementary Data 1 [file 41467_2025_66768_MOESM2_ESM.pdf]
