# Supplementary material for: WRN structural flexibility showcased through fragment-based lead discovery of inhibitors
Source: Nat Commun. 2026 Jan 3;17:79. doi: 10.1038/s41467-025-66768-8 (PMC12770528; doi:10.1038/s41467-025-66768-8)
Supplement: Supplementary file 3 — Reporting Summary [file 41467_2025_66768_MOESM3_ESM.pdf]

nature portfolio

Corresponding author(s): Daniel Wyss, Sandra Gabelli

Last updated by author(s): Oct 24, 2025

Reporting Summary

Nature Portfolio wishes to improve the reproducibility of the work that we publish. This form provides structure for consistency and transparency in reporting. For further information on Nature Portfolio policies, see our Editorial Policies and the Editorial Policy Checklist.

Statistics

For all statistical analyses, confirm that the following items are present in the figure legend, table legend, main text, or Methods section.

n/a	Confirmed	
	x	The exact sample size (*n*) for each experimental group/condition, given as a discrete number and unit of measurement
	x	A statement on whether measurements were taken from distinct samples or whether the same sample was measured repeatedly
x		The statistical test(s) used AND whether they are one- or two-sided<br>*Only common tests should be described solely by name; describe more complex techniques in the Methods section.*
x		A description of all covariates tested
x		A description of any assumptions or corrections, such as tests of normality and adjustment for multiple comparisons
	x	A full description of the statistical parameters including central tendency (e.g. means) or other basic estimates (e.g. regression coefficient) AND variation (e.g. standard deviation) or associated estimates of uncertainty (e.g. confidence intervals)
x		For null hypothesis testing, the test statistic (e.g. *F*, *t*, *r*) with confidence intervals, effect sizes, degrees of freedom and *P* value noted<br>*Give P values as exact values whenever suitable.*
x		For Bayesian analysis, information on the choice of priors and Markov chain Monte Carlo settings
x		For hierarchical and complex designs, identification of the appropriate level for tests and full reporting of outcomes
x		Estimates of effect sizes (e.g. Cohen's *d*, Pearson's *r*), indicating how they were calculated

Our web collection on statistics for biologists contains articles on many of the points above.

Software and code

Policy information about availability of computer code

Data collection	TopSpin 3.5 pl7

Data analysis

No custom computer codes were developed in in this study.
The software versions used in this study were:
TopSpin 3.5 pl7
Fragment Based Screening Version 1.0
XDS (version Jan 31, 2020)
autoPROC (version 1.1.7)
AIMLESS (version 0.7.7)
CCP4 (7.1.015)
coot (0.9.8.91)
PyMOL Molecular Graphics (2.5.5)
4P (Spotfire; V14.02 & DRC R-Package)
Maestro 13.1 (Schrodinger Suite 2022-1)
Waters Diversity Browser 4.0
MestReNova (14.2.27684)
Biacore Insight Evaluation Software (5.1.18.22102)
Gaussian16 (v16)

For manuscripts utilizing custom algorithms or software that are central to the research but not yet described in published literature, software must be made available to editors and reviewers. We strongly encourage code deposition in a community repository (e.g. GitHub). See the Nature Portfolio guidelines for submitting code & software for further information.

Data

Policy information about availability of data

All manuscripts must include a data availability statement. This statement should provide the following information, where applicable:

- Accession codes, unique identifiers, or web links for publicly available datasets
- A description of any restrictions on data availability
- For clinical datasets or third party data, please ensure that the statement adheres to our policy

All data are provided in the figures, tables, supplemental material and source data files associated with the manuscript.
The final coordinates of WRN have been deposited in the PDB with accession codes 9MJS (AMPPNP), 9MJT (apo Form D), 9MJU (fragment 1), 9MJV (fragment 2), 9MJW (fragment 3), 9MJX (fragment 4), 9MJY (compound 1), 9MJZ (compound 3), 9MK0 (compound 5), and 9MK1 (compound 7). Source data are provided with this paper.

Research involving human participants, their data, or biological material

Policy information about studies with human participants or human data. See also policy information about sex, gender (identity/presentation), and sexual orientation and race, ethnicity and racism.

Reporting on sex and gender

Use the terms sex (biological attribute) and gender (shaped by social and cultural circumstances) carefully in order to avoid confusing both terms. Indicate if findings apply to only one sex or gender; describe whether sex and gender were considered in study design; whether sex and/or gender was determined based on self-reporting or assigned and methods used.
Provide in the source data disaggregated sex and gender data, where this information has been collected, and if consent has been obtained for sharing of individual-level data; provide overall numbers in this Reporting Summary. Please state if this information has not been collected.
Report sex- and gender-based analyses where performed, justify reasons for lack of sex- and gender-based analysis.

Reporting on race, ethnicity, or other socially relevant groupings

Please specify the socially constructed or socially relevant categorization variable(s) used in your manuscript and explain why they were used. Please note that such variables should not be used as proxies for other socially constructed/relevant variables (for example, race or ethnicity should not be used as a proxy for socioeconomic status).
Provide clear definitions of the relevant terms used, how they were provided (by the participants/respondents, the researchers, or third parties), and the method(s) used to classify people into the different categories (e.g. self-report, census or administrative data, social media data, etc.)
Please provide details about how you controlled for confounding variables in your analyses.

Population characteristics

Describe the covariate-relevant population characteristics of the human research participants (e.g. age, genotypic information, past and current diagnosis and treatment categories). If you filled out the behavioural & social sciences study design questions and have nothing to add here, write "See above."

Recruitment

Describe how participants were recruited. Outline any potential self-selection bias or other biases that may be present and how these are likely to impact results.

Ethics oversight

Identify the organization(s) that approved the study protocol.

Note that full information on the approval of the study protocol must also be provided in the manuscript.

Field-specific reporting

[illegible]

Life sciences study design

All studies must disclose on these points even when the disclosure is negative.

Sample size	Sample sizes have been described in the figure legends. All biochemical assays were performed at least N=2, and values reported represent the geometric mean and respective standard deviation. Sample sizes were chosen to provide sufficient statistical rigor to establish the geometric mean and variability of the extracted parameter.
Data exclusions	No data was excluded.
Replication	For ADP glo assays all experiments were performed over at least two independent replicates to ensure reliability. All attempts at replication were successful.
Randomization	Investigators generating and collecting assay data did so without advance knowledge of the underlying SAR nor knowledge of compound structures. Data is collected with respect to compound number nomenclature, and analyzed purely on objective application of a mathematical model to the resultant assay data points. There is no subjective analysis involved in the process, and given the compound nomenclature, limited opportunity for bias in the analysis.
Blinding	Investigators were not blinded during data collection, because it did not involve any animal or human subjects.

Reporting for specific materials, systems and methods

We require information from authors about some types of materials, experimental systems and methods used in many studies. Here, indicate whether each material, system or method listed is relevant to your study. If you are not sure if a list item applies to your research, read the appropriate section before selecting a response.

Materials & experimental systems

n/a	Involved in the study	
☐	☒	Antibodies
☐	☒	Eukaryotic cell lines
☒	☐	Palaeontology and archaeology
☒	☐	Animals and other organisms
☒	☐	Clinical data
☒	☐	Dual use research of concern
☒	☐	Plants

Methods

n/a	Involved in the study	
☒	☐	ChIP-seq
☒	☐	Flow cytometry
☒	☐	MRI-based neuroimaging

Antibodies

Antibodies used	anti-His-6-Peroxidase from mouse IgG1
Validation	The antibody was purchased from commercial sources (Roche 119650850) and no internal validation was performed.

Eukaryotic cell lines

Policy information about cell lines and Sex and Gender in Research

Cell line source(s)	Sf21 cells in Sf-900™ II SFM
Authentication	ThermoFisher Scientific - Cat #11497013. No further authentication was performed, as they were used to produce protein and all information about pure protein is included in manuscript.
Mycoplasma contamination	No (part of the supplier certificate)
Commonly misidentified lines (See ICLAC register)	*Name any commonly misidentified cell lines used in the study and provide a rationale for their use.*

Plants

Seed stocks	*Report on the source of all seed stocks or other plant material used. If applicable, state the seed stock centre and catalogue number. If plant specimens were collected from the field, describe the collection location, date and sampling procedures.*
Novel plant genotypes	*Describe the methods by which all novel plant genotypes were produced. This includes those generated by transgenic approaches, gene editing, chemical/radiation-based mutagenesis and hybridization. For transgenic lines, describe the transformation method, the number of independent lines analyzed and the generation upon which experiments were performed. For gene-edited lines, describe the editor used, the endogenous sequence targeted for editing, the targeting guide RNA sequence (if applicable) and how the editor was applied.*
Authentication	*Describe any authentication procedures for each seed stock used or novel genotype generated. Describe any experiments used to assess the effect of a mutation and, where applicable, how potential secondary effects (e.g. second site T-DNA insertions, mosiacism, off-target gene editing) were examined.*
