# Supplementary material for: WRN structural flexibility showcased through fragment-based lead discovery of inhibitors
Source: Nat Commun. 2026 Jan 3;17:79. doi: 10.1038/s41467-025-66768-8 (PMC12770528; doi:10.1038/s41467-025-66768-8)
Supplement: Supplementary file 5 — Description of Additional Supplementary Data files [file 41467_2025_66768_MOESM5_ESM.pdf]

Title: Supplementary Movie 1
Description: Conformational states and interpolated transitions of WRN. The movie displays the three experimentally observed conformations of the WRN construct, shown sequentially from Form A, Form D, and Form E, and then cycled to illustrate interconversion. Intermediate frames are linear/Cartesian interpolations generated in PyMOL to visualize the transition pathway; actual transient conformations are unknown. Animation playback does not imply measured kinetics or validated transition intermediates; it is intended solely to illustrate plausible movements connecting the three solved states. ATPase D1 in colored in slate blue, ATPase D2 in yellow, flexible linker in red, and zinc binding domain in pink.

Title: Supplementary Data 1
Description: Compound characterization data for all newly synthesized fragments and compounds. This includes HRMS data, LCMS, and 1H NMR and 13C NMR listings and spectra.
